# E- and N-cadherin drive hepatic polarity and lumen elongation via opposing effects on RhoA activity

**DOI:** 10.1083/jcb.202509170

**Published:** 2026-05-27

**Authors:** Junya Hayase, Li Yang, Yu-Heng Zhou, Kangji Wang, Cheng-Ran Xu, Erfei Bi

**Affiliations:** 1 Department of Cell and Developmental Biology, Perelman School of Medicine, University of Pennsylvania, Philadelphia, PA, USA; 2 State Key Laboratory of Reproductive Regulation and Breeding of Grassland Livestock, Institutes of Biomedical Sciences, Inner Mongolia University, Hohhot, China; 3 State Key Laboratory of Female Fertility Promotion, Department of Medical Genetics, School of Basic Medical Sciences, Peking University, Beijing, China; 4 Peking-Tsinghua Center for Life Sciences, Peking University, Beijing, China

## Abstract

Hepatocytes display a unique polarity, forming narrow apical tubes—bile canaliculi (BCs)—between adjacent cells that are essential for liver function. Unlike most epithelial cells, hepatocytes express both E- and N-cadherin, yet their specific roles during BC tubulogenesis remain incompletely understood. Here, we show that these cadherins are collectively required for hepatic polarity and BC formation yet act through distinct mechanisms. E-cadherin localizes to adherens junctions, lateral membranes, and the cleavage furrow, where it promotes division-linked BC elongation and cell–cell contact formation by controlling spindle orientation and RhoA activation via NuMA and ARHGEF17. In contrast, N-cadherin is restricted to adherens junctions and maintains hepatic polarity by attenuating RhoA activity through the p120-catenin family member ARVCF and its partner p190B/ARHGAP5. Together, these findings reveal that dual cadherin expression drives hepatic polarity and BC formation by controlling RhoA activity in a coordinated yet opposing manner.

## Teaser

Hayase et al. show that E-cadherin promotes bile canaliculus elongation via RhoA activation and oriented cell division, while N-cadherin maintains hepatic polarity by attenuating RhoA, defining distinct roles of dual cadherin expression in hepatocytes.

## Introduction

The function of many internal organs such as the kidney, small intestine, and the liver depends on the generation of an epithelium (Buckley and St Johnston, 2022; Garcia et al., 2018). The epithelial tissue consists of epithelial cells that are tightly interconnected through several types of junctional complexes including adherens junctions (AJs), tight junctions, and desmosomes, which enable the tissue to form sheet-like structures (Garcia et al., 2018). Among these junctions, AJs play a principal role in cell–cell adhesion and in the rearrangement and movement of epithelial tissues (Campas et al., 2024; Guillot and Lecuit, 2013; Takeichi, 2014). Epithelial cadherin (E-cadherin) is a key component of the AJ (Takeichi, 2014), and links cell–cell contacts to the circumferential actomyosin network beneath the apical membrane through its binding partners, catenins, thereby contributing to the integrity of epithelial tissues (Campas et al., 2024; Guillot and Lecuit, 2013).

The stereotyped architecture of epithelial cell sheets, however, is disrupted during developmental or pathological processes, including epithelial-to-mesenchymal transition (EMT) (Peglion and Etienne-Manneville, 2024; Yang et al., 2020). In cells undergoing EMT, E-cadherin is downregulated, while neuronal cadherin (N-cadherin), another type of classical cadherin expressed in nonepithelial cells, is often upregulated (Loh et al., 2019; van Roy, 2014). Although both E-cadherin and N-cadherin are involved in cell–cell adhesion, the switch from E- to N-cadherin is associated with changes in cell behavior including motility (Loh et al., 2019) and in cell polarity (Scarpa et al., 2015). Despite extensive studies of these cadherins in different settings including EMT and tumor metastasis (Loh et al., 2019; Radice, 2013; van Roy, 2014), the molecular mechanisms underlying their distinct roles are still lacking.

The liver is the largest internal organ responsible for vital functions, including metabolism, detoxification, blood glucose homeostasis, serum protein synthesis, and bile production (Lotto et al., 2023). These functions of the liver rely on its elaborate architecture formed during development (Lotto et al., 2023; Schulze et al., 2019). Hepatocytes are the epithelial parenchymal cells of the liver and are organized into cords in which two adjacent cells share a narrow apical lumen known as the bile canaliculus (BC). The BC serves as the site of bile excretion (Schulze et al., 2019; Treyer and Musch, 2013). This apical lumen is continuous with that of adjoining hepatocytes and extends throughout the hepatic cords, forming a network of BCs that eventually connect to the bile ducts (Tanimizu and Mitaka, 2017; Treyer and Musch, 2013). The basal surfaces of hepatocytes have the unique feature of being flanked by sinusoidal spaces that contain only sparse connective tissue, allowing hepatocytes access to sinusoidal blood flow. This organization is in marked contrast to the basal membranes of most epithelial cell types, which contact a dense basement membrane (Tanimizu and Mitaka, 2017; Treyer and Musch, 2013). Thus, the elaborate architecture and vital function of the liver are highly dependent on the unique cell polarity of hepatocytes. However, the mechanisms that establish and maintain this polarity, particularly during BC formation and elongation, remain unclear.

Unlike other epithelial cells, hepatocytes express both E- and N-cadherin (Doi et al., 2007; Straub et al., 2011), and their expression is developmentally regulated (Doi et al., 2007). In the mouse liver, both cadherins are present in all hepatoblasts/hepatocytes during embryogenesis, but postnatally, their distribution becomes zonally restricted: E-cadherin is enriched in the periportal vein region with a sharp boundary, where N-cadherin is expressed throughout the liver lobule but is concentrated in the pericentral vein region (Doi et al., 2007). During liver regeneration, hepatocytes that normally lack E-cadherin are induced to express it, resulting in dual cadherin expression (He et al., 2021; Lin et al., 2023; Wang et al., 2024). Thus, the co-expression of E- and N-cadherin is a hallmark of both developing and regenerating hepatocytes. However, whether these cadherins have distinct or overlapping roles in hepatic polarity and BC development remains unknown.

In this study, we show that the rat hepatocyte cell line Can 10 resembles developing hepatocytes (Peng et al., 2006; Treyer and Musch, 2013; Wang et al., 2014), expressing both E- and N-cadherin. While both cadherins are collectively required for hepatic polarity and BC formation, they play distinct roles. E-cadherin promotes BC elongation by coordinating mitotic spindle orientation and localized RhoA activation via nuclear mitotic apparatus (NuMA) (Kiyomitsu and Boerner, 2021) and ARHGEF17. In contrast, N-cadherin maintains hepatic polarity by attenuating RhoA activity through the p120-catenin family member ARVCF (Mariner et al., 2000; Sirotkin et al., 1997) and its partner ARHGAP5/p190B (Cho et al., 2010). Together, these findings reveal a division of labor in which E- and N-cadherin independently yet cooperatively regulate RhoA signaling to drive hepatic polarity and tubular BC formation—processes essential for liver architecture and function.

## Results

### A proper E-cadherin/N-cadherin ratio is critical for hepatic polarity development

**Figure 1.**
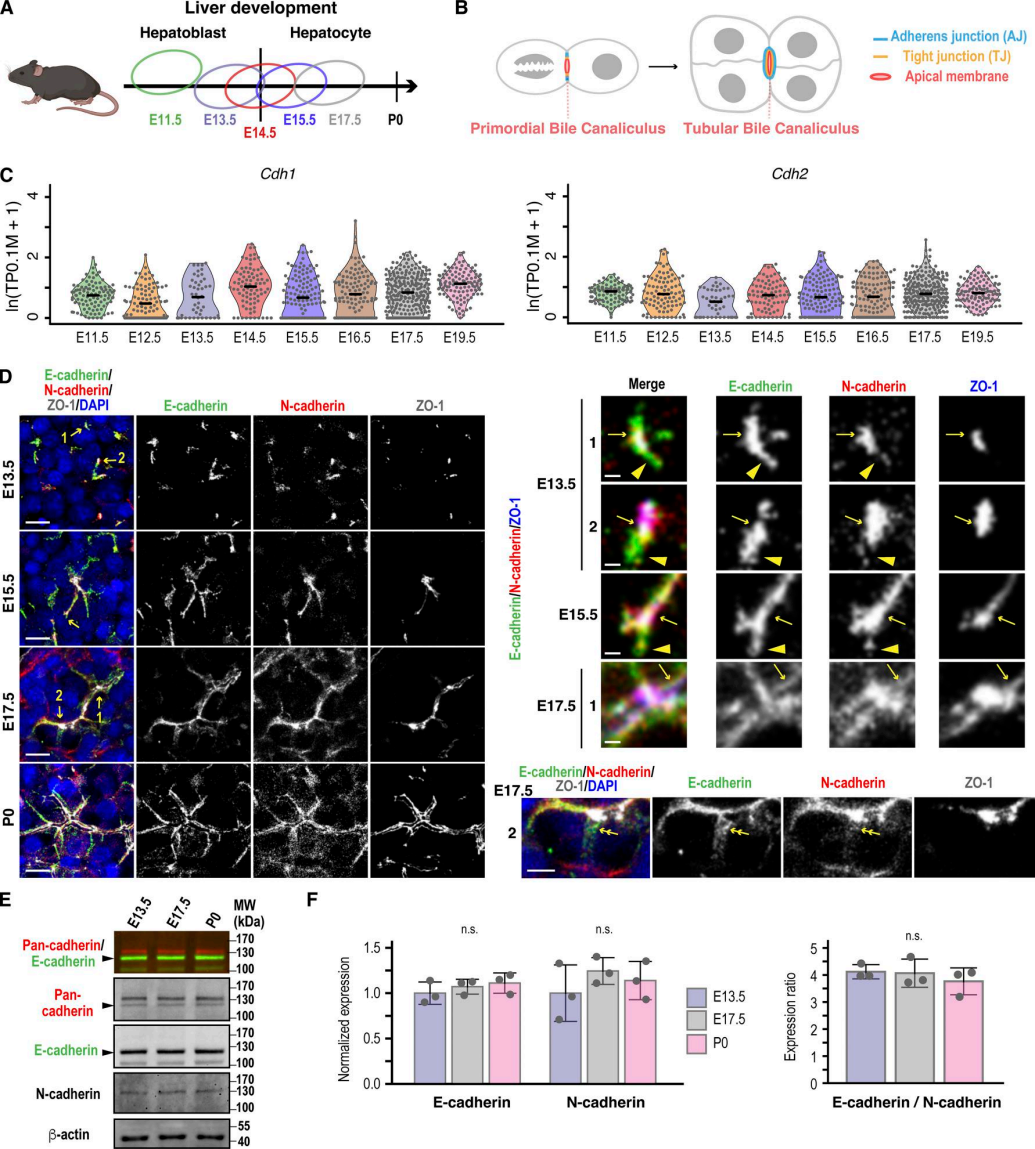
**Dual expression of E-cadherin and N-cadherin in hepatoblasts and hepatocytes during mouse liver development. (A)** Schematic of mouse hepatocyte differentiation from hepatoblasts during liver development. Each circle shows the distribution of the PCA plot at each embryonic day based on the data from scRNA-seq analysis (Yang et al., 2017). Hepatoblast to hepatocyte differentiation occurs around E14.5 (Yang et al., 2017). **(B)** Schematic of tubular BC formation. Oriented cell division of a cell with a primordial BC partitions the preexisting lumen into two daughter cells, resulting in a tubular BC surrounded by three or more cells. **(C)** ScRNA-seq analysis of cadherin genes during mouse liver development. Violin plots represent the expression of CDH1/E-cadherin and CDH2/N-cadherin in hepatoblasts and hepatocytes from the scRNA-seq data (Yang et al., 2017). Each dot represents a single cell. The black line within each violin plot indicates the median expression level. **(D)** Expression of E- and N-cadherin in hepatoblasts and hepatocytes during liver development. Left: liver sections from different developmental stages were immunostained with antibodies against E-cadherin, N-cadherin, and ZO-1, along with DAPI staining. Right: magnified views of the E-cad/N-cad/ZO-1–positive structures indicated by yellow arrows in the left panels. Yellow arrows denote colocalization of E-cadherin and N-cadherin at cellular junctions, whereas yellow arrowheads indicate that E-cadherin extends beyond N-cadherin at these junctions. Double arrows indicate E-cadherin–positive nascent cell–cell contacts between two daughter cells. See also Fig. S1, A–E. **(E and F)** Immunoblot analysis of hepatoblasts and hepatocytes at different developmental stages of the mouse liver. **(E)** Immunoblotting was performed using antibodies against E-, N-, and pan-cadherin, as well as β-actin. Arrowheads indicate E-cadherin corresponding to the lower band of pan-cadherin. MWs of marker proteins are indicated in kDa. **(F)** Left: normalized expression of E- and N-cadherin during BC biogenesis; the values at E13.5 are set to 1.0. Right: expression ratio of E-cadherin to N-cadherin during BC biogenesis after normalizing pan-cadherin antibody affinities to E- and N-cadherin. Data represent means ± SD from three independent samples. Protein expression levels of E-cadherin and N-cadherin, as well as their expression ratio (E-cadherin/N-cadherin), were analyzed by pairwise *t* tests with Holm’s correction. See also Fig. S1, F and G. Scale bars, 1 µm (right panels in D except zoomed 2 at E17.5), 5 µm (zoomed 2 at E17.5 in D), 10 µm (left panels in D). P values are indicated in each graph; n.s., not significant. MWs, molecular weights. Source data are available for this figure: SourceData F1.

**Figure S1.**
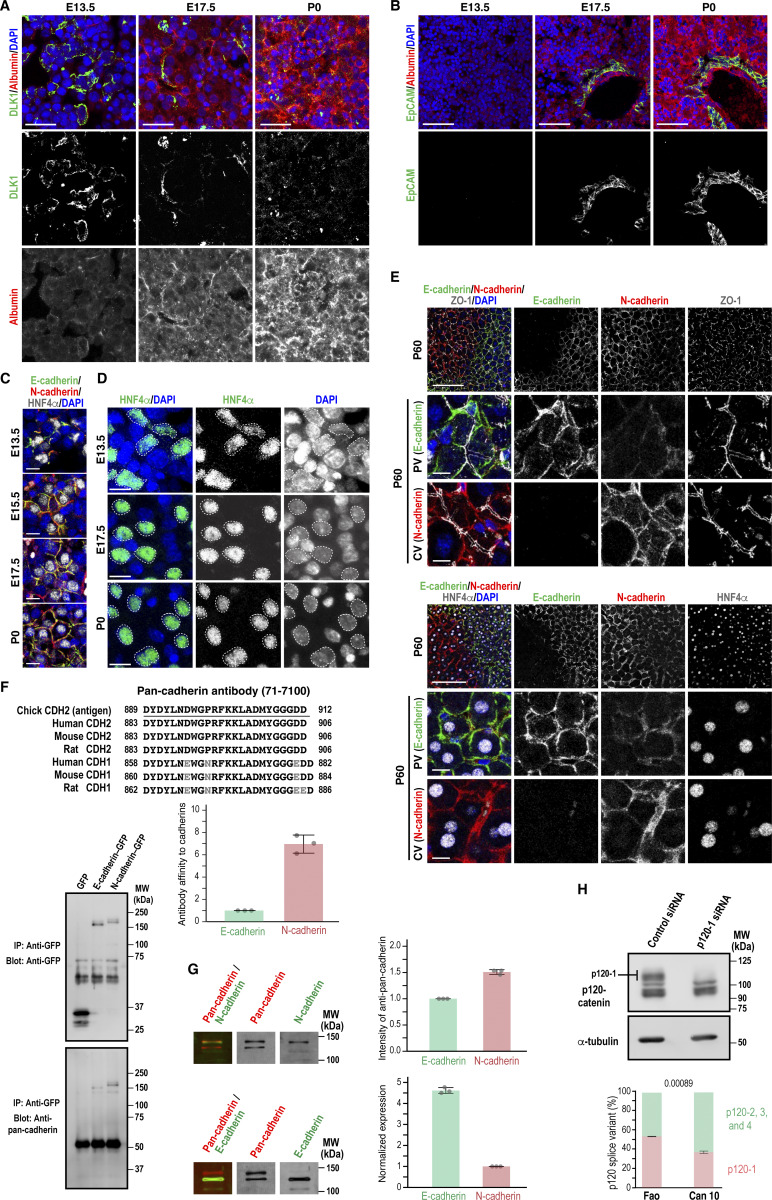
**Expression profiles of DLK1, albumin, EpCAM, HNF4α, E-cadherin, and N-cadherin in hepatoblasts and hepatocytes during mouse liver development, and expression of E-cadherin, N-cadherin, and p120-catenin in Can 10 cells, related to Figs. 1, 2, and 9. (A and B)** Expression patterns of DLK1 and albumin (A) and EpCAM and albumin (B) during mouse liver development. Liver sections from different developmental stages were immunostained with antibodies against DLK1 and albumin (A) or EpCAM and albumin (B), along with DAPI staining. **(C and D)** Expression of HNF4α in mouse hepatoblasts and hepatocytes during liver development. Liver sections from different developmental stages were immunostained using a combination of antibodies against E-cadherin, N-cadherin, and HNF4α, along with DAPI staining (C) or using an anti-HNF4α antibody and DAPI (D). Note that HNF4α localizes within nuclei throughout liver development. **(E)** Expression patterns of E- and N-cadherin in adult hepatocytes. Liver sections from P60 were immunostained with antibodies against E-cadherin, N-cadherin, ZO-1, and HNF4α, along with DAPI staining. Note the different distributions of E-cadherin and N-cadherin along PVs and CVs at P60. **(F)** Relative affinities of the anti-pan-cadherin antibody for E- vs. N-cadherin. Left: lysates from cells expressing GFP-tagged E- or N-cadherin were immunoprecipitated using anti-GFP antibody and analyzed by immunoblotting using anti-GFP and anti-pan-cadherin antibodies. Right: top—antigen information for the anti-pan-cadherin antibody (71-7100) and corresponding regions in E-cadherin/CDH1 and N-cadherin/CDH2. Bottom—pulled-down proteins were normalized to GFP intensity, and the ratio of the pan-cadherin signal to GFP intensity was calculated. The E-cadherin ratio is set to 1.0. Data represent means ± SD from three independent experiments. **(G)** Normalization of E- and N-cadherin expression in Can 10 cells using the anti-pan-cadherin antibody. Top: raw expression data; the intensity of E-cadherin is set to 1.0. Bottom: normalized values calculated using the relative antibody affinities determined in F. Data represent means ± SD from three independent experiments. **(H)** Immunoblot analysis of p120-catenin isoform expression in Can 10 cells. Top: lysates from cells transfected with control or p120-1 siRNA were analyzed using antibodies against p120-1 and α-tubulin. MWs of marker proteins are indicated in kDa. Bottom: quantification of p120-1 vs. other p120 isoforms in Fao and Can 10 cells. Data represent means ± SD from three independent experiments. Scale bars, 10 µm (C and the middle and bottom rows in E), 20 µm (D), 100 µm (A, B, and the top row in E). MWs, molecular weights; PVs, portal veins; CVs, central veins. Source data are available for this figure: SourceData FS1.

To investigate the role of cadherins in AJ assembly and hepatic polarity development, we examined their expression and localization during mouse liver development. Our previous single-cell RNA sequencing (scRNA-seq) analyses revealed that hepatoblasts differentiated into hepatocytes at approximately embryonic day 14.5 (E14.5) (Yang et al., 2017), which was confirmed by immunohistochemistry showing prominent surface expression of the hepatoblast marker DLK1 at E13.5 and significant induction of albumin, a marker of mature hepatocyte differentiation, between E17.5 and postnatal day 0 (P0) (Fig. 1 A; and Fig. S1, A and B). Consistent with a previous report (Doi et al., 2007), we found that both E-cadherin and N-cadherin are expressed in hepatoblasts and hepatocytes from E13.5 to P0, corresponding to the stage of BC formation, elongation, and branching (Fig. 1, B–D; and Fig. S1, C and D). Each hepatocyte expresses both cadherins, which delineate BC structures enclosed by ZO-1 (Fig. 1 D). A closer analysis of the microscopy images revealed extensive overlap between E-cadherin and N-cadherin at cellular junctions (Fig. 1 D, zoomed images). These junctions contained the tight junction protein ZO-1 at their centers, presumably marking the apical membrane initiation sites (Buckley and St Johnston, 2022). Intriguingly, E-cadherin exhibited a broader distribution than N-cadherin at cellular junctions, particularly during early developmental stages (Fig. 1 D; zoomed images of E13.5). Moreover, E-cadherin uniquely localized to nascent cell–cell contacts between daughter cells (Fig. 1 D; zoomed image 2 of E17.5). In contrast, E- and N-cadherin exhibited a zonal distribution after establishment of the BC network (Fig. S1 E) (Doi et al., 2007).

**Figure 2.**
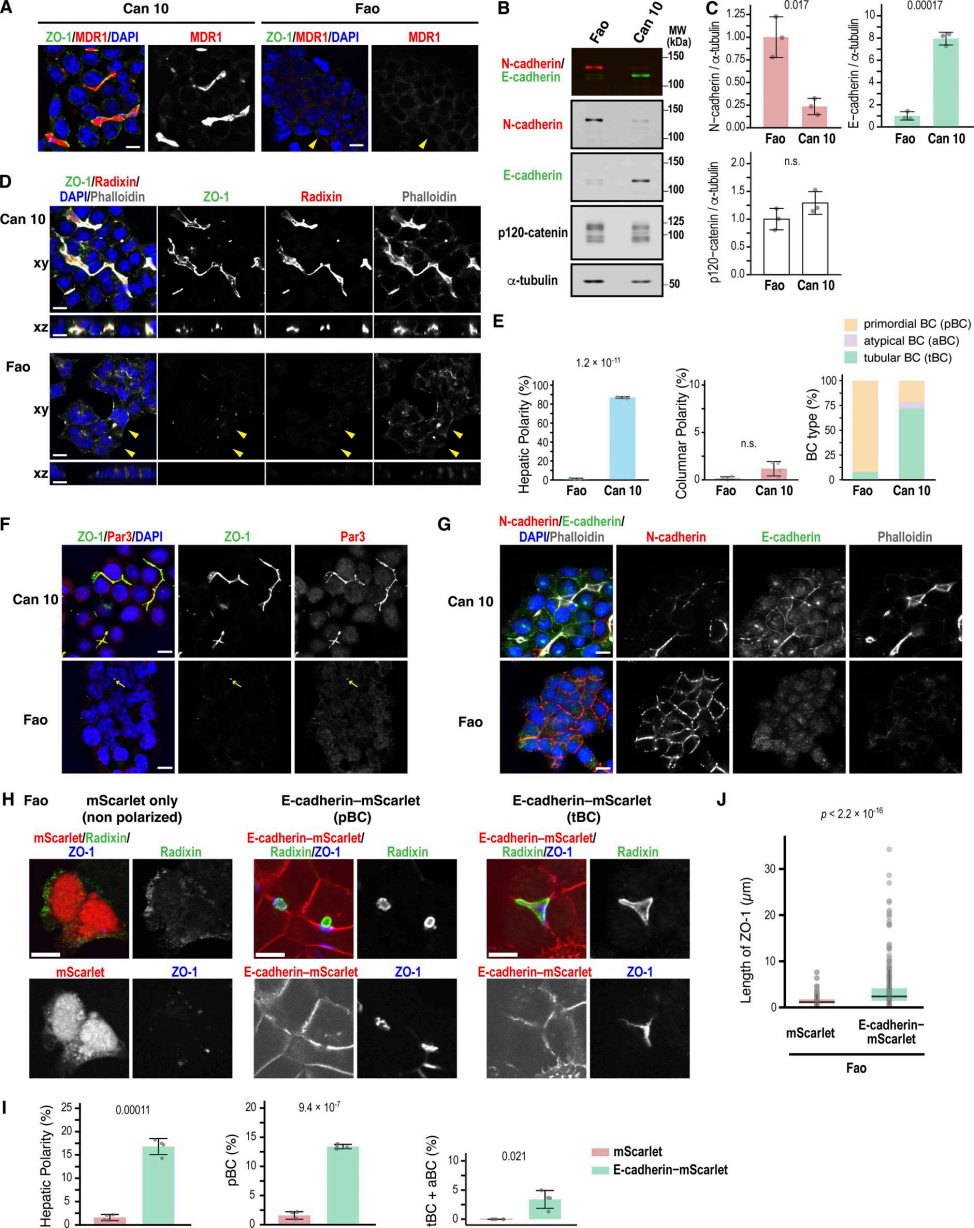
**Increased E-cadherin expression relative to N-cadherin induces hepatic polarity. (A)** Representative iSIM images of polarized Can 10 cells and unpolarized parental Fao cells. Cells were cultured for 3 days, then fixed, and stained with DAPI and antibodies against MDR1 and ZO-1. Yellow arrowheads indicate nonpolarized cells lacking MDR1- and ZO-1–positive structures. **(B)** Immunoblot analysis of E- and N-cadherin expression in Fao and Can 10 cells. Immunoblotting was performed using antibodies against N-cadherin, E-cadherin, p120-catenin, and α-tubulin. MWs of marker proteins are indicated in kDa. **(C)** Normalized intensity ratios of N-cadherin, E-cadherin, and p120-catenin relative to tubulin in Fao and Can 10 cells. Values represent means ± SD from three independent experiments shown in B. Ratios in Fao cells were set to 1.0. See also Fig. S1, G and H. **(D)** Analysis of cell polarization in Can 10 and Fao cells. Cells were cultured for 3 days, then fixed, and stained with phalloidin, DAPI, and antibodies against radixin and ZO-1. iSIM images were shown in horizontal (xy) and orthogonal (xz) views. Note that the Fao cells indicated by yellow arrowheads contain phalloidin-positive structures located within the cells. **(E)** Quantification of Can 10 and Fao cells with various types of polarity and BC structures. Values are means ± SD from four independent experiments (≥401 cells counted per sample). **(F)** Analysis of TJ protein localization and assembly in Can 10 and Fao cells. Cells were cultured for 3 days, then fixed, and stained with DAPI and antibodies against Par-3 and ZO-1. Representative iSIM images are shown. Yellow arrows indicate small TJ-like structures between 2 cells. **(G)** Analysis of AJ protein localization and assembly in relation to F-actin in Can 10 and Fao cells. Cells were cultured for 3 days, then fixed, and stained with phalloidin, DAPI, and antibodies against E-cadherin and N-cadherin. Representative iSIM images are shown. **(H)** Induction of hepatic polarity in Fao cells by increasing E-cadherin expression. Fao cells were cultured for 4 days following transduction with lentiviruses expressing either mScarlet or E-cadherin–mScarlet, then fixed, and stained with antibodies against radixin and ZO-1. Representative confocal images of unpolarized cells expressing mScarlet only, or polarized cells with pBC or tBC expressing E-cadherin–mScarlet, are shown. **(I)** Quantification of Fao cells expressing mScarlet alone or E-cadherin–mScarlet with indicated polarity and BC structures. Values represent means ± SD from four independent experiments (≥154 cells counted per sample). **(J)** Quantification of ZO-1 length in Fao cells expressing mScarlet only or E-cadherin–mScarlet. Values are from four independent experiments (≥211 ZO-1–positive structures counted per condition). Scale bars, 10 µm (A, D, F, G, and H). P values are indicated at the top of each graph. n.s., not significant. TJ, tight junction; pBC, primordial BC; tBC, tubular BC; MWs, molecular weights. Source data are available for this figure: SourceData F2.

Western blotting with E- and N-cadherin–specific and pan-cadherin antibodies showed that hepatoblasts and hepatocytes isolated from E13.5, E17.5, and P0 expressed both cadherins, and their levels and ratios did not vary significantly during these developmental stages (Fig. 1, E and F). Given the relative homogeneity of hepatoblasts and hepatocytes during development (Fig. 1 A) (Yang et al., 2017), it appears that the expression dynamics of E-cadherin and N-cadherin do not significantly affect hepatocyte differentiation itself. These findings raise the question of whether the co-expression of E- and N-cadherin is critical for establishing and/or maintaining the unique hepatic polarity. To address this, we used the rat hepatocyte cell line Can 10, which recapitulates the BC developmental stage observed in vivo (Wang et al., 2014). This cell line expresses MDR1, an apical transporter localized at BCs in hepatocytes, and forms MDR1- and ZO-1–positive tubular structures (Fig. 2 A). We first compared the protein levels of these cadherins between Can 10 cells and their parental Fao cells, which lack hepatic polarity (Fig. 2 A) (Peng et al., 2006). Similar to hepatocytes in developing livers (Fig. 1, D–F), both cadherins were expressed in Can 10 and Fao cells (Fig. 2, B and C). However, E-cadherin was predominant in Can 10, whereas N-cadherin was predominant in Fao (Fig. 2, B and C). Using GFP-tagged E- and N-cadherin to normalize pan-cadherin antibody affinities, we found that the E-cadherin-to-N-cadherin expression ratio in Can 10 cells (∼4.6) closely matched that in hepatoblasts/hepatocytes (∼4.0) from E13.5 to P0, favoring E-cadherin expression (Fig. 1, E and F; and Fig. S1, F and G). The total level of p120-catenin—a binding partner of both E- and N-cadherin—was comparable in Can 10 and Fao cells, with only the difference being the expression of its mesenchymal splice variant, p120-1 (Fig. 2, B and C; and Fig. S1 H) (Keirsebilck et al., 1998; Mo and Reynolds, 1996). Approximately 80% of Can 10 cells exhibited hepatic polarity, defined as a radixin-positive apical domain enclosed by two or more cells (Fig. 2, D and E). More than half of these polarized cells formed tubular BCs involving three or more cells—structures similar to those observed in hepatocytes at E15.5 and E17.5 (Fig. 1 D; and Fig. 2, D and E). In contrast, Fao cells lacked both hepatic polarity and typical epithelial columnar polarity, characterized by apical membranes facing the free surface (Fig. 2, D–G). This was despite their abundant N-cadherin expression and apparent cadherin-mediated cell–cell contacts (Fig. 2 G). The high E-cadherin/N-cadherin ratio and strong polarization capacity of Can 10 cells prompted us to test whether forced E-cadherin expression could induce polarity in Fao cells. Strikingly, E-cadherin–mScarlet expression induced hepatic polarity (Fig. 2, H and I), elongating both ZO-1–positive junctions (Fig. 2 J) and BC structures (Fig. 2, H and I), although the E-cadherin–expressing Fao cells mostly represented primordial BCs and did not fully recapitulate the BC morphology of Can 10 cells (Fig. 2 I). Taken together, in the presence of N-cadherin, upregulating E-cadherin is sufficient to induce hepatic polarity and drive BC formation and elongation. Together, these observations suggest that a proper ratio of E- to N-cadherin in hepatocytes is critical for establishing hepatic polarity.

### Distinct and shared roles of E- and N-cadherin in hepatic polarity and BC formation

**Figure 3.**
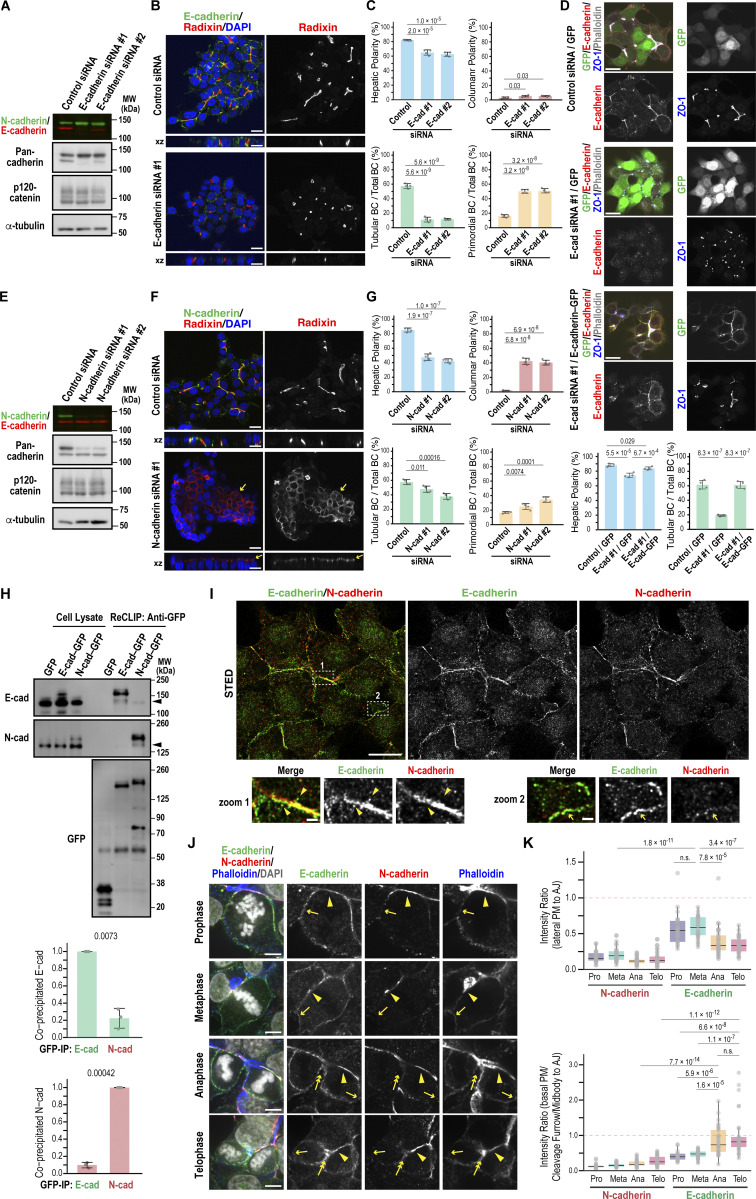
**Distinct roles of E- and N-cadherin in BC elongation and hepatic polarity maintenance. (A–C)** E-cadherin is required for BC elongation. **(A)** Immunoblot analysis of Can 10 cell lysates transfected with siRNAs targeting E-cadherin. MWs of marker proteins are indicated in kDa. **(B)** Representative confocal images of Can 10 cells transfected with E-cadherin siRNAs, cultured for 72 h, and stained with DAPI and antibodies against radixin and E-cadherin. The orthogonal views are shown in xz. **(C)** Quantification of various polarity and BC structures in siRNA-transfected cells. Data represent means ± SD from four independent experiments (≥480 cells per sample). **(D)** Restoration of tubular BC structures by rescue expression of E-cadherin. Can 10 cells were transfected with E-cadherin siRNA #1 one day earlier and then transduced with lentiviruses expressing either GFP or E-cadherin–GFP. Cells were fixed and stained with antibodies against E-cadherin and ZO-1, along with phalloidin. Top: representative confocal images are shown. Bottom: quantification of hepatic polarity and tubular BC structure in the indicated cells. Data represent means ± SD from four independent experiments (≥96 cells per sample). **(E–G)** N-cadherin is required for maintaining hepatic polarity. Experimental procedures and image analyses were as described in A–C, except N-cadherin siRNAs were used. Yellow arrows indicate the apical membrane in columnar polarity cells. Data represent means ± SD from four independent experiments (≥535 cells per sample). **(H)** E- and N-cadherin preferentially form homodimers or oligomers in Can 10 cells. Top: lysates and anti-GFP immunoprecipitates from Can 10 cells expressing GFP-tagged E-cadherin or N-cadherin, treated with the reversible cross-linker DSP, were analyzed by immunoblotting using antibodies against E-cadherin, N-cadherin, or GFP. MWs of marker proteins are indicated in kDa. Middle and Bottom: quantification of coprecipitated endogenous cadherins normalized to pulled-down GFP-tagged E- and N-cadherin, respectively. Data represent means ± SD from three independent experiments. **(I)** Super-resolution imaging of E- and N-cadherin localization. STED microscopy of Can 10 cells cultured for 3 days, fixed, and stained with antibodies against E- and N-cadherin. Lower panels show magnified views of boxed regions. Yellow arrowheads indicate E-cadherin–positive vesicles. Yellow arrows indicate the E-cadherin–positive leading edge of a cell. **(J)** Dynamic localization of E- and N-cadherin during mitosis and cytokinesis. Can 10 cells cultured for 72 h were fixed and stained with phalloidin, DAPI, and antibodies against E- and N-cadherin. Representative confocal images are shown. Arrowheads, arrows, and double arrows indicate AJs, polar cortex, and the basal cleavage furrow or midbody, respectively. See also Fig. S2 D. **(K)** Quantitative analysis of cadherin distribution at the PM during mitosis and cytokinesis. E- and N-cadherin intensities at lateral (top) or basal (bottom) PM regions were normalized to intensity at AJs. Data are from two independent experiments (≥21 cells per condition). Scale bars, 1 µm (lower panels in I), 5 µm (J), 10 µm (upper panels in I), 20 µm (B, D, and F). P values are indicated in each graph; n.s., not significant. MWs, molecular weights. Source data are available for this figure: SourceData F3.

**Figure S2.**
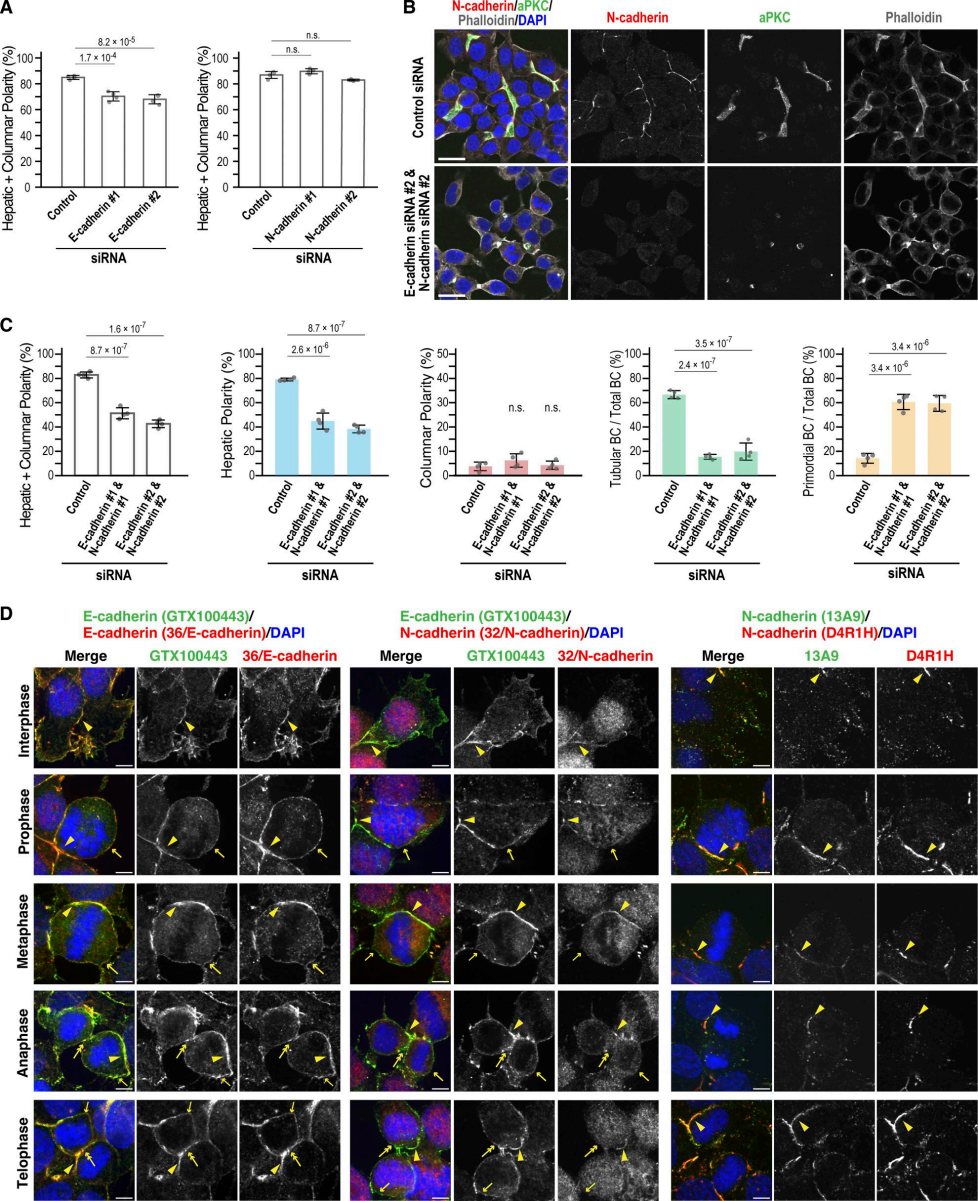
**Shared and distinct roles of E- and N-cadherin in polarity development and BC formation in Can 10 cells, related to Fig. 3. (A)** Quantification of total apical–basal polarity (combined hepatic and columnar polarity) in E- or N-cadherin knockdown cells. Data represent means ± SD from four independent experiments (≥480 cells per condition). **(B)** E- and N-cadherin jointly contribute to AJ assembly and apical–basal polarity. Shown are representative confocal images of Can 10 cells transfected with E- and N-cadherin siRNAs, cultured for 72 h, and stained with DAPI and antibodies against aPKC and N-cadherin. **(C)** Quantification of polarity and BC structures in double knockdown cells. Data represent means ± SD from four independent experiments (≥350 cells per condition). **(D)** Localization patterns of E- and N-cadherin during different stages of cytokinesis. Shown are representative confocal images of Can 10 cells cultured for 72 h, and stained with DAPI and the indicated antibodies. Arrowheads, arrows, and double arrows indicate AJs, polar cortex, and the basal cleavage furrow or midbody, respectively. Scale bars, 20 µm (B), 5 µm (D). P values are indicated at the top of each graph; n.s., not significant.

To define their specific roles in hepatocytes, we depleted E- and N-cadherin individually or together in Can 10 cells using siRNAs. Transient depletion of E-cadherin markedly reduced the formation of tubular BCs (Fig. 3, A–C). In contrast, cells exhibiting hepatic polarity or apical–basal polarity—which includes both hepatic and columnar polarity—showed only a modest but significant reduction (Fig. 3 C; and Fig. S2 A). Most E-cadherin–knockdown cells formed only primordial BCs between two opposing cells (Fig. 3, B and C). The rescue expression of E-cadherin in depleted cells restored the tubular BC architecture (Fig. 3 D), suggesting that E-cadherin is primarily required for BC elongation. N-cadherin knockdown, however, altered cellular geometry, promoting a switch from hepatic to columnar polarity without markedly affecting tubular or primordial BC formation (Fig. 3, E–G). Despite a significant decrease in hepatic polarity, overall apical–basal polarity was maintained in N-cadherin–knockdown cells (Fig. S2 A), indicating that N-cadherin mainly functions to maintain hepatic polarity. Depletion of both E- and N-cadherin in Can 10 cells led to a loss of apical–basal polarity in nearly half the cells (Fig. S2, B and C), a phenotype not observed with individual knockdowns (Fig. S2 A). Notably, some cells failed to maintain cell–cell contacts and detached from clusters (Fig. S2 B). Together, these findings indicate that while E- and N-cadherin cooperate in establishing hepatic polarity and BC formation, presumably by promoting AJ assembly, they play distinct roles in BC elongation and in maintaining hepatic polarity, respectively.

The distinct phenotypes resulting from E- versus N-cadherin knockdown raised the possibility that these cadherins predominantly form homo-oligomers. To test this, we transduced Can 10 cells with GFP-tagged E- or N-cadherin via lentiviral infection and performed reversible cross-link immunoprecipitation (ReCLIP) using the thiol-cleavable cross-linker dithiobis[succinimidyl propionate] (DSP) (Smith et al., 2011). Both GFP-tagged cadherins were expressed at lower levels than their endogenous counterparts (Fig. 3 H). Notably, GFP-tagged cadherins coprecipitated primarily with their respective endogenous forms, though a small amount of E-N hetero-oligomers was also detected (Fig. 3 H). These results support the hypothesis that E- and N-cadherin function mainly as homo-oligomers in Can 10 cells. We next examined cadherin localization using super-resolution stimulated emission depletion (STED) microscopy. E- and N-cadherin colocalized at linear AJs (Fig. 3 I, zoom 1). In addition, E-cadherin appeared as puncta or vesicle-like structures near AJs (Fig. 3 I, zoom 1, arrowheads) and at the leading edge of cells (Fig. 3 I, zoom 2, arrows). A striking difference emerged during cell division (Fig. 3, J and K): N-cadherin remained at AJs surrounding F-actin–decorated BCs (Fig. 3 J, arrowheads), whereas E-cadherin localized not only at AJs (arrowheads) but also at the lateral plasma membranes (PMs), which become the polar cortex of dividing cells (Fig. 3 J, single arrows; and Fig. 3 K). Moreover, E-cadherin was observed at the ingressing basal PM at the cleavage furrow or midbody during cytokinesis, where new cell–cell contacts form (Fig. 3 J, double arrows; and Fig. 3 K). These observations were validated using multiple independent antibodies (Fig. S2 D). Thus, although cadherins localize to AJs, E-cadherin also appears at the lateral membrane and cleavage furrow during cytokinesis. Taken together, these findings suggest that both E- and N-cadherin function primarily as homo-oligomers contributing to AJ assembly, but diverge in their roles in cell division–linked BC biogenesis (Wang et al., 2014).

### E-cadherin and NuMA cooperate during cytokinesis to promote BC elongation

**Figure 4.**
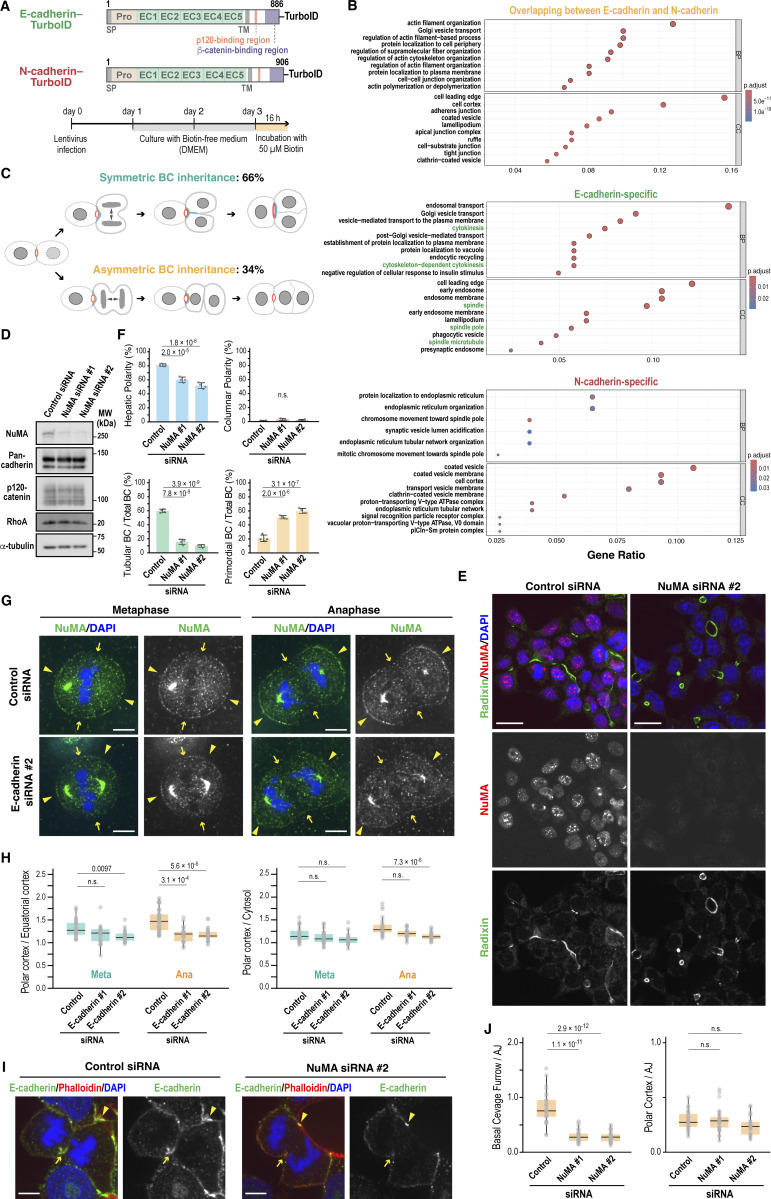
**Interplay between E-cadherin and NuMA is critical for BC elongation. (A)** Schematic of the BioID assay used to identify proteins in proximity to E- and N-cadherin. **(B)** GO analysis of E- and N-cadherin–associated proteins. The top 10 terms in BP and CC are shown. Gene ratio is defined as the number of genes associated with a given term within each gene set (overlapping E-cadherin/N-cadherin, E-cadherin-specific, or N-cadherin-specific) divided by the total number of genes in that gene set. The terms related to spindle and cytokinesis are shown in green. See also Fig. S3, A–C. **(C)** Quantification of symmetric versus asymmetric BC inheritance during cell division. Inheritance patterns were assessed by tracking cells expressing Mzt1-GFP and radixin–mScarlet. Symmetric (emerald green) inheritance and asymmetric (gold) inheritance were quantified in 70 cells from two independent experiments. See also Fig. S4, A and B. **(D)** NuMA knockdown does not alter expression levels of E-cadherin/N-cadherin, p120-catenin, or RhoA. Immunoblot analysis of Can 10 cells transfected with NuMA-targeting siRNAs. MWs of marker proteins are indicated in kDa. **(E)** NuMA is required for BC elongation. Shown are representative confocal images of Can 10 cells transfected with NuMA siRNAs, cultured for 72 h, and stained with DAPI and antibodies against NuMA and radixin. **(F)** Quantification of polarity and BC features in NuMA-knockdown cells. Data represent means ± SD from four independent experiments (≥836 cells per condition). **(G)** E-cadherin knockdown disrupts NuMA localization at the polar cortex. Shown are representative confocal images of Can 10 cells transfected with E-cadherin siRNAs, cultured for 72 h, and stained with DAPI and the anti-NuMA antibody. Arrowheads and arrows indicate polar and equatorial cortex, respectively. **(H)** Quantitative analysis of NuMA localization at the PM during metaphase and anaphase in E-cadherin-knockdown cells. Shown are the ratios of NuMA intensity at the polar cortex to that at the equatorial cortex (left) and to the cytosol (right). Data are from two independent experiments (≥21 cells per condition). **(I)** NuMA knockdown impairs E-cadherin accumulation at the cleavage furrow. Shown are representative confocal images of Can 10 cells transfected with NuMA siRNAs, cultured for 72 h, and stained with DAPI, phalloidin, and an anti-E-cadherin antibody. Arrowheads and arrows indicate AJs and the basal cleavage furrow, respectively. **(J)** Quantitative analysis of E-cadherin distribution at the PM during anaphase in NuMA-knockdown cells. Ratios of E-cadherin intensity at the basal cleavage furrow (left) or polar cortex (right) to its intensity at AJs are shown. Data are from two independent experiments (≥26 cells per condition). Scale bars, 5 µm (G and I); 20 µm (E). P values are indicated in each graph; n.s., not significant. GO, gene ontology; MWs, molecular weights; BP, Biological Processes; CC, Cellular Components. Source data are available for this figure: SourceData F4.

**Figure S3.**
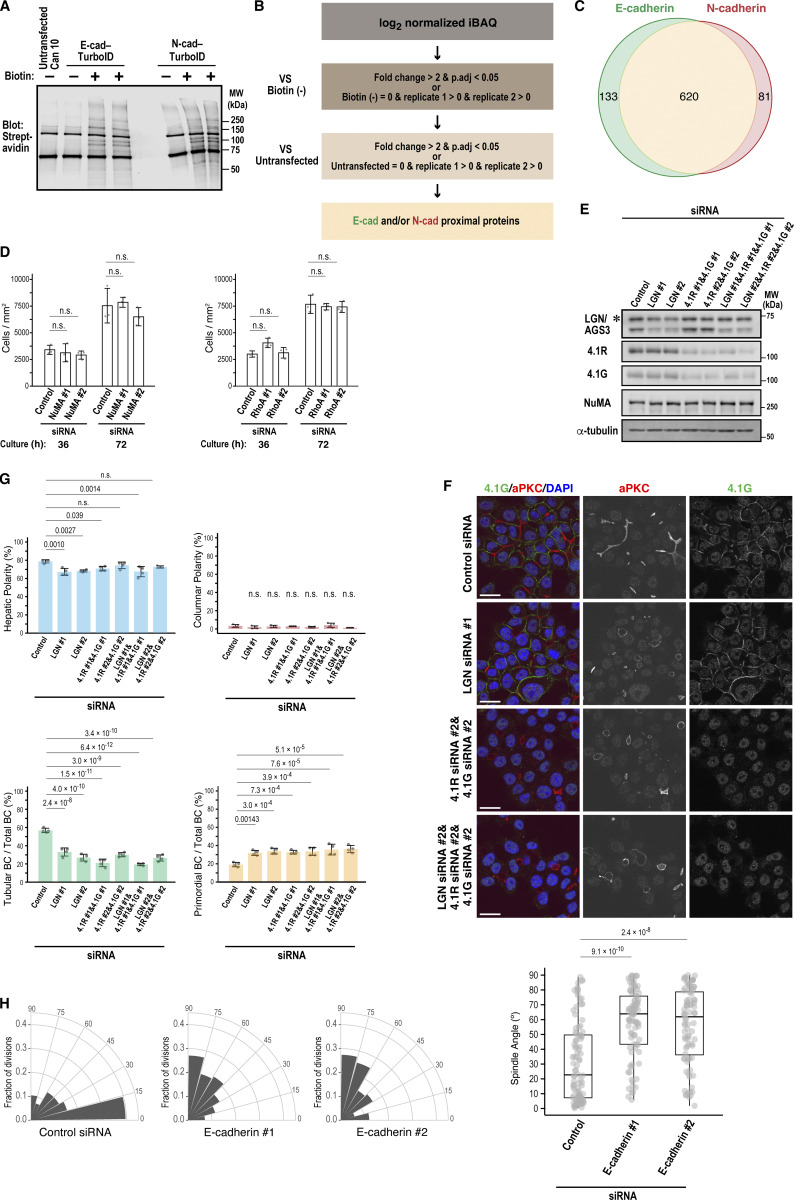
**Identification of E- and N-cadherin interactors, roles of NuMA and its interactors LGN and Band 4.1 in BC elongation, and E-cadherin function in spindle orientation, related to Fig. 4. (A)** BioID analysis of E- and N-cadherin interactors. Streptavidin blot of Can 10 cells expressing TurboID-tagged E- or N-cadherin. MWs of marker proteins are indicated in kDa. **(B)** Analysis scheme for the E- and N-cadherin BioID data. Raw iBAQ values of proteins with ≥2 unique peptides were log_2_-normalized and filtered using two sequential criteria. Proteins were retained if, compared to both negative controls [biotin(−) and untransfected], they showed (1) significant differential expression [adjusted P < 0.05, fold change (FC) >2] or (2) detection in both replicates with complete absence in controls. **(C)** Venn diagram of filtered proteins showing overlap between E-cadherin and N-cadherin, as well as those specific to each. Numbers in the circles indicate overlapping proteins (620), E-cadherin–specific proteins (133), and N-cadherin–specific proteins (81). **(D)** Depletion of NuMA or RhoA does not affect cell proliferation. Can 10 cells transfected with NuMA or RhoA siRNAs were cultured for 36 h or 72 h, stained with DAPI and WGA, and analyzed for cell proliferation. Data represent means ± SD from three independent experiments. **(E)** Knockdown efficiencies of LGN, 4.1R, and 4.1G in Can 10 cells. Lysates from siRNA-transfected cells were analyzed by immunoblotting using antibodies against LGN, 4.1R, 4.1G, and α-tubulin. The asterisk marks the AGS3 isoform of LGN. MWs of marker proteins are indicated in kDa. **(F)** LGN, 4.1R, and 4.1G function in the same pathway to promote BC elongation. Shown are representative confocal images of Can 10 cells transfected with LGN siRNA alone or in combination with both 4.1R and 4.1G siRNAs, cultured for 72 h, and stained with DAPI and antibodies against aPKC and 4.1G. **(G)** Quantification of polarity and BC structures in single and double knockdown cells. Data represent means ± SD from four independent experiments (≥742 cells per condition). **(H)** E-cadherin depletion alters mitotic spindle orientation. Rose diagrams (left) and combined scatter and box-and-whisker plots (right) show spindle angle distributions relative to the BC. Values are from two independent experiments (≥84 cells per condition) and were analyzed using the Wilcoxon rank-sum test with Holm’s correction. Scale bars, 20 µm. P values are indicated at the top of each graph; n.s., not significant. iBAQ, intensity-based absolute quantification; MWs, molecular weights. Source data are available for this figure: SourceData FS3.

To uncover the molecular mechanisms underlying the distinctive roles of E- and N-cadherin, we performed proximity-dependent biotin identification (BioID) using TurboID-tagged E- and N-cadherin constructs in Can 10 cells (Fig. 4 A and Fig. S3, A–C) (Cho et al., 2020). Mass spectrometry analysis showed that 74% (620 out of 834) of the interactome proteins overlapped between E-cadherin and N-cadherin (Fig. S3 C and Table S1), whereas 16% (133 out of 834) and 9.7% (81 out of 834) were specific to E-cadherin and N-cadherin, respectively (Fig. S3 C; and Tables S2 and S3). Gene ontology analysis revealed that actin regulators and junction assembly proteins predominated in the overlapping group (Fig. 4 B and Table S1). This group included the well-known cadherin-binding partners p120-catenin and β-catenin (Fig. 4 A), as well as another interactor, the lipoma preferred partner (Van Itallie et al., 2014), confirming the reliability of our BioID assay. Among the E-cadherin–specific proximal proteins, those involved in vesicle transport and those localized at the leading edge were prominent (Fig. 4 B and Table S2), which is consistent with the presence of E-cadherin at the leading edge (Fig. 3 I and Fig. S2 D) and at PMs forming cell–cell junctions between daughter cells (Fig. 3 J). Intriguingly, cytokinesis-related proteins were also enriched among the E-cadherin–specific proteins (Fig. 4 B and Table S2), suggesting a role of E-cadherin in linking cytokinesis to BC elongation.

**Figure S4.**
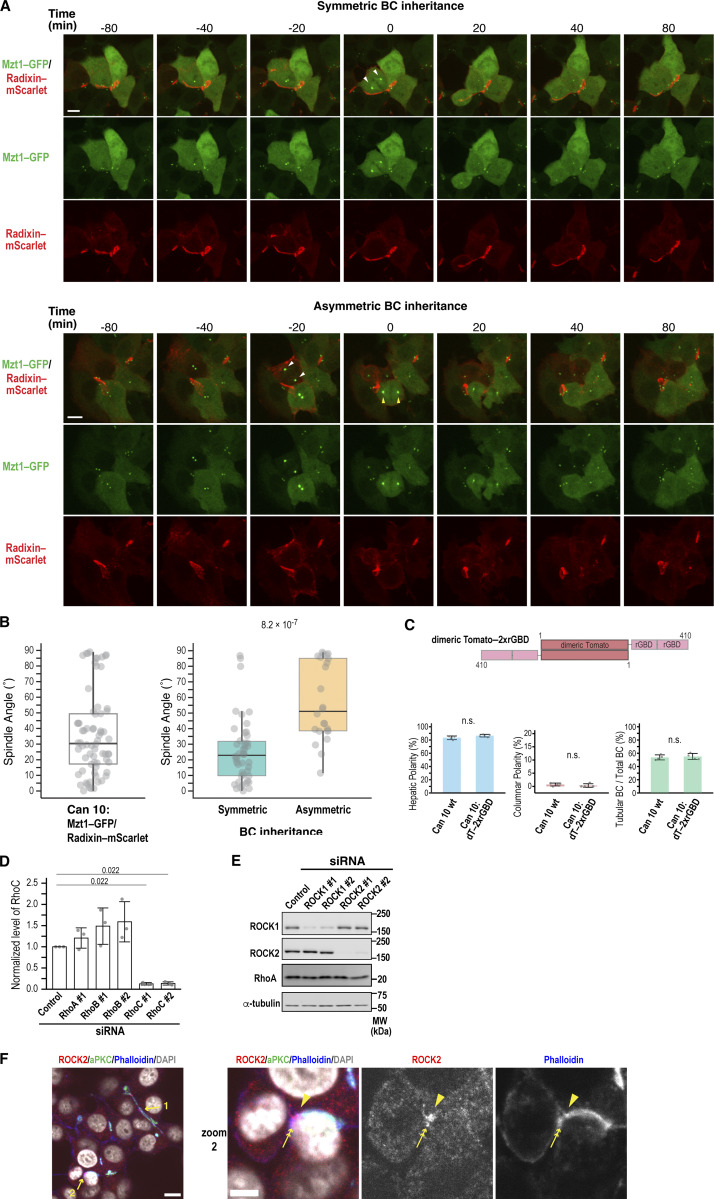
**BC inheritance during cytokinesis, functionality of the Rho biosensor in Can 10 cells, and expression, localization, and knockdown efficiency of Rho and ROCK isoforms in Can 10 cells, related to Figs. 4, 5, and 6. (A)** Montage of time-lapse imaging of Can 10 cells expressing Mzt1-GFP and radixin–mScarlet during cytokinesis. Maximum-intensity projections are shown. The top and bottom panels show symmetric and asymmetric BC inheritance, respectively. White arrowheads at time 0 indicate spindle orientation parallel to the BC, resulting in BC inheritance into both daughters; yellow arrowheads at time 0 indicate spindle orientation perpendicular to the BC, resulting in BC inheritance into only one daughter. Note that Mzt1 brightens during cell division and relocates to the subapical region after cytokinesis. **(B)** Comparison of spindle orientation between symmetric and asymmetric BC inheritance. The combined scatter and box-and-whisker plots on the left show all cells expressing Mzt1-GFP and radixin–mScarlet; the plots on the right show spindle angle distributions relative to the BC for cells with symmetric or asymmetric BC inheritance (*n* = 46 cells for symmetric inheritance; *n* = 24 cells for asymmetric inheritance). Values are from two independent experiments and were analyzed using the Wilcoxon rank-sum test. **(C)** Expression of an active RhoA biosensor (dT-2×rGBD) does not impair polarization or BC formation in Can 10 cells. Top: schematic of the dT-2×rGBD construct. Bottom: quantification of Can 10 cells expressing the biosensor, categorized by polarity type and BC morphology. Data represent means ± SD from four independent experiments (≥268 cells per condition). **(D)** Quantification of RhoC expression. Data represent means ± SD from three independent experiments; ratios of RhoC/α-tubulin in control siRNA-transfected cells from each experiment are set to 1.0. **(E)** Immunoblot analysis of ROCK1- or ROCK2-depleted Can 10 cells. MWs of marker proteins are indicated in kDa. **(F)** Representative confocal images of Can 10 cells, cultured for 72 h, and stained with DAPI, phalloidin, and antibodies against ROCK2 and aPKC. Yellow arrows in the left panel indicate ROCK2 accumulation near the apical edge. Yellow arrowheads and double arrows in the right panels indicate ROCK2 accumulation at the edge of the BC and the midbody region, respectively. Scale bars, 5 µm (zoomed images in F), 10 µm (A and F). P value is indicated at the top of each graph; n.s., not significant. MWs, molecular weights. Source data are available for this figure: SourceData FS4.

Consistent with our previous report (Wang et al., 2014), confocal time-lapse imaging of Can 10 cells expressing the centrosome marker Mzt1–GFP and the apical marker radixin–mScarlet revealed that BC elongation is primarily driven by oriented cell division, which enables symmetric inheritance of the preexisting BC by the daughter cells (Fig. 4 C; and Fig. S4, A and B). Among mother cells with a preexisting BC, 66% showed a predominantly parallel spindle orientation relative to the BC, leading to symmetric BC inheritance by the daughter cells. In contrast, the remaining 34% exhibited mostly “oblique” or “perpendicular” spindle orientation, resulting in asymmetric BC inheritance (Fig. 4 C; and Fig. S4, A and B). Because of this relationship between oriented cell division and BC elongation, we first focused on NuMA protein, which was identified among cadherin-proximal proteins. NuMA is a nuclear matrix protein that regulates spindle orientation (Kiyomitsu and Boerner, 2021) and forms a complex with E-cadherin (Gloerich et al., 2017). During metaphase and anaphase, NuMA translocates from the nucleus to the cell cortex near the spindle poles (Kiyomitsu and Boerner, 2021), suggesting a spatial and temporal overlap with E-cadherin. Strikingly, NuMA knockdown reduced the number of cells with tubular BCs and increased the number with primordial BCs (Fig. 4, D–F), with no change in columnar polarity (Fig. 4 F). Cell growth was unaffected at least 72 h after siRNA transfection (Fig. S3 D), indicating that the observed defect is not due to a general cytokinesis failure. Given that NuMA depletion phenocopied E-cadherin knockdown, we next examined whether E-cadherin influences NuMA recruitment to the cell cortex during cytokinesis. E-cadherin knockdown did not alter overall NuMA recruitment to the PM (Fig. 4, G and H; polar cortex/cytosol), but specifically reduced its accumulation at the polar cortex, particularly during anaphase (Fig. 4, G and H; polar cortex/equatorial cortex). These results suggest that E-cadherin contributes to NuMA enrichment at the polar cortex. NuMA cortical localization is mediated by its binding partners—LGN and protein 4.1 family members (4.1R and 4.1G)—which are known to interact with E-cadherin (Gloerich et al., 2017; Yang et al., 2009). Knockdown of these NuMA partners reproduced the BC phenotypes seen with NuMA or E-cadherin depletion (Fig. S3, E–G), supporting the idea that E-cadherin regulates spindle orientation through the NuMA-LGN/4.1 pathway. Consistently, E-cadherin knockdown disrupted mitotic spindle alignment, which in control cells typically runs parallel to the long axis of the preexisting BC (Fig. S3 H) (Wang et al., 2014). Together, these data indicate that E-cadherin controls BC elongation by regulating spindle orientation via NuMA and its cortical partners. We also explored whether NuMA affects E-cadherin localization. While NuMA knockdown did not impair E-cadherin recruitment to the polar cortex during anaphase (Fig. 4, I and J), it significantly reduced E-cadherin accumulation at the basal region of the cleavage furrow, where new cell–cell contacts form (Fig. 4, I and J). Taken together, these findings suggest that E-cadherin and NuMA functionally cooperate at the polar cortex and cleavage furrow during anaphase and cytokinesis to promote BC elongation.

### RhoA is the primary Rho GTPase involved in BC elongation

**Figure 5.**
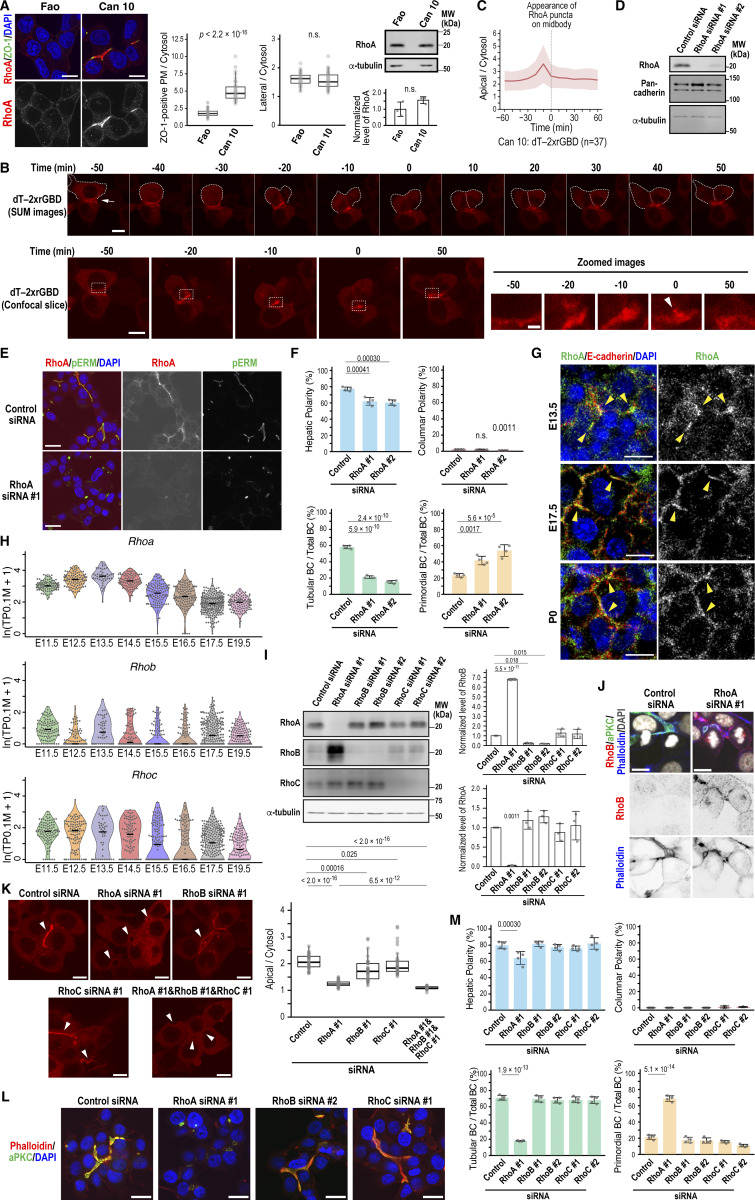
**RhoA is the major isoform responsible for BC elongation. (A)** RhoA accumulates at the BC region in polarized Can 10 cells but not in unpolarized parental Fao cells. Left: representative confocal images of Can 10 and Fao cells cultured for 3 days and stained with DAPI and antibodies against RhoA and ZO-1. Middle: quantification of RhoA intensity at ZO-1–positive PMs (left) or lateral membranes (right), normalized to cytoplasmic intensity. Values are from two independent experiments (≥50 cells per condition). The intensity ratios were analyzed by the Wilcoxon rank-sum test. Right: immunoblot analysis and quantification of RhoA levels in Fao and Can 10 cells. Data represent means ± SD from three independent experiments; RhoA/α-tubulin ratio in Fao cells is normalized to 1.0. Normalized RhoA levels were analyzed using Welch’s *t* test. **(B and C)** RhoA biosensor accumulates at the apical region and cleavage furrow during cytokinesis in cells with a preexisting BC. **(B)** Montage of time-lapse imaging from Can 10 cells expressing dT-2×rGBD during cytokinesis. SUM projection and confocal images are shown. Zoomed views of the boxed regions in the confocal images are also shown (bottom right). The arrow in the SUM image at time −50 indicates a preexisting BC; the arrowhead in the zoomed image at time 0 indicates the midbody. See also Video 1. **(C)** dT-2×rGBD intensities at the BC region over time from cells in (B) (*n* = 37) were quantified and plotted. Bold lines and shaded bands represent the mean and SD, respectively, from two independent experiments, as throughout this study. **(D–F)** RhoA is required for BC elongation. **(D)** Immunoblot analysis of RhoA knockdown in Can 10 cells. MWs of marker proteins are indicated in kDa. **(E)** Representative confocal images of RhoA-depleted Can 10 cells stained with DAPI and antibodies against RhoA and pERM. **(F)** Quantification of polarity and BC features in RhoA-knockdown cells. Data represent means ± SD from four independent experiments (≥593 cells per condition). **(G)** Expression of RhoA in hepatoblasts and hepatocytes during liver development. Liver sections from different developmental stages were immunostained with antibodies against RhoA and E-cadherin, along with DAPI staining. Yellow arrowheads show the colocalization of RhoA with E-cadherin. **(H)** ScRNA-seq analysis of Rho subfamily genes during mouse liver development. Violin plots represent the expression of RhoA, RhoB, and RhoC in hepatoblasts and hepatocytes from the scRNA-seq data (Yang et al., 2017). Each dot represents a single cell. The black line within each violin plot indicates the median expression level. **(I)** Knockdown of RhoA increases RhoB expression. Left: representative immunoblots of cells depleted of RhoA, RhoB, or RhoC. Right: quantification of RhoA and RhoB protein levels. Data represent means ± SD from three independent experiments; Rho/α-tubulin ratio in control siRNA-transfected cells from each experiment is set to 1.0. See also Fig. S4 D. **(J)** Localization of RhoB in RhoA-depleted cells. Shown are representative confocal images of RhoA-depleted Can 10 cells stained with DAPI, phalloidin, and antibodies against RhoB and aPKC. Note that RhoB localizes to the PM and midbody only in RhoA-knockdown cells. **(K)** RhoA is the primary isoform responsible for apical Rho activity in Can 10 cells. Left: representative confocal images of dT-2×rGBD–expressing Can 10 cells transfected with siRNAs targeting Rho subfamily members. Arrowheads indicate BCs. Right: ratios of dT-2×rGBD intensities at the BC vs. cytoplasm in control and Rho-knockdown cells. Data are from two independent experiments (≥32 cells per condition). Values across conditions were analyzed by the Wilcoxon rank-sum test with Holm’s correction. **(L and M)** RhoA is the primary isoform responsible for BC elongation. **(L)** Representative confocal images of Can 10 cells transfected with control siRNA or siRNAs targeting Rho subfamily members. Cells were stained with DAPI, phalloidin, and anti-aPKC antibody. **(M)** Quantification of polarity and BC features in Rho subfamily-knockdown cells. Data represent means ± SD from four independent experiments (≥218 cells per condition). Scale bars, 2 µm (zoomed images in B), 10 µm (A, B, G, J, and K), 20 µm (E and L). P values are indicated in each graph; n.s., not significant. MWs, molecular weights; pERM, phospho-ezrin/radixin/moesin. Source data are available for this figure: SourceData F5.

The unexpected role of NuMA in E-cadherin localization at the cleavage furrow during anaphase prompted us to further explore its link to cytokinesis. A recent study reported that NuMA restricts the localization of the small GTPase RhoA to the cleavage furrow for efficient cell division in HeLa cells (Sana et al., 2022). We found that in interphase, endogenous RhoA accumulated strongly around the BC area demarcated by ZO-1 in Can 10 cells (Fig. 5 A), but was barely detectable at the PM in unpolarized Fao cells, despite similar total RhoA levels (Fig. 5 A). To determine the precise spatiotemporal dynamics of RhoA activity, we expressed a probe specific for its active (GTP-bound) form—dimeric Tomato-tagged tandem repeats of Rhotekin-GBD (dT-2×rGBD)—in Can 10 cells (Mahlandt et al., 2021). Importantly, this probe did not affect hepatic polarization or BC development (Fig. S4 C). Consistent with the antibody staining (Fig. 5 A), the dT-2×rGBD probe showed strong signal intensity surrounding the BC, which appears as a dark space between adjacent cells (Fig. 5 B, time point −50; and Video 1). As the cell progressed through cytokinesis, the probe signal intensified at the BC membrane of the dividing cell, peaking as a punctum at the division site (Fig. 5 B, time point −10; and Fig. 5 C). This punctum likely represents the midbody between daughter cells (Hu et al., 2012). The intensity gradually declined following cleavage furrow ingression (Fig. 5 B, time point 0). This dynamic pattern suggests that active RhoA may contribute to BC maintenance and elongation during cytokinesis.

**Video 1.** **Time-lapse analysis of RhoA activation dynamics during cytokinesis-mediated BC elongation in Can 10 cells (Fig. 5 B).** Can 10 cells expressing the active RhoA biosensor (dT-2×rGBD) and containing a preexisting BC were imaged by spinning disk confocal microscopy. The video shows the dynamics of RhoA activation at the BC and at the division site during cytokinesis-mediated BC elongation. Red fluorescence indicates dT-2×rGBD. Images were acquired at 10-min intervals. Time is displayed as hh:mm, and the movie is shown at 2 frames per sec. Related to Fig. 5 B.

To investigate this hypothesis, we depleted RhoA using siRNAs (Fig. 5 D). Knockdown cells failed to form tubular BCs and instead displayed an increased number of primordial BCs, without an increase in columnar polarity (Fig. 5, E and F). This phenotype essentially phenocopied E-cadherin knockdown (Fig. 3, B–D). Consistent with the findings in Can 10 cells, RhoA accumulated at E-cadherin–positive developing BCs during mouse liver development (Fig. 5 G, arrowheads). scRNA-seq analyses of mouse hepatoblasts and hepatocytes during liver development showed RhoA upregulation between E11.5 and E15.5 (Fig. 5 H) (Yang et al., 2017), coinciding with the period when hepatic polarity is established and BCs begin to elongate (Fig. 1 D). In contrast, other RhoA-related subfamily members, such as RhoB and RhoC, did not show a similar upregulation pattern, although both isoforms were expressed at the RNA level (Fig. 5 H). Consistent with the embryonic hepatoblasts and hepatocytes, both RhoB and RhoC were also expressed in Can 10 cells (Fig. 5 I). However, RhoB appeared to be expressed at very low levels, consistent with the scRNA-seq data (Fig. 5 H), and was barely detectable in Can 10 cells by immunostaining (Fig. 5 J). Strikingly, the protein level of RhoB increased 6.8-fold in RhoA-knockdown cells compared with control cells (Fig. 5 I and Fig. S4 D), and the increased RhoB localized to the PM and midbody (Fig. 5 J). This may explain why RhoA-depleted cells proliferated similar to control cells (Fig. S3 D). We then examined whether RhoA alone accounts for Rho activation at BCs using cells expressing dT-2×rGBD. RhoA depletion dramatically reduced the probe’s intense signal at BCs, whereas depletion of RhoB or RhoC only slightly affected it (Fig. 5 K). Triple knockdown of RhoA, RhoB, and RhoC nearly completely abolished the probe’s signal at BCs (Fig. 5 K). These data indicate that Rho activation at BCs involves all three isoforms, but RhoA accounts for the majority. Importantly, only RhoA knockdown resulted in failure of tubular BC formation (Fig. 5, L and M), despite the 6.8-fold increase of RhoB in these cells. Thus, RhoA is the primary GTPase involved in Rho activation at BCs or AJs, as well as in BC elongation.

### RhoA-ROCK promotes BC elongation and E-cadherin recruitment to the division site during cytokinesis

**Figure 6.**
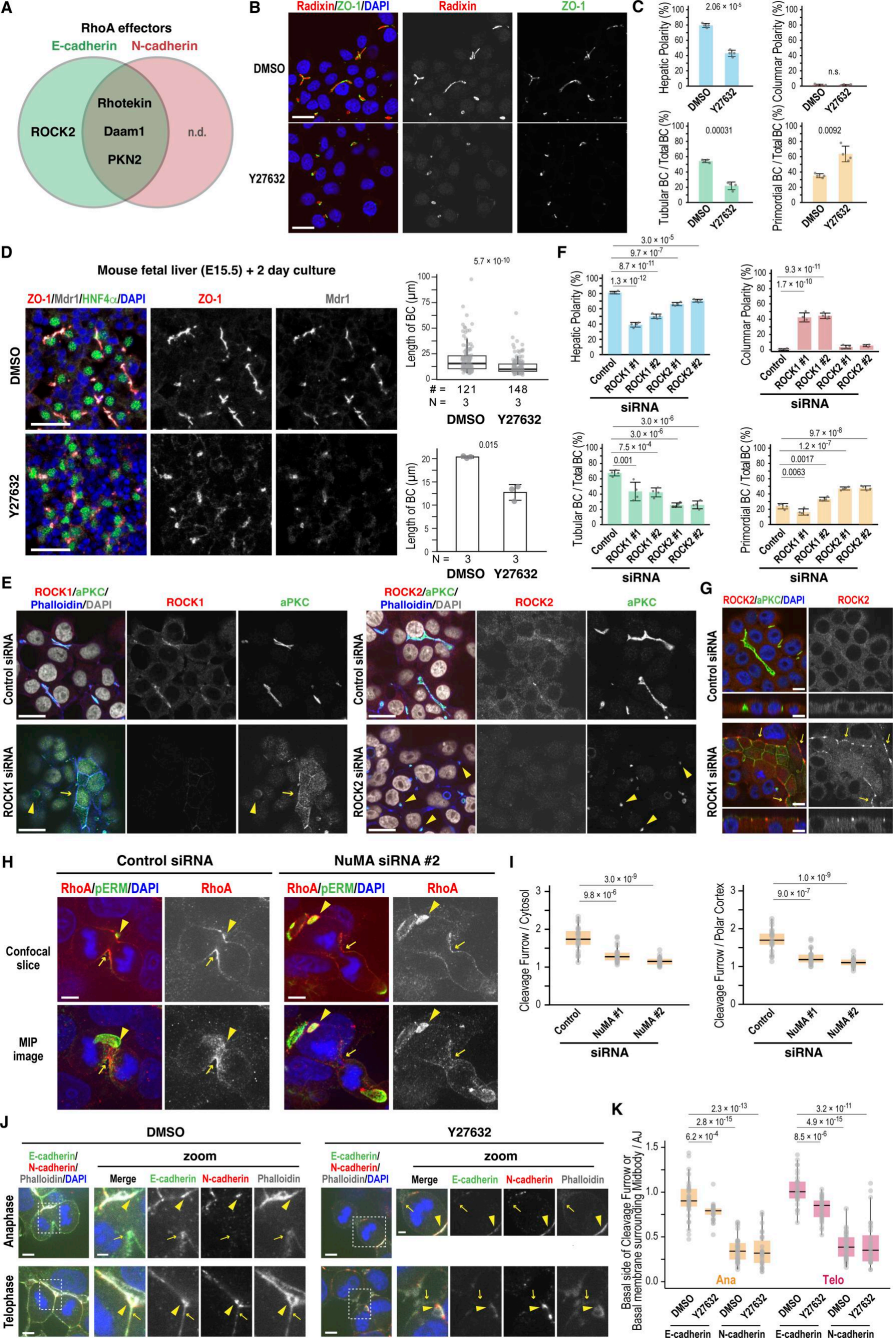
**RhoA-ROCK signaling drives BC elongation and, together with NuMA, promotes E-cadherin accumulation at the division site during cytokinesis. (A)** Venn diagram of RhoA-specific effectors identified by BioID analysis. Note that E-cadherin–specific group contains only ROCK2. n.d., not detected. **(B and C)** ROCK activity is required for BC elongation. **(B)** Confocal images of Can 10 cells treated with the ROCK inhibitor Y27632. Treatment was initiated one day after plating at a concentration of 3 µM and was continued for 48 h. Cells were stained with DAPI and antibodies against ZO-1 and radixin. **(C)** Quantification of polarity and BC features in Y27632-treated cells. Data represent means ± SD from four independent experiments (≥666 cells per condition). **(D)** ROCK activity is required for BC elongation in ex vivo mouse livers. Liver lobes from E15.5 were treated with 1 µM Y27632 for two days and stained with antibodies against ZO-1, Mdr1, and HNF4α. Representative images are shown on the left, and quantitative data for BC length on the right. Top right: combined scatter and box-and-whisker plots of BC length in ex vivo–cultured hepatocytes. # indicates the number of quantified BCs, and N indicates the number of independent experiments. Values between the two groups were compared using the Wilcoxon rank-sum test. Bottom right: mean BC length from three independent experiments. Values between the two groups were compared using Welch’s *t* test. **(E)** Distinct roles of ROCK1 and ROCK2 in hepatic polarity and BC elongation. Shown are representative confocal images of Can 10 cells transfected with siRNAs targeting ROCK1 (left) or ROCK2 (right), cultured for 72 h, and stained with DAPI and antibodies against aPKC and ROCK1 (left) or ROCK2 (right). See also Fig. S4 F. **(F)** Quantification of polarity and BC structures in cells from E. Data represent means ± SD from four independent experiments (≥236 cells per condition). **(G)** Localization of ROCK2 at the apical edge in columnar polarity cells induced by ROCK1 depletion. Shown are representative confocal images of Can 10 cells transfected with ROCK1-siRNA. Cells were cultured for 3 days and stained with DAPI and antibodies against ROCK2 and aPKC. **(H and I)** NuMA is required for RhoA accumulation at the cleavage furrow. **(H)** Confocal images (top) and MIP images of Can 10 cells transfected with NuMA siRNAs, and stained with DAPI and antibodies against RhoA and phospho-ERM. Arrowheads and arrows indicate BCs and the basal cleavage furrow, respectively. **(I)** Quantification of RhoA intensity at the basal cleavage furrow relative to cytoplasm (left) and polar cortex (right). Data are from two independent experiments (≥22 cells per condition). **(J and K)** ROCK activity is required for E-cadherin accumulation at the division site. **(J)** Representative confocal images of DMSO- or Y27632-treated cells stained with DAPI, phalloidin, and antibodies against E-cadherin and N-cadherin. Arrowheads and arrows indicate AJs and the basal cleavage furrow or midbody, respectively. **(K)** Quantification of E-cadherin intensity at the basal cleavage furrow and midbody relative to AJs. Data are from three independent experiments (≥30 cells per condition). Scale bars, 2 µm (zoomed images in J), 5 µm (H and J), 10 µm (G), 20 µm (B, E), 50 µm (D). P values are indicated in each graph; n.s., not significant. MIP, maximum-intensity projection.

Since ROCK2 was the only E-cadherin–specific proximal protein identified among known RhoA effectors (Fig. 6 A and Table S2), we examined its role in BC morphogenesis. Similar to RhoA depletion, treatment of Can 10 cells with the ROCK inhibitor Y27632 significantly reduced tubular BC formation (Fig. 6, B and C). This phenotype was corroborated by ex vivo treatment of mouse fetal liver with Y27632, which impaired BC development (Fig. 6 D). Together, these results indicate that the RhoA-ROCK pathway is essential for BC elongation in vitro and in vivo.

To determine which ROCK isoform is involved in BC elongation, we depleted ROCK1 or ROCK2 in Can 10 cells (Fig. S4 E) and assessed their phenotypes. While depletion of either isoform reduced tubular BCs (Fig. 6, E and F), their primary phenotypes differed: ROCK1 depletion increased columnar polarity, whereas ROCK2 depletion markedly reduced tubular BCs and increased primordial BCs (Fig. 6, E and F). Thus, ROCK2 depletion phenocopied E-cadherin or RhoA depletion. Intriguingly, ROCK2 was primarily cytoplasmic during interphase (Fig. 6 E) but was specifically recruited to the midbody region, as well as to the apical edge of the BC (Fig. S4 F). These observations suggest that ROCK2 functions specifically in BC elongation during cytokinesis. Surprisingly, ROCK2 was aberrantly recruited to the apical edge in columnar cells upon ROCK1 depletion (Fig. 6 G), suggesting that this mislocalization may promote columnar polarity. Taken together, these findings indicate that ROCK2 plays a primary role in BC elongation, while ROCK1 mainly functions in maintaining hepatic polarity.

Given the phenotypic similarities between RhoA and NuMA knockdowns in Can 10 cells and their functional connection during cytokinesis in other cell types (Sana et al., 2022), we examined the effect of NuMA depletion on RhoA localization during anaphase. As expected, NuMA knockdown reduced RhoA recruitment to the basal side of the cleavage furrow (Fig. 6, H and I). Considering the role of NuMA in E-cadherin localization at the cleavage furrow (Fig. 4, I and J), these findings raise the possibility that NuMA regulates E-cadherin localization by confining RhoA-ROCK to the cleavage furrow. To test this, we treated cells with Y27632 and examined E-cadherin localization during anaphase and telophase. Notably, this treatment reduced E-cadherin recruitment to the daughter cell interface at both stages, without affecting N-cadherin localization (Fig. 6, J and K). These results suggest that ROCK activity is required for E-cadherin recruitment to the newly forming cell–cell contacts, thereby facilitating sealing and elongation of a preexisting BC.

### ARHGEF17 drives BC elongation as the key RhoGEF

**Figure S5.**
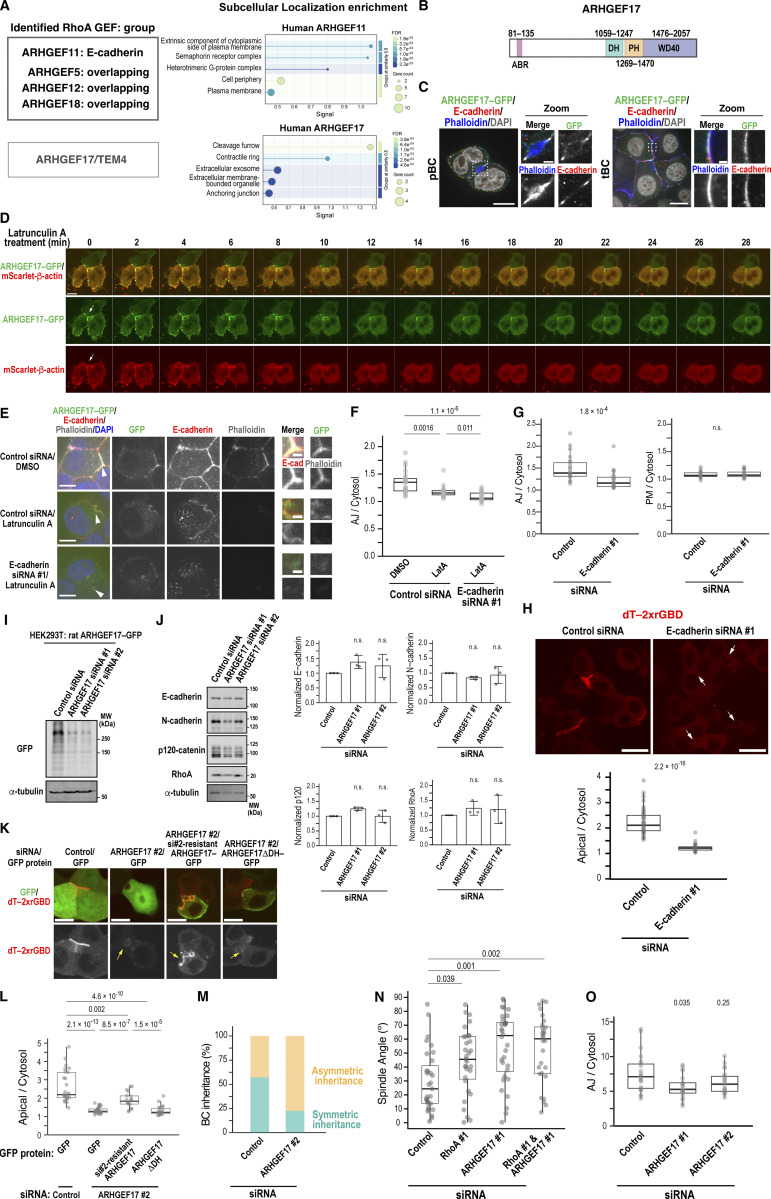
**Localization and function of ARHGEF17 in relation to E-cadherin, roles of E-cadherin and ARHGEF17 in RhoA activation, and roles of RhoA and ARHGEF17 in spindle orientation, related to Fig. 7. (A)** RhoA-specific GEFs identified by BioID analysis. ARHGEF17 is shown as an isolated candidate because it was detected in both E-cadherin replicates without a background signal in biotin (−) but was excluded by the filter due to high background intensity in untransfected cells. GO enrichment analysis of the protein–protein interaction network (STRING) is shown on the right. Note that ARHGEF17 is predicted to localize to the cleavage furrow and contractile ring, whereas ARHGEF11 is not. **(B)** Schematic of rat ARHGEF17. ABR, actin-binding region; DH, Dbl homology; PH, pleckstrin homology; WD40, WD40-repeat domain. **(C)** ARHGEF17 localizes to the cell cortex and AJs in cells with primordial or tubular BCs. Shown are representative confocal images of Can 10 cells expressing ARHGEF17-GFP, cultured for 2 days after transduction, and stained with DAPI, phalloidin, and anti-E-cadherin antibody. **(D–F)** Localization of ARHGEF17 at AJs depends on F-actin and E-cadherin. **(D)** Time-lapse montage of Can 10 cells expressing ARHGEF17-GFP and mScarlet–β-actin before and after treatment with LatA. Arrows indicate AJs positive for ARHGEF17. See also Video 2. **(E)** Representative confocal images of Can 10 cells expressing ARHGEF17-GFP after treatment with 10 µM LatA for 30 min, combined with control siRNA or E-cadherin siRNA, and stained with DAPI, phalloidin, and anti-E-cadherin antibody. The regions indicated by arrowheads are shown as zoomed images. **(F)** Quantification of ARHGEF17-GFP intensity at AJs relative to the cytoplasm following treatment with LatA and/or siRNA. Data are from three independent experiments (≥16 cells per condition). **(G)** E-cadherin is required for ARHGEF17 localization at AJs but not PM. Quantification of ARHGEF17-GFP intensity at AJs relative to the cytoplasm (left) or PM to the cytoplasm (right) in control siRNA- or E-cadherin siRNA #1–transfected cells. Data are from three independent experiments (≥22 cells per condition). **(H)** Decrease in RhoA activity upon E-cadherin depletion. Top: representative confocal images of dT-2×rGBD–expressing Can 10 cells transfected with E-cadherin siRNA. Arrows indicate BCs with the reduced intensity of active RhoA in E-cadherin knockdown cells. Bottom: ratios of dT-2×rGBD intensities at the BC vs. cytoplasm in control and E-cadherin-knockdown cells. Values are from two independent experiments (≥47 cells per condition). **(I)** Validation of ARHGEF17 siRNA efficiency. Immunoblot analysis of ARHGEF17-GFP-expressing HEK293T cells transfected with rat ARHGEF17 siRNAs. MWs of marker proteins are indicated in kDa. **(J)** ARHGEF17 depletion does not affect protein levels of E-cadherin, N-cadherin, p120-catenin, and RhoA. Left: representative immunoblots from Can 10 cells transfected with ARHGEF17 siRNAs. Right: quantification of indicated protein levels normalized to α-tubulin. Data represent means ± SD from three independent experiments; values are normalized to control siRNA-transfected cells. **(K and L)** Restoration of Rho activity by rescue expression of ARHGEF17. **(K)** dT-2×rGBD–expressing Can 10 cells were transfected with ARHGEF17 siRNA #2 one day earlier, after which they were transduced with lentiviruses expressing either GFP, si#2-resistant ARHGEF17-GFP, or ARHGEF17ΔDH-GFP. Representative confocal images are shown. Yellow arrows indicate BCs in ARHGEF17-knockdown cells. Note that RhoA activity was only restored by the expression of siRNA-resistant ARHGEF17. **(L)** Ratios of dT-2×rGBD intensities at the BC vs. cytoplasm in the cells shown in (K). Values are from two independent experiments (≥18 cells per condition). **(M)** ARHGEF17 regulates oriented cell division. Proportions of dividing cells expressing dT-2×rGBD that undergo symmetric vs. asymmetric BC inheritance were quantified following transfection with control siRNA or ARHGEF17 siRNA and time-lapse confocal imaging from two independent experiments (≥31 cells per condition). **(N)** Depletion of ARHGEF17 and RhoA alters mitotic spindle orientation. Combined scatter and box-and-whisker plots show spindle angle distributions relative to the BC. Data are from two independent experiments (≥28 cells per condition). **(O)** Quantitative analysis of E-cadherin localization at AJs. Ratios of E-cadherin intensity at AJs to cytoplasmic intensity are shown. Data are from two independent experiments (≥21 cells per condition). Scale bars, 1 µm (zoomed images in E), 5 µm (E), 10 µm (D and K), 20 µm (C and H). P values are indicated at the top of each graph; n.s., not significant. MWs, molecular weights. Source data are available for this figure: SourceData FS5.

**Figure 7.**
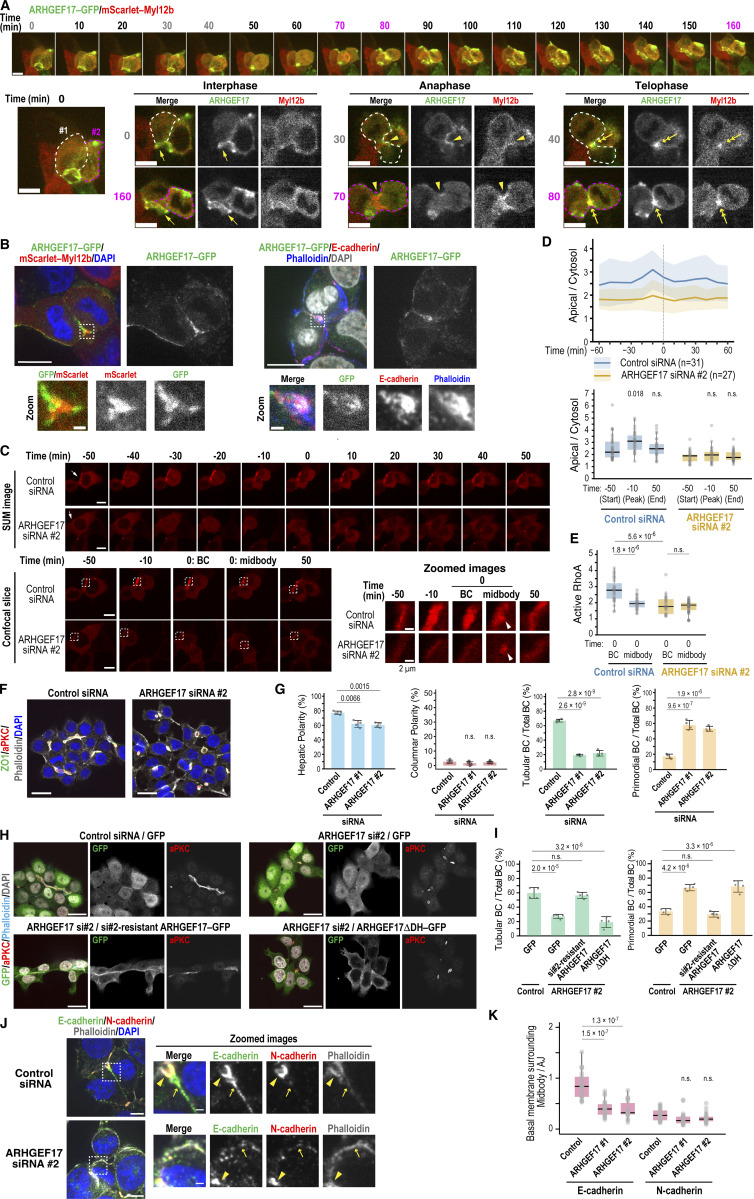
**ARHGEF17 activates RhoA at the apical region and cleavage furrow to promote BC elongation. (A)** Localization of ARHGEF17 during interphase and cytokinesis. Montage of time-lapse imaging of Can 10 cells expressing ARHGEF17-GFP and mScarlet–Myl12b during cytokinesis. A maximum-intensity projection of cells #1 and #2, outlined in white and magenta, respectively, at time 0 is shown in the bottom left corner. Confocal slice images of interphase (#1, 0 min; & #2, 160 min), anaphase (#1, 30 min; & #2, 70 min), and telophase (#1, 40 min; & #2, 80 min) are shown at the bottom. Yellow arrows, arrowheads, and double arrows indicate BCs, the cleavage furrow, and the midbody, respectively. **(B)** ARHGEF17 localizes to the daughter cell interface near the midbody during late cytokinesis. Shown are representative confocal images of Can 10 cells expressing ARHGEF17-GFP alone (right) or with mScarlet–Myl12b (left) cultured for 2 days after transduction, and stained as indicated. **(C)** ARHGEF17 is required for RhoA activation at the apical region and cleavage furrow during cytokinesis. Time-lapse montage of Can 10 cells transfected with control or ARHGEF17 siRNAs and expressing dT-2×rGBD. SUM projections (top), confocal images (bottom), and zoomed views of boxed regions are shown. Arrows (−50 min) indicate BCs; arrowheads (0 min) indicate the midbody. See also Video 3. **(D)** Quantitative analysis of RhoA activation in cells with a preexisting BC. Ratios of dT-2×rGBD intensities at the BC vs. cytoplasm in control and ARHGEF17-knockdown cells imaged in C are plotted over time (top), and at selected time points (bottom). Bold lines and shaded bands represent the mean and SD, respectively, from two independent experiments (*n* = 31 cells transfected with control siRNA; *n* = 27 cells transfected with ARHGEF17 siRNA). **(E)** ARHGEF17 primarily activates RhoA at the apical region, but not at the midbody. Ratios of dT-2×rGBD intensity at the BC or midbody to cytoplasm are shown in cells from C. Data represent combined scatter and box-and-whisker plots of active RhoA in control cells (*n* = 31) and ARHGEF17-knockdown cells (*n* = 27) from two independent experiments. **(F)** ARHGEF17 is required for BC elongation. Shown are representative confocal images of Can 10 cells transfected with control siRNA or ARHGEF17 siRNA, cultured for 72 h, and stained with DAPI, phalloidin, and antibodies against aPKC and ZO-1. **(G)** Quantification of polarity and BC structures in control and ARHGEF17-knockdown Can 10 cells. Data represent means ± SD from four independent experiments (≥373 cells per condition). **(H and I)** GEF activity of ARHGEF17 is required for BC elongation. Can 10 cells were transfected with ARHGEF17 siRNA #2 one day earlier, after which they were transduced with lentiviruses expressing either GFP, si#2-resistant ARHGEF17-GFP, or ARHGEF17ΔDH-GFP. Cells were fixed and stained using antibodies against GFP and aPKC, along with DAPI and phalloidin. Representative confocal images are shown. **(I)** Quantification of BC structures in cells shown in H. Data represent means ± SD from four independent experiments (≥44 cells per condition). **(J and K)** ARHGEF17 is required for E-cadherin accumulation at the cleavage furrow adjacent to the midbody. Confocal images of control and ARHGEF17-knockdown cells stained with DAPI, phalloidin, and antibodies against E- and N-cadherin. Arrowheads and arrows indicate AJs and the midbody region, respectively. **(K)** Quantitative analysis of E-cadherin and N-cadherin distribution at the division site. Ratios of cadherin intensity at the basal side of the midbody to their intensity at AJs are shown in cells from J. Data are from two independent experiments (≥21 cells per condition). See also Fig. S5 O. Scale bars, 1 µm (zoomed images in B and J), 2 µm (zoomed images in C), 10 µm (A, B, C, and J), 20 µm (F and H). P values are shown above each graph; n.s., not significant.

Since RhoA is activated at the apical region (presumably at the AJ) and the cleavage furrow of dividing cells during BC elongation, we aimed to identify the guanine nucleotide exchange factors (GEFs) responsible for these activations. Among the RhoA-specific GEFs captured by our BioID assay, ARHGEF17/TEM4 is the only one reported to function in both AJ assembly (Ngok et al., 2013) and cytokinesis (Prifti et al., 2025), although its localization at the cleavage furrow has not been described, it is expected to localize there (Fig. S5, A and B). Thus, we first examined its localization in Can 10 cells. ARHGEF17–GFP colocalized with E-cadherin around BCs (Fig. S5 C). This localization was reduced following treatment with latrunculin A (LatA), an F-actin depolymerization agent (Fig. S5, D and E; and Video 2) (Spector et al., 1989; Yarmola et al., 2000). Combined treatment with LatA and E-cadherin knockdown abolished this localization (Fig. S5, E and F), suggesting that ARHGEF17 requires both F-actin and E-cadherin for its recruitment to AJs. Notably, E-cadherin was required for AJ localization of the GEF, but not for its PM localization (Fig. S5 G). This is consistent with a previous report that ARHGEF17 localizes to cell–cell contacts in a cadherin–catenin complex-dependent manner (Ngok et al., 2013). Supporting this observation, Rho activity around the BC was markedly reduced by E-cadherin depletion (Fig. S5 H). To examine the dynamics of ARHGEF17 during cytokinesis, we tracked ARHGEF17–GFP with the mScarlet-tagged cytokinesis marker Myl12b, an isoform of myosin regulatory light chains (Brito and Sousa, 2020). Live-cell imaging revealed that ARHGEF17 localized to the cleavage furrow and then concentrated around the midbody (Fig. 7 A, Cell #1, time points 30 and 40; Cell #2, time points 70 and 80). Interestingly, when the long axis of a dividing cell was aligned parallel to a preexisting BC, its midbody formed near the BC (Fig. 7 A, Cell #1). In contrast, when the long axis of a dividing cell was oblique to the BC, its midbody initially formed away from the BC but was eventually pulled and incorporated into the preexisting BC (Fig. 7 A, Cell #2). Higher magnification image analysis showed that ARHGEF17 was distributed at the daughter cell interface around the midbody, where E-cadherin was also observed (Fig. 7 B). These findings suggest that ARHGEF17 plays a role in coordinating cytokinesis with new cell–cell contact formation.

**Video 2.** **Time-lapse analysis of ARHGEF17-GFP and mScarlet–β-actin localization before and after LatA treatment in Can 10 cells (Fig. S5 D).** Can 10 cells expressing ARHGEF17-GFP (green) and mScarlet–β-actin (red) were treated with 10 µM LatA at time 00:00 and imaged by spinning disk confocal microscopy. The video shows the effect of F-actin disruption on ARHGEF17 localization at AJs and the cell cortex. Images were acquired at 2-min intervals. Time is displayed as hh:mm, and the movie is shown at 2 frames per sec. Related to Fig. S5 D.

To assess whether ARHGEF17 activates RhoA during cytokinesis-linked BC elongation, we analyzed dT-2×rGBD–expressing cells with or without ARHGEF17 knockdown (Fig. 7 C; Fig. S5, I and J ; and Video 3). In contrast to control cells, which showed high RhoA activity around the BC and its elevation during cytokinesis, ARHGEF17-knockdown cells exhibited decreased basal RhoA activity at the BC (Fig. 7, C and D, time point −50) and failed to increase RhoA activity during cytokinesis (Fig. 7, C and D). This is due to DH domain–dependent Rho activation, since the full-length, siRNA-resistant ARHGEF17 restored the apical Rho activity, whereas the DH-deletion mutant did not (Fig. S5, K and L). Furthermore, ARHGEF17 depletion led to increased asymmetric BC inheritance (Fig. S5 M) and randomized spindle angles (Fig. S5 N), indicating a role in the regulation of spindle orientation. However, RhoA intensity at the midbody was not significantly affected in the ARHGEF17-knockdown cells, suggesting that other GEF(s) may be responsible for activating RhoA at this site (Fig. 7 E). These results indicate that ARHGEF17 is essential for RhoA activation around BCs, especially during cytokinesis, linking cytokinesis to BC development. In support of this, ARHGEF17 knockdown impaired tubular BC formation (Fig. 7, F and G) but increased primordial BCs, phenocopying RhoA knockdown and ROCK inhibition (Fig. 5 F and Fig. 6 C). The tubular architecture of BCs was restored by the expression of full-length, siRNA-resistant ARHGEF17, but not by its ΔDH variant, confirming that the GEF activity of ARHGEF17 is required for BC elongation (Fig. 7, H and I). We next examined whether ARHGEF17 contributes to cadherin localization at later stages of cytokinesis and found that ARHGEF17 depletion impaired E-cadherin recruitment to the daughter cell interface during telophase (Fig. 7, J and K). Conversely, ARHGEF17 knockdown did not markedly alter E-cadherin localization at AJs (Fig. S5 O). Thus, ARHGEF17 is required for RhoA activation around the BCs and E-cadherin accumulation at nascent cell–cell contact sites, thereby promoting cytokinesis-linked BC elongation.

**Video 3.** **Time-lapse analysis of active RhoA dynamics in control and ARHGEF17-depleted Can 10 cells (Fig. 7 C).** Can 10 cells expressing the active RhoA biosensor (dT-2×rGBD) and containing a preexisting BC were transfected with control siRNA (left) or ARHGEF17 siRNA (right). At 48 h after transfection, cells were imaged by spinning disk confocal microscopy. The video shows that ARHGEF17 is required for RhoA activation at the BC but not at the midbody. Red fluorescence indicates dT-2×rGBD. Images were acquired at 10-min intervals. Time is displayed as hh:mm, and the movie is shown at 2 frames per sec. Related to Fig. 7 C.

### p190B maintains hepatic polarity by inhibiting RhoA activity

**Figure 8.**
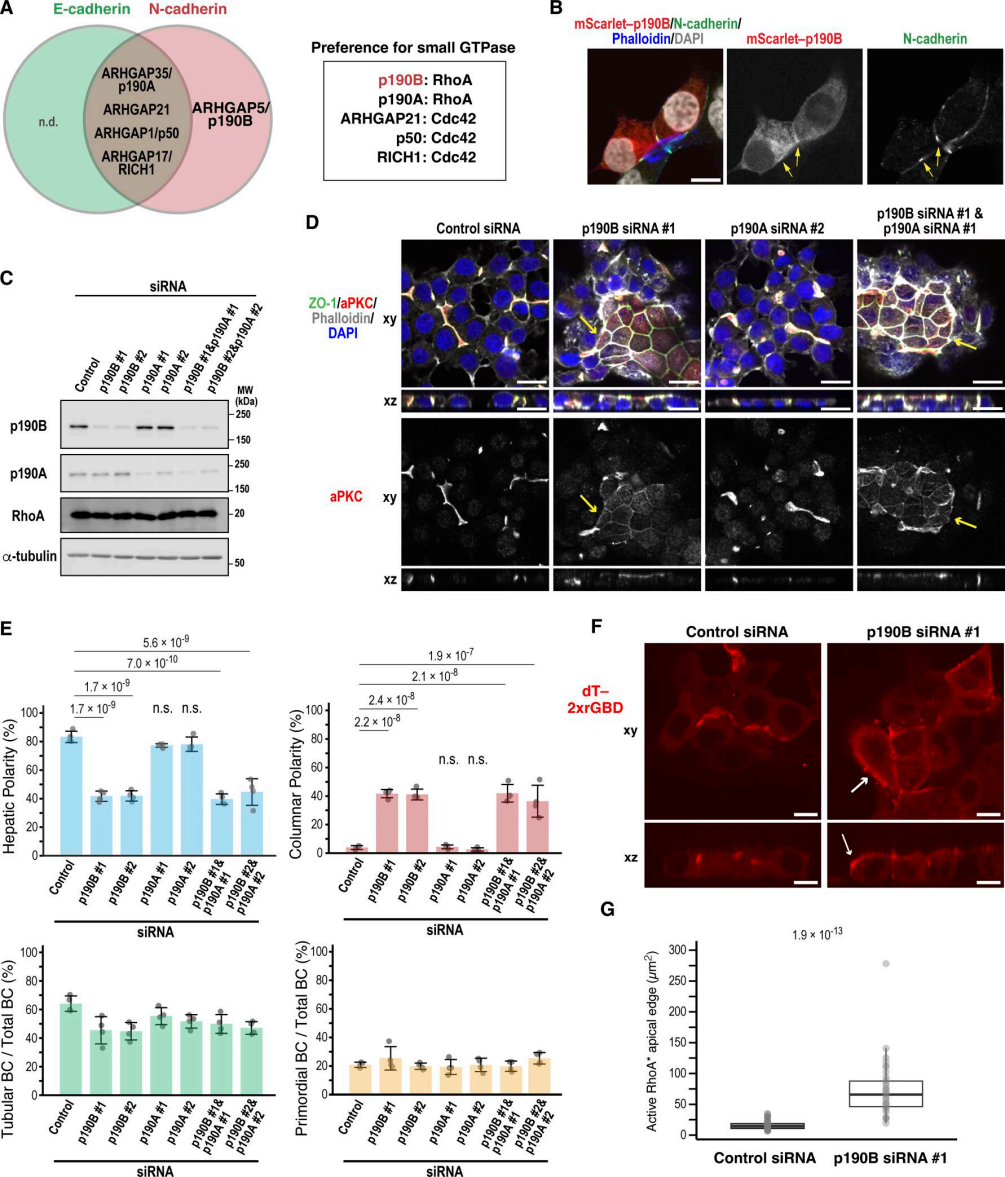
**p190B maintains hepatic polarity by downregulating RhoA activity. (A)** Venn diagram of ARHGAP proteins identified by BioID analysis. Their preferences for Rho family small GTPases are shown on the right. The RhoGAP specific to N-cadherin is indicated in red. Note that the E-cadherin–specific group does not include any ARHGAPs. n.d., not detected. **(B)** Localization of p190B in Can 10 cells. Representative confocal images of Can 10 cells expressing mScarlet–p190B are shown. Cells were cultured for 3 days, fixed, and stained with DAPI, phalloidin, and an anti-N-cadherin antibody. Yellow arrows indicate accumulation of p190B at N-cadherin–positive AJs. **(C)** Depletion of p190A and/or p190B does not alter RhoA levels. Immunoblot analysis of Can 10 cells transfected with p190A and/or p190B siRNAs. MWs of marker proteins are indicated in kDa. **(D)** p190B, but not p190A, is required for hepatic polarity maintenance. Shown are representative confocal images of Can 10 cells transfected with p190A and/or p190B siRNAs, cultured for 72 h, and stained with DAPI and antibodies against ZO-1 and aPKC. Yellow arrows indicate the apical membrane in columnar polarity cells. **(E)** Quantification of polarity and BC structures in cells from D. Data represent means ± SD from four independent experiments (≥431 cells per condition). **(F and G)** Elevated RhoA activity induced by p190B depletion. **(F)** Representative confocal images of Can 10 cells transfected with p190B siRNA and expressing dT-2×rGBD. Arrows indicate high RhoA activity at the apical edge in columnar polarity cells. **(G)** Product of the apical edge surface area with high probe activity and the mean intensity ratio of dT-2×rGBD at BCs versus cytoplasm in control or p190B-knockdown cells. Values are from two independent experiments (≥34 cells per condition). Values between the two groups were compared using the Wilcoxon rank-sum test. Scale bars, 10 µm (B and F), 20 µm (D). P values are indicated at the top of each graph; n.s., not significant. MWs, molecular weights. Source data are available for this figure: SourceData F8.

We next sought to understand how N-cadherin deficiency induces columnar polarity. Previous studies suggest that elevated RhoA activity alters polarity orientation in 3D-cultured epithelial cells (Yu et al., 2008) and is associated with columnar but not hepatic polarity (Lazaro-Dieguez and Musch, 2017). These observations raise the possibility that N-cadherin maintains hepatic polarity by inactivating RhoA at AJs following its activation by E-cadherin and ARHGEF17. To explore this, we searched our BioID dataset for Rho GTPase–activating proteins (RhoGAPs). Notably, only two RhoA-specific GAPs were identified: ARHGAP5 (p190B) and ARHGAP35 (p190A), with p190B specifically found as an N-cadherin–proximal protein (Fig. 8 A and Table S3). p190B was not only mainly distributed in the cytoplasm but also localized to N-cadherin–positive AJs in Can 10 cells (Fig. 8 B). Consistent with the BioID data, p190B knockdown caused a significant shift from hepatic to columnar polarity, whereas p190A depletion had no effect (Fig. 8, C–E). Double knockdown of both p190A and p190B did not further enhance columnar polarity compared with p190B single depletion alone, suggesting no redundant roles in this process (Fig. 8, C–E). To determine whether the polarity switch induced by p190B depletion was due to increased RhoA activity, we measured the apical intensity of dT-2×rGBD in control and p190B-knockdown cells. Notably, p190B depletion significantly expanded the area of high RhoA activity along the apical edge (Fig. 8, F and G). Taken together, these findings indicate that p190B-mediated inactivation of RhoA is essential for maintaining hepatic polarity.

### ARVCF and p120-catenin maintain hepatic polarity downstream of N-cadherin

**Figure 9.**
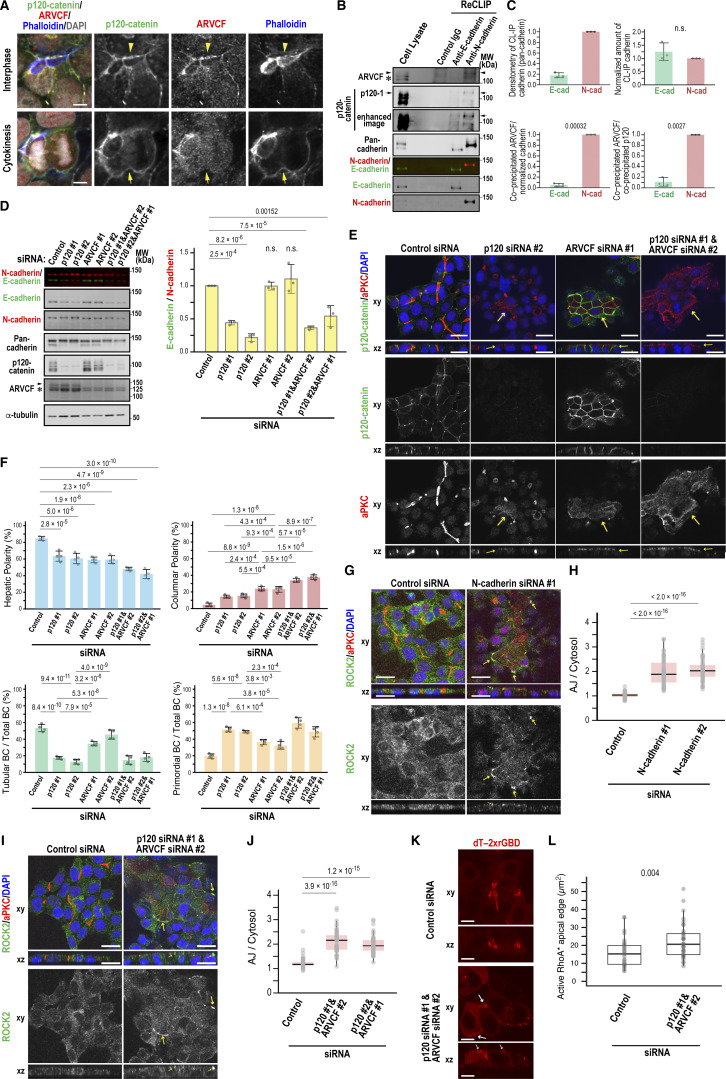
**ARVCF mediates the role of N-cadherin in hepatic polarity maintenance by downregulating RhoA activity. (A)** Localization of p120-catenin and ARVCF during interphase and cytokinesis. Shown are representative iSIM images of Can 10 cells cultured for 3 days and stained with DAPI, phalloidin, and antibodies against p120-catenin and ARVCF. Yellow arrowheads and arrows indicate AJs and the cleavage furrow, respectively. **(B)** N-cadherin, but not E-cadherin, preferentially associates with ARVCF over p120-catenin. Lysates of Can 10 cells treated with the reversible cross-linker DSP were immunoprecipitated using control IgG, anti-E-cadherin, or anti-N-cadherin antibody, followed by SDS-PAGE and immunoblot analysis. The asterisk indicates nonspecific bands in the lysates detected by the anti-ARVCF antibody. MWs of marker proteins are indicated in kDa. **(C)** Quantification of ARVCF and p120-catenin binding preference for E- and N-cadherin. Top: intensities of pulled-down cadherins (left) and their normalized protein amounts (right). Bottom: ratios of coprecipitated ARVCF (left) or ARVCF/p120-catenin (right) normalized to the cadherin pulldown. Data represent means ± SD from three independent experiments. The ARVCF/N-cadherin ratio (left) and the ARVCF/p120-catenin ratio in N-cadherin pulldowns (right) are set to 1.0. See also Fig. S1 H. **(D)** Knockdown of p120, but not ARVCF, destabilizes E-cadherin without affecting N-cadherin. Left: representative immunoblots of single and double knockdown cells. The asterisk indicates nonspecific bands in the lysates detected by the anti-ARVCF antibody. Right: quantification of E-cadherin-to-N-cadherin ratios. Data represent means ± SD from three independent experiments; the ratio in control siRNA-transfected cells is set to 1.0. **(E)** ARVCF primarily regulates hepatic polarity, while p120-catenin affects BC elongation. Shown are representative confocal images of Can 10 cells transfected with ARVCF and/or p120-catenin siRNAs, cultured for 72 h, and stained with DAPI and antibodies against p120-catenin and aPKC. Yellow arrows indicate the apical membrane in columnar polarity cells. **(F)** Quantification of polarity and BC structures in cells from E. Data represent means ± SD from four independent experiments (≥803 cells per condition). **(G and H)** N-cadherin depletion increases ROCK2 accumulation at the apical edge. **(G)** Representative confocal images of Can 10 cells transfected with control or N-cadherin siRNAs, cultured for 72 h, and stained with DAPI and antibodies against aPKC and ROCK2. Arrows indicate ROCK2 accumulation on the apical-most junctions. **(H)** Quantification of ROCK2 intensity at the apical edge vs. cytoplasm. Data are from two independent experiments (≥54 cells per condition). **(I and J)** ROCK2 accumulates at AJs in ARVCF/p120-catenin double knockdown cells. Shown are representative confocal images of Can 10 cells transfected with both siRNAs, cultured for 72 h, and stained with DAPI and antibodies against aPKC and ROCK2. Arrows indicate ROCK2 accumulation on the apical-most junctions. **(J)** Quantification of ROCK2 localization at AJs. Ratios of ROCK2 intensity at the apical edge vs. cytoplasm are shown for control and double knockdown cells. Data are from two independent experiments (≥51 cells per condition). **(K and L)** Elevated Rho activity induced by double depletion of ARVCF/p120-catenin. **(K)** Representative confocal images of Can 10 cells transfected with both siRNAs and expressing dT-2×rGBD. Arrows indicate high RhoA activity at the apical edge in columnar polarity cells. **(L)** Product of the apical edge surface area with high probe activity and the mean intensity ratio of dT-2×rGBD at BCs versus cytoplasm in control or the double knockdown cells. Values are from two independent experiments (≥32 cells per condition). Values between the two groups were compared using the Wilcoxon rank-sum test. Scale bars, 5 µm (A), 10 µm (K), 20 µm (E, G, and I). P values are indicated at the top of each graph; n.s., not significant. MWs, molecular weights. Source data are available for this figure: SourceData F9.

Since p190B interacts with p120-catenin and its paralog ARVCF (Cho et al., 2010; Ponik et al., 2013; Wildenberg et al., 2006), we investigated the localization and function of these catenins in Can 10 cells. While p120-catenin and ARVCF colocalized at AJs during interphase (Fig. 9 A), they exhibited distinct localizations during cytokinesis: p120-catenin was present at the PM, including the cleavage furrow, whereas ARVCF was restricted to AJs surrounding BCs (Fig. 9 A, cytokinesis), reminiscent of the differential localization of E-cadherin and N-cadherin (Fig. 3 J). Consistent with this, ReCLIP assays using DSP demonstrated that endogenous ARVCF was coprecipitated more efficiently with N-cadherin than with E-cadherin (Fig. 9, B and C; and Fig. S1, F and G). ARVCF also showed a preference for N-cadherin over p120-catenin (Fig. 9 C). The association between p120-catenin and E-cadherin was confirmed by the observation that only E-cadherin was markedly reduced in p120-catenin–knockdown cells, whereas ARVCF depletion did not affect cadherin stability (Fig. 9 D). These results suggest that p120-catenin and ARVCF preferentially interact with E-cadherin and N-cadherin, respectively, with p120-catenin specifically stabilizing E-cadherin.

To assess their roles in hepatic polarity and BC development, we examined single and double knockdowns of p120-catenin and ARVCF (Fig. 9 D). p120-catenin knockdown decreased tubular BCs but increased primordial BCs (Fig. 9, E and F), similar to E-cadherin depletion. In contrast, ARVCF knockdown had a mild effect on tubular BC formation and mainly increased columnar polarity (Fig. 9, E and F). This columnar polarity was further enhanced by additional depletion of p120-catenin (Fig. 9, E and F). These results suggest that p120-catenin primarily functions with E-cadherin in BC elongation, while ARVCF, with minor input from p120-catenin, functions with N-cadherin to maintain hepatic polarity.

We next examined whether RhoA signaling was activated in columnar cells induced by N-cadherin depletion or by double knockdown of p120-catenin and ARVCF. In control cells, ROCK2 was predominantly cytoplasmic and barely detectable at the PM, whereas in N-cadherin–depleted cells exhibiting columnar polarity, ROCK2 concentrated at the apical region of cell–cell contacts (Fig. 9, G and H). A similar pattern was observed in columnar cells induced by combined knockdown of p120-catenin and ARVCF (Fig. 9, I and J). These findings suggest that aberrant activation of RhoA-ROCK signaling occurs upon N-cadherin loss or double depletion of p120-catenin and ARVCF, likely due to the absence and/or inactivation of p190B at AJs. Consistent with this idea, double knockdown of p120-catenin and ARVCF expanded the area of high Rho activity along the apical edge (Fig. 9, K and L). Together, these observations suggest that ARVCF and its binding partner p190B mediate the role of N-cadherin in maintaining hepatic polarity by inactivating RhoA at AJs.

## Discussion

In this study, we reveal that hepatocytes employ E- and N-cadherin to regulate hepatic polarity and drive BC biogenesis through spatiotemporal control of RhoA activity in an opposing manner, addressing a fundamental question in liver biology. While both cadherins cooperate in polarity establishment, they also serve distinct, nonredundant roles. E-cadherin drives cytokinesis-linked BC elongation by coordinating spindle orientation and RhoA activation through NuMA and the RhoGEF ARHGEF17, which also facilitates its recruitment to daughter cell interfaces for nascent cell–cell contact formation. In contrast, N-cadherin maintains hepatic polarity by promoting RhoA inactivation via ARVCF and the RhoGAP p190B. Collectively, these findings define the shared and unique roles of E- and N-cadherin in hepatic polarity and BC formation, processes essential for liver architecture and function.

### Co-expression of E- and N-cadherin is required for the establishment and maintenance of hepatic polarity

E-cadherin is predominantly expressed in epithelial tissues, where it mediates AJ assembly and function, particularly in columnar or cuboidal epithelial cells (Halbleib and Nelson, 2006; Wu and Yap, 2013; Zhang et al., 2023). In contrast, N-cadherin is mainly expressed in nonepithelial tissues such as neurons and cardiomyocytes, where it mediates the formation of synaptic junctions and intercalated disks, thereby supporting synaptic transmission and mechanical force transduction, respectively (Laszlo and Lele, 2022; Vite and Radice, 2014). A switch from E-cadherin to N-cadherin expression is commonly observed during EMT in development and in cancer metastasis (Loh et al., 2019; Mrozik et al., 2018; van Roy, 2014; Yu et al., 2019). However, unlike these situations where E- and N-cadherin are largely expressed in a mutually exclusive manner, embryonic hepatoblasts and hepatocytes express both E- and N-cadherin simultaneously during mouse liver development, which becomes zonally restricted after birth (Doi et al., 2007) (Fig. 1, C and D; and Fig. S1 E). The reason for this expression pattern in hepatoblasts and hepatocytes remains completely unknown. Our analyses in this study provide insight into this question.

Unlike other simple epithelia, where a layer of columnar or cuboidal epithelial cells surrounds a central apical lumen, hepatoblasts and hepatocytes form a tiny apical lumen—the BC—between neighboring cells (Decaens et al., 2008; Treyer and Musch, 2013). This process is spatially linked to cytokinesis, at least in Can 10 cells (Wang et al., 2014). In addition, BC elongation is associated with oriented cell division (Wang et al., 2014). To explore whether E- and N-cadherin co-expression contributes to this unique hepatic polarity, we performed localization and knockdown studies of E- and/or N-cadherin in Can 10 cells. We found that both E-cadherin and N-cadherin localized to AJs, but E-cadherin uniquely showed additional localization at the cleavage furrow and nascent cell–cell contact sites between daughter cells. Strikingly, E-cadherin knockdown prevented cell division–linked BC elongation, whereas N-cadherin knockdown caused a switch from hepatic polarity to columnar polarity. Double knockdown significantly reduced the number of cells displaying the basic apical–basal polarity. These analyses indicate that both cadherins act in concert to establish and maintain hepatic polarity, while E-cadherin is specialized in BC elongation and N-cadherin in polarity maintenance.

The in vivo roles of E- and N-cadherin remain unclear. However, the co-expression of both cadherins occurs only during liver development (Doi et al., 2007). A liver-specific knockout of E-cadherin, but not N-cadherin, was generated in mice using the *Alfp-Cre* driver (Battle et al., 2006). This knockout did not appear to affect tight junction assembly at E18.5, and its effects on AJ assembly and BC formation remain to be fully characterized. Given the vital role of E-cadherin in cell division–dependent BC elongation observed in vitro (Wang et al., 2014) (this study), it is reasonable to speculate that E-cadherin plays a major role in BC elongation during early stages of liver development, when cell proliferation is high, rather than during late stages when proliferation declines and BCs begin to form a network that may involve cell division–independent mechanisms. In this context, the expression levels of E-cadherin during early stages (E11.5 to E17.5) of liver development in liver-specific knockout mice should be quantitatively assessed in conjunction with examination of BC architecture.

Strikingly, cadherin distribution becomes zonally restricted after birth: E-cadherin is predominantly expressed in the periportal vein region (Zone 1) of the liver lobule, whereas N-cadherin shows a complementary pattern with highest expression in the pericentral vein region (Zone 3) (Doi et al., 2007). During regeneration, hepatocytes, especially those from the intermediate region (Zone 2), can upregulate E-cadherin, presumably resulting in co-expression of both cadherins (He et al., 2021; Lin et al., 2023; Wang et al., 2024). These findings suggest that E- and N-cadherin co-expression is likely linked to cell division–dependent hepatoblast and hepatocyte polarization and BC biogenesis, a possibility supported by our in vitro observations. They also indicate that Can 10 cells resemble developing and regenerating hepatocytes, rather than adult hepatocytes.

### Opposing RhoA regulation by E- and N-cadherin controls BC elongation and hepatic polarity

**Figure 10.**
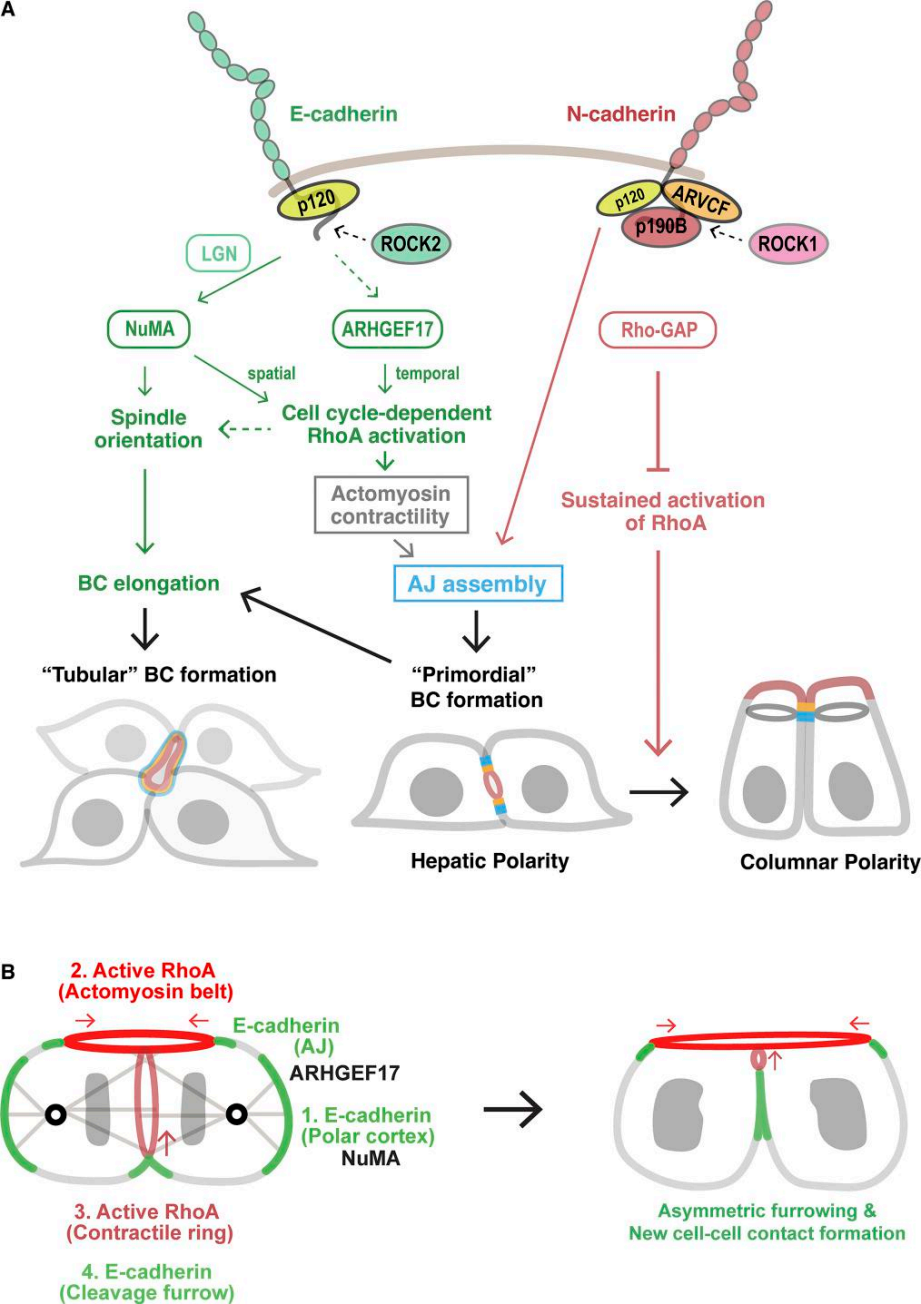
**Models for cytokinesis-linked hepatocyte polarization and BC formation. (A)** Schematic illustrating the overlapping and distinct roles of E-cadherin and N-cadherin in controlling BC elongation and maintaining hepatic polarity via opposing effects on RhoA activity. E-cadherin activates RhoA at the AJs surrounding the BC and at the cleavage furrow via ARHGEF17, while restricting active RhoA to the division site via NuMA at the lateral membrane during cytokinesis. This spatially restricted RhoA activity enables the recruitment of E-cadherin to nascent cell–cell contacts between daughter cells, promoting new AJ assembly during primordial BC formation and BC elongation. ARHGEF17 and NuMA also contribute to BC elongation by regulating spindle orientation (see below). While N-cadherin shares the role of promoting AJ assembly during primordial BC formation with E-cadherin, it also decreases RhoA activity at AJs via ARVCF and p190B RhoGAP, thereby preventing an unwanted switch from hepatic to columnar polarity. **(B)** Model depicting the roles of two distinct mechanical forces in regulating spindle orientation during BC elongation. The first force is generated by E-cadherin–NuMA–dynein–astral MT pathway at the cell poles (#1), which governs the initial selection of spindle orientation. The second force arises from a RhoA-mediated actomyosin belt at the AJs surrounding the BC membrane, activated by ARHGEF17 (#2). This force stabilizes spindle orientation by anchoring the dividing cell at the BC side of its cleavage furrow and also provides an asymmetric mechanical cue that biases furrow ingression toward the BC (#3 and red arrow). RhoA-ROCK-dependent roles during and after contractile ring constriction enable E-cadherin delivery and/or stabilization at the division site during cytokinesis, thereby promoting nascent AJ assembly between daughter cells and driving BC elongation (#4).

To determine how E- and N-cadherin act in concert to drive hepatic polarity and BC biogenesis at the mechanistic level, we identified their specific interactors using the BioID assay and found that they regulate these processes by exerting opposite control on RhoA activity (Fig. 10 A).

E-cadherin acts through its proximal binding partners, NuMA and ARHGEF17, to regulate spindle orientation and RhoA activation, thereby promoting cytokinesis-linked BC elongation. Time-lapse analysis revealed that among dividing cells with a preexisting BC, ∼66% display symmetric BC inheritance with elongation, with most dividing with a spindle parallel to the BC long axis. The remaining ∼34% display asymmetric inheritance without elongation, with most dividing with an oblique or perpendicular spindle relative to the BC (Fig. 4 C; and Fig. S4, A and B). Based on these observations and the temporal sequence of BC morphogenesis during liver development (Fig. 1 D and Fig. S1, A–D), we propose that spindle orientation is developmentally regulated, with more parallel divisions driving BC elongation during early development (E15.5–E17.5) and more oblique/perpendicular divisions promoting hepatocyte stratification and BC network formation during late development (P0, perinatal stage).

Our findings further suggest a model in which two mechanical forces coordinate to dictate spindle orientation in E-cadherin–mediated parallel divisions during BC elongation (Fig. 10 B). The first force acts at the cell poles, mediated by the E-cadherin–NuMA–dynein–astral microtubule (MT) pathway (di Pietro et al., 2016; Gloerich et al., 2017; Kiyomitsu and Boerner, 2021), and is responsible for the initial selection of spindle orientation (step 1). The second force acts at AJs surrounding the BC, generated by the ARHGEF17- and RhoA-dependent actomyosin belt, and stabilizes spindle orientation (step 2). This contractile activity also provides an asymmetric mechanical cue that biases furrow ingression toward the BC via the RhoA-activated cytokinetic actomyosin ring (step 3), a process common to epithelial planar divisions (Fleming et al., 2007; Jinguji and Ishikawa, 1992; Maddox et al., 2007; Thieleke-Matos et al., 2017; Wang et al., 2014). Following cytokinesis, E-cadherin delivery to the division site enables new AJ assembly between daughter cells (step 4).

This model predicts that disrupting either force—actomyosin at AJs or MT/dynein at the poles—impairs spindle orientation and thereby inhibits BC elongation. Consistent with this, depletion of E-cadherin, NuMA, ARHGEF17, or RhoA, or inhibition of ROCK, blocked BC elongation (Figs. 3, 4, 5, 6, and 7). Mechanistically, E-cadherin controls spindle orientation via at least two pathways (Fig. 10 B, steps 1 and 2): enhancing NuMA localization at the polar cortex during anaphase (Fig. 4, G and H; and Fig. S3 H), and activating RhoA at AJs likely via ARHGEF17 (Fig. 7, C–E; and Fig. S5, H and K–N). Notably, an AJ-associated spindle orientation mechanism (“rescue mechanism”) had been proposed in columnar epithelial cells such as MDCK cells but thought absent in hepatocytes (Lazaro-Dieguez and Musch, 2017). Our findings in Can 10 cells contrast this view, likely because Can 10 cells, like developing hepatocytes, form tubular BCs requiring planar divisions, whereas WIF-9B and HepG2 hepatocytes used previously cannot form tubular BCs and undergo nonplanar divisions. Furthermore, we identify ARHGEF17 as the RhoGEF recruited to AJs in an E-cadherin– and F-actin–dependent manner, where it activates RhoA to drive BC elongation. Together, these findings suggest that E-cadherin promotes BC elongation by coordinating spindle orientation and RhoA activation.

In contrast, N-cadherin localizes exclusively to linear AJs and inactivates RhoA via the p120-catenin isoform ARVCF and its partner p190B/ARHGAP5. Thus, E-cadherin activates RhoA through ARHGEF17 during division to promote BC elongation, whereas N-cadherin suppresses RhoA activity after cytokinesis in cells through p190B to prevent sustained ROCK activity, which would otherwise stabilize the actomyosin belt and enforce columnar polarity (Fig. 10 A) (Lazaro-Dieguez and Musch, 2017). We further find that the Rho effector ROCK1 localizes to AJs/BCs and primarily maintains hepatic polarity, placing it within the N-cadherin pathway (Fig. 6, E and F), whereas its paralog ROCK2 plays a more prominent role in BC elongation (Fig. 6, E and F), consistent with its identification as an E-cadherin–proximal interactor (Fig. 6 A). ROCK2 is predominantly cytoplasmic but is specifically recruited to the apical edge in cells exhibiting columnar polarity upon ROCK1 depletion (Fig. 6 G). Thus, ROCK1 normally functions at AJs/BCs to limit ROCK2 localization to these sites. When this antagonism is disrupted, such as upon ROCK1 depletion or aberrant RhoA activation (e.g., N-cadherin or p190B depletion), ROCK2 is ectopically recruited to AJs/BCs, promoting columnar polarity (Fig. 10 A).

Collectively, these findings suggest that E- and N-cadherin exert opposing, spatiotemporally coordinated control over RhoA activity, whereby E-cadherin promotes RhoA activation at AJs through ARHGEF17 to drive division-linked BC elongation, while N-cadherin attenuates RhoA activity via p190B-GAP to preserve hepatic polarity.

### Interplay of E-cadherin, RhoA, and NuMA during nascent contact formation between daughter cells

The establishment of nascent cell–cell contacts or the formation of nascent AJs between daughter cells remains poorly understood (Osswald and Morais-de-Sa, 2019). E-cadherin is delivered to the daughter cell interface at the terminal stage of cytokinesis during epithelial morphogenesis in *Drosophila* and *Xenopus* (Founounou et al., 2013; Herszterg et al., 2013; Higashi et al., 2016). In Can 10 cells, E-cadherin is present across the PM during cell division, including at AJs and the cleavage furrow, whereas N-cadherin predominantly accumulates in linear AJs (Fig. 3, J and K). These observations suggest that E-cadherin mediates nascent AJ assembly between daughter hepatocytes. This distribution is also consistent with distinct roles of E- and N-cadherin in the establishment and maintenance of hepatic polarity, respectively, despite their shared requirement for both processes.

How E-cadherin is delivered to and stabilized at the cleavage furrow or daughter cell interface remains unclear. We found that E-cadherin accumulation at these sites in Can 10 cells depends on ROCK (Fig. 6, J and K) and ARHGEF17 (Fig. 7, J and K), the effector and activator of RhoA, respectively. Both proteins localize to the cleavage furrow or daughter cell interface (Fig. S4 F; and Fig. 7, A and B), indicating that the ARHGEF17-RhoA-ROCK pathway is required for E-cadherin localization to the division site. It is possible that E-cadherin and its associated F-actin further recruit and/or stabilize ARHGEF17 at this site, as observed at AJs, thereby promoting local RhoA activation and establishing a positive feedback loop that enhances E-cadherin accumulation during nascent AJ assembly. Intriguingly, CG43102, the *Drosophila* ortholog of ARHGEF17, localizes to the cleavage furrow or daughter cell interface during and after cytokinesis in the notum (di Pietro et al., 2023). Whether this RhoGEF contributes to E-cadherin recruitment during nascent AJ assembly in *Drosophila* remains to be determined.

We uncovered a reciprocal dependency between E-cadherin and NuMA during division-linked BC elongation. During mitosis, particularly anaphase, E-cadherin is required for clustering NuMA at the polar cortex to regulate astral MT-mediated spindle orientation (Fig. 4, G and H). However, E-cadherin is not required for NuMA membrane association, as NuMA remains at the PM upon E-cadherin depletion, likely through LGN and Band 4.1 proteins (Kiyomitsu and Boerner, 2021; Lechler and Mapelli, 2021). LGN binds the juxtamembrane region of E-cadherin, which overlaps with the p120-catenin–binding site (Gloerich et al., 2017; Ishiyama et al., 2010), raising the possibility of competitive binding. During metaphase, E-cadherin localizes both to the polar cortex and to AJs surrounding preexisting BCs. In this context, p120-catenin is more enriched at AJs relative to the polar cortex than E-cadherin (median polar cortex-to-cytoplasm ratio: 0.335 for p120-catenin versus 0.587 for E-cadherin; *n* ≥ 21 cells; P = 1.4 × 10^−7^; Fig. 3 K). This differential distribution suggests that reduced p120-catenin at the polar cortex may permit LGN binding to E-cadherin, whereas its enrichment at AJs may limit LGN interaction, thereby preventing inappropriate spindle orientation.

In contrast, during cytokinesis, NuMA is required for E-cadherin accumulation at the division site (Fig. 4, I and J). Recent work in HeLa cells showed that NuMA restricts RhoA to the cleavage furrow, while RhoA activity, in turn, contributes to NuMA polarization (Sana et al., 2022). Consistent with this, we found that NuMA is required to confine RhoA to the basal side of the cleavage furrow in Can 10 cells (Fig. 6, H and I). These findings suggest that NuMA promotes E-cadherin accumulation by regulating the spatial distribution of RhoA at the PM. Although the underlying mechanism remains unclear, the marked reduction of RhoA at the PM in NuMA-depleted Can 10 cells raises the possibility that NuMA regulates RhoA activity through specific GEFs or GAPs, an area that warrants further investigation.

Collectively, these findings indicate that ARHGEF17 and NuMA mediate spatiotemporal control of RhoA activity at the division site to drive E-cadherin–dependent nascent AJ assembly between daughter cells (Fig. 10 A). In turn, efficient furrow ingression and nascent AJ assembly may reinforce spindle orientation, thereby promoting division-linked BC elongation (Fig. 10, A and B).

## Materials and methods

### Cell culture

Can 10 and Fao cells were grown in Ham’s F-12K (Kaighn’s) medium (21127022; Thermo Fisher Scientific) with 5% FBS (16000044; Gibco) and 0.2% Antibiotic–Antimycotic (15240062; Gibco). HEK293T cells were cultured in Dulbecco’s modified Eagle’s medium (11995065; Thermo Fisher Scientific) supplemented with 10% FBS (16000044; Gibco) and 1% Antibiotic–Antimycotic (15240062; Gibco).

### Mice

All experimental animal protocols were approved by the Institutional Animal Care and Use Committee of Peking University. The C57BL/6 mouse strain was used in this study. All mice were maintained under specific pathogen-free conditions at 23 ± 2°C with a 12-h day/night cycle. Female mice aged 8–12 wk were mated with adult male mice. The morning the vaginal plug was detected was designated E0.5.

### Mouse liver lobe culture

Mouse liver lobe culture was performed as previously described (Yang et al., 2017) with slight modifications. Liver tissues were cut into 1-mm^3^ cubes. Fetal liver explants were cultured for 2 days in the presence of DMSO (D2650; Sigma-Aldrich) or 1 µM Y27632 (S1049; Selleck).

### Antibodies and chemicals

Anti-E-cadherin (36/E-cadherin, 610182) and anti-N-cadherin (32/N-cadherin, 610921) monoclonal antibodies were purchased from BD Biosciences; anti-EpCAM-APC (G8.8, 17-5791-82) rat monoclonal, anti-P-Glycoprotein/mdr (C219, MA1-26528) and anti-ARHGAP5 (3L9I3, MA5-38043) mouse monoclonal, and anti-ZO-1 (40-2200; 61-7300), anti-ARVCF (PA5-64129), and anti-pan-cadherin (71-7100) rabbit polyclonal antibodies were from Thermo Fisher Scientific; anti-N-cadherin (13A9, 14215) and anti-β-catenin (L54E2, 2677) mouse monoclonal and anti-N-cadherin (D4R1H, 13116), anti-catenin δ-1/p120-catenin (D7S2M, 59854), anti-RhoA (67B9, 2117), anti-RhoB (D1J9V, 63876), anti-RhoC (D40E4, 3430), anti-ROCK1 (E2G4N, 28999), anti-ROCK2 (E5T5P, 47012), and anti-p190-A RhoGAP (C59F7, 2860) rabbit monoclonal antibodies were from Cell Signaling Technology; anti-NuMA1 (AD6-1, MABE1807) mouse monoclonal and anti-partitioning-defective 3 [Par3] (07-330) and anti-LGN (ABT174) rabbit polyclonal antibodies were from Millipore; anti-ZO-1 (R40.76, sc-33725) rat monoclonal and anti-p120 (15D2, sc-23872), anti-4.1R (B-11, sc-166759), anti-N-cadherin (13A9, sc-59987), anti-PKCzeta/aPKC (H-1, sc-17781), anti-MDR1 (D-11, sc-55510), and anti-RhoA (26C4, sc-418) mouse monoclonal antibodies were from Santa Cruz Biotechnology; anti-α-tubulin (66031-1-Ig) mouse monoclonal and anti-E-cadherin (20874-1-AP), anti-albumin (16475-1-AP), and anti-GFP (50430-2-AP) rabbit polyclonal antibodies were from Proteintech; anti-EPB41L2/4.1G (EPR8873(2), ab175928), and anti-pericentrin (EPR21987, ab220784) rabbit monoclonal antibodies were from Abcam; anti-radixin (GTX105408) and anti-E-cadherin (GTX100443) rabbit polyclonal antibodies were from GeneTex; goat anti-E-cadherin (AF748) polyclonal antibodies were from R&D Systems; rat anti-DLK-FITC (24-11, D187-4) monoclonal antibody was from MBL; and goat anti-HNF4A (LS-C758303) polyclonal antibodies were from LifeSpan BioSciences.

Y27632 (Y0503; Sigma-Aldrich) was used at a concentration of 3 µM for Can 10 cells. For liver explant culture, 1 µM Y27632 (S1049; Selleck) was used. DSP was purchased from Pierce (22585).

### Plasmid construction

The cDNAs encoding rat E-cadherin (aa 1–886), N-cadherin (aa 1–906), ARHGEF17 (aa 1–2057), β-actin (aa 1–375), Mzt1 (aa 1–78), radixin (aa 1–583), p190B (aa 1–1503), and Myl12b (aa 1–172) were obtained by RT-PCR using RNAs prepared from Can 10 cells. Mutations leading to the indicated amino acid substitutions or deletions were introduced by PCR-mediated site-directed mutagenesis. The cDNA for TurboID was a gift from Alice Ting (plasmid #107173; Addgene; RRID: Addgene_107173). For the expression of TurboID-tagged E-cadherin and N-cadherin, the cDNA encoding TurboID was inserted to the C terminus of the cadherins with a flexible linker 4×(GGGGS)GGGS. The cDNA for dT-2×rGBD was a gift from Dorus Gadella (plasmid #176098; Addgene; RRID: Addgene_176098). The obtained cDNAs were inserted into pLJM1 using In-Fusion Snap Assembly (638948; Takara Bio). All constructs were sequenced to confirm their identity. Plasmids used in this study are listed in Table S4.

### Lentivirus packaging and transduction in Fao and Can 10 cells

Lentivirus was packaged by cotransfection of the pLJM1 plasmids encoding EGFP, E-cadherin–EGFP, E-cadherin–mScarlet, E–cadherin–TurboID, N-cadherin–EGFP, N-cadherin–Turbo ID, ARHGEF17-EGFP, Mzt1–EGFP, radixin–mScarlet, mScarlet–p190B, mScarlet–β-actin, or Myl12b–mScarlet, with the packaging plasmids pMDLg/pRRE, pRSV-Rev, and the VSV-G envelope–expressing vector pMD2.G into HEK293T cells using Lipofectamine 3000 transfection reagent (L3000015; Thermo Fisher Scientific). The medium was changed to DMEM with 10% FBS 12 h after transfection. Lentivirus-containing supernatants were collected at 48 h after transfection. The collected supernatants were filtered through a Millex-HA syringe filter with a pore size of 0.45 µm (Millipore). For transduction of E-cadherin, N-cadherin, Myl12b, or 2×rGBD in Can 10 cells, the filtered lentivirus-containing medium was added to the cells in Ham’s F-12K medium with 5% FBS. For transduction of E-cadherin–mScarlet in Fao cells or Mzt1–GFP, radixin–mScarlet, ARHGEF17-EGFP in Can 10 cells, viral supernatants were concentrated using Lenti-X Concentrator (631231; Takara Bio) and added to the cells. The expression of the tagged proteins was assessed 96 h after transduction in Fao cells and 48 h after transduction in Can 10 cells.

### Generation of Can 10 cells stably expressing dT-2×rGBD

Lentivirus packaging and transduction of pLV-dT-2×rGBD were performed as described above. Puromycin was added to a final concentration of 2.5 µg/ml 48 h after infection for selection. After one week, single clones were isolated by plating TrypLE (Thermo Fisher Scientific)-dissociated cells at 1 cell/100 μl into 96-well plates, each well containing Ham’s F-12K medium with 5% FBS and 2.5 µg/ml puromycin. After 2 wk of culture, single clones were screened by microscopy for dT expression.

### siRNA-mediated knockdown and rescue experiment in Can 10 cells

The predesigned 25-nucleotide Dicer-substrate siRNAs targeting rat E-cadherin, N-cadherin, NuMA, LGN, 4.1R, 4.1G, RhoA, RhoB, RhoC, ROCK1, ROCK2, ARHGEF17, p120-catenin, ARVCF, p190B/ARHGAP5, and p190A/ARHGAP35 were purchased from Integrated DNA Technologies (IDT) and are listed in Table S4. Negative control DsiRNA (IDT, 51-01-14-04) was used throughout experiments. Can 10 cells plated at 0.9 × 10^4^/cm^2^ were transfected with 4.8 nM siRNA using Lipofectamine RNAiMAX Transfection Reagent (13778150; Thermo Fisher Scientific) and cultured for 72 h in Ham’s F-12K medium with 5% FBS.

For E-cadherin siRNA-rescue experiment, siRNA #1, which targets the 3′UTR of *Cdh1*, and pLJM1–GFP–E-cadherin, which contains only the open reading frame of *Cdh1*, were used. For ARHGEF17 siRNA-rescue experiment, 12 nucleotides of the *ARHGEF17* siRNA #2 targeting a region within the DH domain were mutated without altering the amino acid sequence as follows: 5′-**A**G**G**TA**C**GA**AT**T**AT**T**A**GT**C**AA**A**GA**TT**-3′ (the mutated nucleotides are in bold). The DH-deletion mutant ARHGEF17 ΔDH (1-1058/1248-2057) lacks the siRNA targeting region. The mutated genes were inserted into the lentiviral vector pLJM1-GFP. Lentivirus was packaged by cotransfecting E-cadherin–GFP, si#2-resistant ARHGEF17-GFP, or ARHGEF17 ΔDH-GFP with the packaging plasmids pMDLg/pRRE, pRSV-Rev, and pMD2.G into HEK293T cells using Lipofectamine 3000 transfection reagent (L3000015; Thermo Fisher Scientific). The medium was changed to DMEM containing 10% FBS 12 h after transfection, and the lentivirus-containing supernatant was collected at 48 h after transfection. The viral supernatants were filtered and then added to Can 10 cells that had been transfected with siRNA one day earlier, followed by culturing for 48 h.

### Immunofluorescence microscopy

Can 10 cells and Fao cells were cultured on 18-mm coverslips (EMS) coated with rat collagen type I (Corning) in Ham’s F-12K medium supplemented with 5% FBS. For Mdr1 staining, cells were permeabilized with acetone for 2 min at 4°C. For staining of Par3 and NuMA, cells were fixed in 100% methanol for 2 min at 4°C followed by 1.8% formaldehyde (sc-203049A; Santa Cruz Biotechnology) for 10 min. Fixed cells were washed twice with PBS (135 mM NaCl, 1.3 mM KCl, 3.2 mM Na_2_HPO_4_, 0.5 mM KH_2_PO_4_, pH 7.4), then blocked with 3% bovine serum albumin (BSA) in PBS for 30 min. For RhoA staining, cells were fixed in 10% trichloroacetic acid (TCA) for 20 min at 4°C. For other antibodies, cells were fixed with 1.8% formaldehyde for 10 min, washed twice with PBS, and permeabilized in PBS containing 0.1% Triton X-100 and 3% BSA. Indirect immunofluorescence analysis was performed using the following primary antibodies: mouse anti-E-cadherin (610182; BD Biosciences, 1:200), rabbit anti-E-cadherin (GTX100443; GeneTex, 1:200), mouse anti-N-cadherin (610921; BD Biosciences, 1:200), mouse anti-N-cadherin (sc-59987; Santa Cruz Biotechnology, 1:200), rabbit anti-N-cadherin (13116; Cell Signaling Technology, 1:250), rat anti-ZO-1 (sc-33725; Santa Cruz Biotechnology, 1:500), rabbit anti-ZO-1 (40-2200; Thermo Fisher Scientific, 1:2,000), mouse anti-Mdr1 (MA1-26528; Thermo Fisher Scientific, 1:100), rabbit anti-radixin (GTX105408; GeneTex, 1:250), mouse anti-aPKC (sc-17781; Santa Cruz Biotechnology, 1:200), rabbit anti-pericentrin (ab220784; Abcam, 1:1,000), rabbit anti-Par3 (07-330; Millipore, 1:1,000), mouse anti-NuMA (MABE1807; Millipore, 1:200), rabbit anti-EPB41L2/4.1G (ab175928; Abcam, 1:250), mouse anti-RhoA (sc-418; Santa Cruz Biotechnology, 1:200), mouse anti-p120-catenin (sc-23872; Santa Cruz Biotechnology, 1:200), rabbit anti-ARVCF (PA5-64129; Thermo Fisher Scientific, 1:200), rabbit anti-RhoB (63876; Cell Signaling Technology, 1:400), rabbit anti-ROCK1 (28999; Cell Signaling Technology, 1:400), and rabbit anti-ROCK2 (47012; Cell Signaling Technology, 1:200). The secondary antibodies and phalloidin used were as follows: Alexa Fluor 405–conjugated goat anti-rat IgG antibodies (ab175671; Abcam), Alexa Fluor 488–conjugated chicken anti-rabbit (A21441; Thermo Fisher Scientific) or goat anti-mouse IgG antibodies (A11001; Thermo Fisher Scientific), Alexa Fluor 555–conjugated goat anti-rabbit (A21428; Thermo Fisher Scientific) or goat anti-mouse IgG antibodies (A21422; Thermo Fisher Scientific), and Alexa Fluor 647–conjugated phalloidin (A22287; Thermo Fisher Scientific). Stained samples were washed twice with PBS and mounted with VECTASHIELD PLUS Antifade Mounting Medium (Vector Laboratories) or VECTASHIELD PLUS Antifade Mounting Medium with DAPI (Vector Laboratories). Confocal images were obtained using a spinning disk confocal scanner unit (CSU-X1; Yokogawa) with a Nikon Ti2-E microscope. The microscope was equipped with a CFI Plan Apo 20×/0.75 Lambda objective (Nikon), a CFI Plan 40×/1.30 oil-immersion objective (Nikon), a CFI Apo TIRF 100×/1.49 oil-immersion objective (Nikon), an ORCA-Quest qCMOS camera (C15550-20UP; Hamamatsu Photonics), and a Stradus VersaLase four-wavelength laser system (Vortran Laser Technology). The imaging system was controlled by VisiView (Visitron Systems).

For live-cell imaging of Can 10 cells, cells were grown on ibiTreat µ-slide or 35-mm µ-dish (ibidi) and imaged at 37°C in a humidified chamber (H301-K-FRAME; Okolab) with 5% CO_2_. Confocal images were acquired every 10 min with 28 z-stacks at a step size of 0.9 µm (Mzt1-GFP/radixin–mScarlet) or 26 z-stacks at a step size of 0.8 µm (dT-2xrGBD) using a spinning disk confocal scanner unit (CSU-X1; Yokogawa) attached to a Nikon Ti2-E microscope equipped with a CFI Plan 40×/1.30 oil-immersion objective (Nikon), an ORCA-Quest qCMOS camera (C15550-20UP; Hamamatsu Photonics) or an EMCCD camera (Evolve 512 Delta, Photometrics), and a Stradus VersaLase laser system (Vortran Laser Technology).

For imaging of mouse liver tissues and cultured explants, specimens were fixed in 4% paraformaldehyde at 4°C overnight, then dehydrated, and embedded in paraffin. The paraffin-embedded tissues were sliced into 10-µm-thick sections. After rehydration, antigen retrieval was performed by autoclaving the sections in citrate buffer (10 mM citrate, 0.05% Tween-20, pH 6.0) for 10 min. For RhoA staining, specimens were fixed in 10% TCA solution (R21475-500 ml; Shanghai Yuanye Bio-Technology Co., Ltd.) at 4°C for 1 h, washed three times with 30 mM glycine solution dissolved in PBS for 10 min each time, then placed in OCT, and rapidly frozen on dry ice. The frozen tissues were sliced into 10-µm-thick sections. Indirect immunostaining was performed using the following primary antibodies: goat anti-E-cadherin (AF748; R&D Systems, 1:100), goat anti-HNF4A (LS-C758303; LifeSpan BioSciences, 1:50), rat anti-EpCAM (17-5791-82; Thermo Fisher Scientific, 1:100), rat anti-DLK (D187-4; MBL, 1:100), mouse anti-MDR1 (sc-55510; Santa Cruz Biotechnology, 1:100), mouse anti-N-cadherin (14215; Cell Signaling Technology, 1:100), mouse anti-RhoA (sc-418; Santa Cruz Biotechnology, 1:25), rabbit anti-E-cadherin (20874-1-AP; Proteintech, 1:100), rabbit anti-albumin (16475-1-AP; Proteintech, 1:100), and rabbit anti-ZO-1 (61-7300; Invitrogen, 1:200). Nuclei were stained with DAPI (D9564; Sigma-Aldrich, 0.5 µg/ml). Images were captured with a TCS SP8 confocal microscope (Leica Microsystems) using a Plan-Apochromat 63×/0.75 NA objective lens (Leica Microsystems).

### Super-resolution imaging

Instant structured illumination microscopy (iSIM) images were acquired using a microscope (model Olympus IX71 inverted microscope, Evident Scientific) equipped with a UPlanSApo 60×/NA 1.2 water immersion objective (Evident Scientific), an ORCA-Quest qCMOS camera (model C15550-20UP; Hamamatsu Photonics), and a confocal scan head: VisiTech VT-iSIM (VisiTech International, Inc.). The imaging system was controlled by MetaMorph (Molecular Devices). Obtained images were further deconvolved using the Microvolution deconvolution plugin (Microvolution) in ImageJ.

STED microscopy images were captured with TCS SP8 STED 3× (Leica Microsystems) using a Plan-Apochromat 100×/1.40 NA oil-immersion STED white objective lens. Acquired mages were reconstructed using Huygens STED deconvolution software (Scientific Volume Imaging) according to the manufacturer’s protocol.

### Image processing and analysis

All images were processed and analyzed using Fiji/ImageJ (2.16.0/1.54 g). To quantify the intensity ratio of E-cadherin or N-cadherin on the polar cortex to AJ, the mean intensity of the polar membrane signal was divided by the mean intensity on AJ. Quantification of the intensity ratio of the cadherins on the basal cleavage furrow or on the basal surface surrounding the midbody to AJ was measured by calculating the ratio of the mean intensity of the indicated basal surface area divided by the mean intensity on AJ. For quantification of the NuMA intensity ratio of polar cortex to the cytoplasm, the mean intensity of the polar membrane signal was divided by the mean intensity in the cytoplasm area between a spindle pole and the polar cortex. Quantification of polar enrichment of NuMA was measured by calculating the ratio of the mean intensity of polar membrane signal divided by the mean intensity of the equatorial membrane signal. To quantify the intensity ratio of BC-associated RhoA or active RhoA vs. cytoplasm, the mean intensity of the apical membrane signal in hepatic polarity cells or the edge of the apical membrane in columnar polarity cells was divided by the mean intensity in the cytoplasm. High intensity area of rGBD on the edge of the apical membrane was defined as the region showing an intensity >1.0 relative to the cytoplasmic intensity. Quantification of the apical ROCK2 signal was measured by calculating the ratio of the mean intensity of the apical edge delineated by the aPKC signal divided by the mean intensity in the cytoplasm. All data analyzed for intensity measurements were from at least two independent experiments. For quantification of ZO-1 length, the length of the ZO-1–positive structure was measured. For measurement of spindle angles, the acute angle between the spindle axis and the line along the apical surface was measured. Quantification of cells with BCs was performed as previously described (Wang et al., 2014), with minor modifications. Briefly, cells exhibiting a radixin- or aPKC-positive apical membrane positioned between adjacent cells were classified as having hepatic polarity. BC structures were categorized as follows: a BC formed between 2 cells was defined as a primordial BC, while a BC enclosed by three or more cells and exhibiting a long-to-short axis ratio >2.0 was defined as a tubular BC. Cells with apical domains facing the culture surface were classified as having columnar polarity. The sum of cells with hepatic or columnar polarity was counted as apical–basal polarized cells. All quantifications of BC structures were based on at least four independent experiments.

### Reversible cross-link immunoprecipitation

The ReCLIP assay was performed as described by Smith et al. (2011), with minor modifications optimized for Can 10 cells. 2×10^6^ cells were cultured on a 10-cm dish for 72 h in Ham’s F-12K medium supplemented with 5% FBS. Cells were washed twice with Dulbecco’s phosphate-buffered salt solution containing Ca^2+^ and Mg^2+^ (DPBS; 21030CM; Corning) at room temperature (RT). After removal of DPBS, 10 ml of a 0.5 mM DSP cross-linker (22585; Pierce) solution in DPBS was added to each plate. Cells were incubated with the cross-linker for 30 min at RT with occasional agitation. Following addition of 10 ml quenching solution (20 mM Tris-Cl, pH 7.6), cells were incubated at RT for an additional 10 min. After quenching, cells were washed once with chilled DPBS and lysed with RIPA buffer (150 mM NaCl, 1 mM Na_2_EDTA, 1 mM EGTA, 1% NP-40, 1% sodium deoxycholate, 2.5 mM Na_4_P_2_O_7_, 1 mM β-glycerophosphate, 1 mM Na_3_VO_4_, 1 µg/ml leupeptin, 20 mM Tris-Cl, pH 7.5; 9806; Cell Signaling Technology) containing protease inhibitor cocktail (P8340; Sigma-Aldrich). Lysates were homogenized by pipetting 10 times and cleared by centrifugation. The cell lysate was incubated with protein G Sepharose (17061801; Cytiva) conjugated to an anti-GFP antibody (50430-2-AP; Proteintech), a negative control mouse IgG (X0931; Agilent), an anti-E-cadherin antibody (610182; BD Biosciences), or an anti-N-cadherin antibody (sc-59987; Santa Cruz Biotechnology) for 2 h at 4°C with end-over-end rotation. After the beads were washed three times with RIPA buffer supplemented with protease inhibitors, the precipitants were eluted with 2× Laemmli sample buffer (1610747; Bio-Rad) containing the reducing agents 20% 2-mercaptoethanol and 50 mM DTT.

### Proximity labeling assay

The proximity labeling assay was performed as described by Cho et al. (2020), with modifications optimized for cadherin expression in Can 10 cells. To generate lentiviruses, HEK293T cells were cultured in 10-cm dishes and transfected with 3.2 µg of the lentiviral vector containing the gene of interest and the lentiviral packaging plasmids pMDLg/pRRE (0.8 µg), pRSV-Rev (0.8 µg), and the VSV-G envelope–expressing vector pMD2.G (2.5 µg) using Lipofectamine 3000 transfection reagent (Thermo Fisher Scientific). After 48 h, the cell medium containing the lentivirus was harvested and filtered through a 0.45-μm filter. 3 × 10^6^ Can 10 cells were infected with the crude lentivirus and cultured in biotin-free medium (Dulbecco’s modified Eagle’s medium supplemented with 10% FBS). For biotin labeling of transduced cells, biotin was added 2 days after infection. Biotin (100 mM stock in DMSO) was diluted in the biotin-free medium and added to the cells to a final concentration of 50 µM, followed by incubation at 37°C for 16 h. Labeling was stopped by gentle washing 10 times with chilled Dulbecco’s phosphate-buffered saline containing Ca^2+^ and Mg^2+^. The supernatant was removed, and the pellet was lysed by resuspension in RIPA buffer (150 mM NaCl, 1 mM Na_2_EDTA, 1 mM EGTA, 1% NP-40, 1% sodium deoxycholate, 2.5 mM Na_4_P_2_O_7_, 1 mM β-glycerophosphate, 1 mM Na_3_VO_4_, 1 µg/ml leupeptin, 20 mM Tris-Cl, pH 7.5; 9806; Cell Signaling Technology) containing protease inhibitor cocktail (P8340; Sigma-Aldrich) and incubated for 10 min at 4°C. Lysates were clarified by centrifugation at 15,000 *g* for 20 min at 4°C. For enrichment of biotinylated proteins, 400 µg streptavidin-coated magnetic beads (PI88816; Pierce) were washed twice with RIPA buffer and incubated with clarified lysates for 2 h at 4°C with end-over-end rotation. The beads were then washed twice with 1 ml RIPA buffer, once with 1 ml 1 M KCl, once with 1 ml 0.1 M Na_2_CO_3_, once with 1 ml 2 M urea in 10 mM Tris-Cl (pH 8.0), and twice with 1 ml RIPA buffer. After enrichment, biotinylated proteins were eluted by boiling the beads in 40 μl 2× Laemmli sample buffer (1610747; Bio-Rad) supplemented with 20 mM DTT and 2 mM biotin. The eluted proteins were separated by SDS-PAGE gel for further processing and preparation for LC-MS/MS analysis.

### LC-MS/MS analysis and data processing

Eluted proteins were separated on an SDS gel for ∼5 mm, fixed, and stained using Colloidal Blue Staining Kit (LC6025; Thermo Fisher Scientific). The entire region of the gel-containing protein was excised, reduced with TCEP, alkylated with iodoacetamide, and digested with trypsin. The resulting tryptic digests were analyzed using a Q Exactive Plus mass spectrometer (Thermo Fisher Scientific) coupled with the Vanquish UHPLC system (Thermo Fisher Scientific). Mass spectrometry data were searched with full tryptic specificity against the UniProt rat proteome database (07/21/2022) and a contaminant database using MaxQuant 2.4.2.0. Variable modifications searched include the following: acetylation on protein N terminus, oxidation on methionine, and deamidation on asparagine. Protein quantification was performed using razor + unique peptides. Common contaminants, incorrect identifications, and proteins identified by only a single razor + unique peptide were removed from the protein list. Protein and peptide abundance was measured using intensity-based absolute quantification, followed by log_2_ transformation for normalization prior to limma analysis. Differential analysis was performed using the R package limma (v3.64.1) with the parameter “trend = TRUE.” Significant protein identifications between E-cadherin and N-cadherin samples were defined as those showing: (1) minimum fold change (experimental/control) >2 and (2) adjusted P value <0.05. Protein accession numbers were converted to gene symbols using UniProt for subsequent gene ontology enrichment analysis. The BioID interactome datasets for shared, E-cadherin–unique, and N-cadherin–unique proteins are provided in Tables S1, S2, and S3, respectively.

### Immunoblot analysis

Can 10 cells and Fao cells were washed with ice-cold DPBS containing Ca^2+^ and Mg^2+^ (21030CM; Corning) and lysed in 2× Laemmli sample buffer (1610747; Bio-Rad) containing 1× protease inhibitor cocktail (P8340; Sigma-Aldrich) and 10% 2-mercaptoethanol. Western blotting was performed following standard protocols, using the following primary antibodies: anti-E-cadherin (610182; BD Biosciences, 1:500), anti-N-cadherin (13116; Cell Signaling Technology, 1:200), anti-pan-cadherin (71-7100; Thermo Fisher Scientific, 1:1,000), anti-catenin δ-1/p120-catenin (59854; Cell Signaling Technology, 1:1,000), anti-RhoA (sc-418; Santa Cruz Biotechnology, 1:250), anti-ARVCF (PA5-64129; Thermo Fisher Scientific, 1:250), anti-GFP (50430-2-AP; Proteintech, 1:500), anti-NuMA1 (MABE1807; Millipore, 1:400), anti-LGN (ABT174; Millipore, 1:1,500), anti-4.1R (sc-166759; Santa Cruz Biotechnology, 1:200), anti-EPB41L2/4.1G (ab175928; Abcam, 1:1,000), anti-p190-A RhoGAP (2860; Cell Signaling Technology, 1:500), anti-ARHGAP5/p190B (MA5-38043; Thermo Fisher Scientific, 1:500), anti-RhoA (2117; Cell Signaling Technology, 1:1,000), anti-RhoB (63876; Cell Signaling Technology, 1:1,000), anti-RhoC (3430; Cell Signaling Technology, 1:1,000), anti-ROCK1 (28999; Cell Signaling Technology, 1:1,000), anti-ROCK2 (47012; Cell Signaling Technology, 1:1,000), and anti-α-tubulin (66031-1-Ig; Proteintech, 1:2,000). For quantification of E-cadherin and N-cadherin intensity, the membranes were incubated with IRDye 800CW goat anti-mouse (926-32210; LI-COR Biosciences) and IRDye 680RD goat anti-rabbit secondary antibodies (926-68071; LI-COR Biosciences) diluted at 1:2,000 in Antibody Diluent (Thermo Fisher Scientific) at RT for 1 h. After three washes with 1× wash buffer (Thermo Fisher Scientific), the blots were scanned with ODYSSEY M (LI-COR Biosciences). For other antibodies, Rabbit and Mouse Optimized HRP reagents (Thermo Fisher Scientific) were used as secondary antibodies. The blots were developed with SuperSignal West Femto Maximum Sensitivity Substrate (34096; Thermo Fisher Scientific) after three washes with the wash buffer, followed by scanning with LI-COR ODYSSEY M. The obtained images were analyzed with Image Studio (LI-COR Biosciences).

For isolation of mouse hepatoblasts and hepatocytes, embryonic mouse liver tissues were digested with a mixture of collagenases (2.5 mg/ml collagenase II, 2.5 mg/ml collagenase IV, dissolved in RPMI 1640 medium) at 37°C. The cells were labeled with an anti-mouse DLK-FITC (D187-4; MBL International, 1:100) antibody, filtered through a 70-µm cell strainer, and sorted using a BD FACSAria Fusion cell sorter (BD Biosciences). Dead cells were excluded using DAPI staining. The targeted mouse cells were lysed in 2× loading buffer [5× loading buffer (WB2001; NCM Biotech) diluted in strong RIPA lysis solution (50 mM Tris [pH 7.4], 150 mM NaCl, 1% Triton X-100, 1% sodium deoxycholate, 0.1% SDS)]. Western blotting was performed following the standard protocol using the following primary antibodies: anti-E-cadherin (610182; BD Biosciences, 1:1,000), anti-N-cadherin (14215; Cell Signaling Technology, 1:1,000), anti-pan-cadherin (71-7100; Thermo Fisher Scientific, 1:1,000), and anti-β-actin (AC026; ABclonal, 1:5,000). For quantification of E-cadherin and N-cadherin intensity, the membranes were incubated with IRDye 800CW goat anti-mouse (926-32210; LI-COR Biosciences) and IRDye 680RD goat anti-rabbit secondary antibodies (926-68021; LI-COR Biosciences) diluted at 1:5,000 at RT for 1 h. After three washes with PBS containing 0.1% Tween-20, the blots were scanned with ChemiDoc MP Imaging System (Bio-Rad). The obtained images were analyzed with Fiji/ImageJ (2.16.0/1.54 g).

### Quantification and statistical analysis

All statistical analyses were performed using RStudio (ver. 2024.12.0+467). All tests were two-sided. For parametric analyses, data distribution was assumed to be normal but was not formally tested due to limited sample size. Individual data points are shown where applicable. For cell culture experiments, independent experiments represent biological replicates consisting of separately prepared cultures that were independently transfected, treated, fixed, stained, imaged, and quantified. For ex vivo liver culture experiments, independent experiments represent biological replicates consisting of separately prepared liver explants. Data are presented as the mean ± SD or as box-and-whisker plots, as specified in the figure legends. Quantification of polarity types and BC structures between two groups was analyzed by Welch’s *t* test, and comparisons among more than two groups were performed using pairwise *t* tests with Holm’s correction. ReCLIP-coprecipitated levels of E- or N-cadherin were compared using Welch’s *t* test. Protein expression levels between control and knockdown cells were analyzed by pairwise *t* tests with Holm’s correction. ZO-1–positive structure lengths in Fao cells expressing mScarlet vs. E-cadherin–mScarlet were compared using the Wilcoxon rank-sum test. Spindle angles and intensity ratios were compared between two groups using the Wilcoxon rank-sum test; comparisons among more than two groups were performed using Wilcoxon’s rank-sum tests with Holm’s correction.

### Online supplemental material

Fig. S1 shows expression profiles of DLK1, albumin, EpCAM, HNF4α, E-cadherin, and N-cadherin in hepatoblasts and hepatocytes during mouse liver development, and expression of E-cadherin, N-cadherin, and p120-catenin in Can 10 cells. Fig. S2 shows shared and distinct roles of E- and N-cadherin in polarity development and BC formation in Can 10 cells. Fig. S3 shows identification of E- and N-cadherin interactors; roles of NuMA and its interactors LGN and Band 4.1 in BC elongation; and E-cadherin function in spindle orientation. Fig. S4 shows BC inheritance during cytokinesis; functionality of the Rho biosensor (d-2×rGBD) in Can 10 cells; and expression, localization, and knockdown efficiency of Rho and ROCK isoforms in Can 10 cells. Fig. S5 shows localization and function of ARHGEF17 in relation to E-cadherin; roles of E-cadherin and ARHGEF17 in RhoA activation; and roles of RhoA and ARHGEF17 in spindle orientation. Table S1 shows BioID interactome data of shared proteins between E-cadherin and N-cadherin. Table S2 shows BioID interactome data of E-cadherin–unique proteins. Table S3 shows BioID interactome data of N-cadherin–unique proteins. Table S4 shows plasmids and siRNA sequences used in this study. Video 1 shows time-lapse analysis of Can 10 cells expressing the active RhoA biosensor (dT-2×rGBD) and containing a preexisting BC, showing RhoA activation dynamics at the BC and at the division site during cytokinesis-mediated BC elongation. Video 2 shows time-lapse analysis of ARHGEF17-GFP and mScarlet–β-actin localization in Can 10 cells before and after LatA treatment, showing the effect of F-actin disruption on ARHGEF17 localization at AJs and the cell cortex. Video 3 shows time-lapse analysis of Can 10 cells expressing the active RhoA biosensor (dT-2×rGBD) and containing a preexisting BC, transfected with control siRNA (left) or ARHGEF17 siRNA (right), illustrating that ARHGEF17 is required for RhoA activation at the BC but not at the midbody.

## Supplementary Material

Table S1 shows BioID interactome data of shared proteins between E-cadherin and N-cadherin.

Table S2 shows BioID interactome data of E-cadherin–unique proteins.

Table S3 shows BioID interactome data of N-cadherin–unique proteins.

Table S4 shows plasmids and siRNA sequences used in this study.

SourceData F1 is the source file for Fig. 1.

SourceData F2 is the source file for Fig. 2.

SourceData F3 is the source file for Fig. 3.

SourceData F4 is the source file for Fig. 4.

SourceData F5 is the source file for Fig. 5.

SourceData F8 is the source file for Fig. 8.

SourceData F9 is the source file for Fig. 9.

SourceData FS1 is the source file for Fig. S1.

SourceData FS3 is the source file for Fig. S3.

SourceData FS4 is the source file for Fig. S4.

SourceData FS5 is the source file for Fig. S5.

## Data Availability

The data supporting the findings of this study are included in the paper and its supplemental information and are available from the primary corresponding author, Erfei Bi (ebi@pennmedicine.upenn.edu), upon reasonable request.
