# Supplementary figures and images for: E- and N-cadherin drive hepatic polarity and lumen elongation via opposing effects on RhoA activity
Source: J Cell Biol. 2026 May 27;225(8):e202509170. doi: 10.1083/jcb.202509170 (PMC13215057; doi:10.1083/jcb.202509170)

Figure 1 E

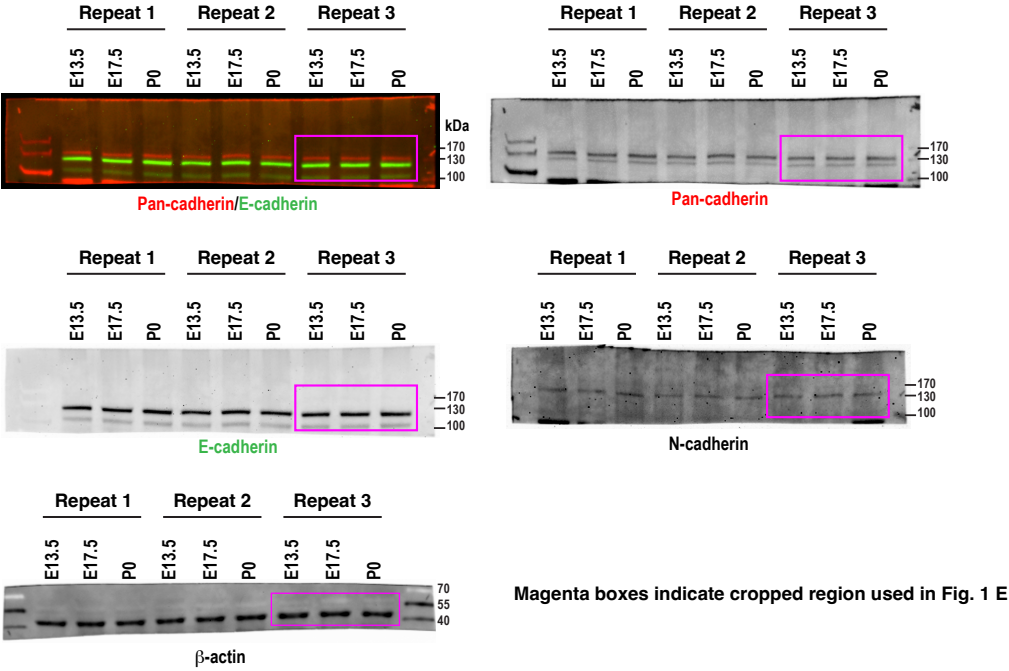

Magenta boxes indicate cropped region used in Fig. 1 E

Supplement: SourceData F1 — is the source file for Fig. 1. [file jcb_202509170_sourcedataf1.pdf]

Figure 2 B

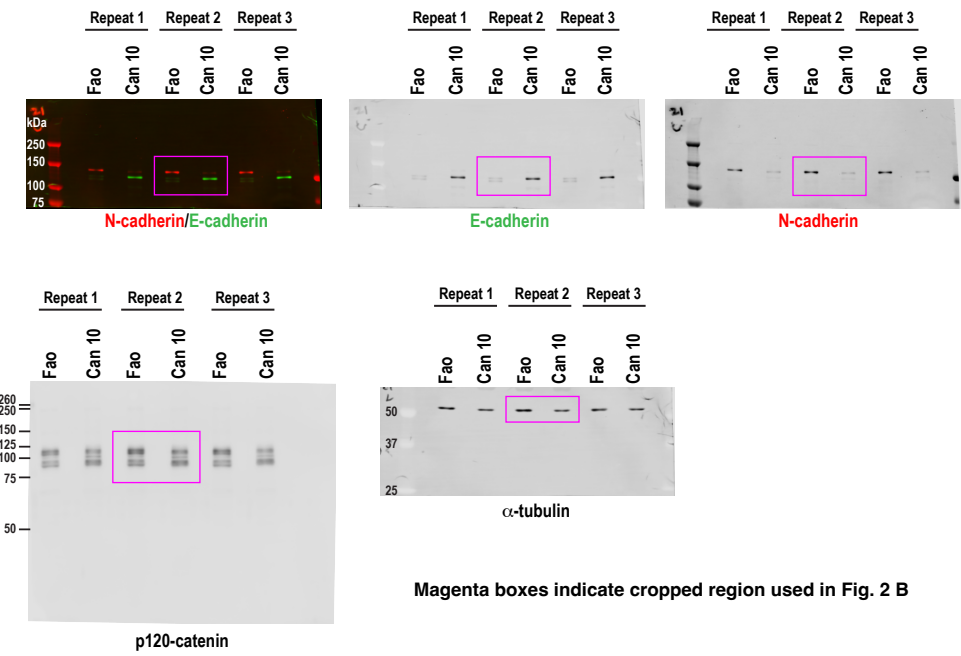

Supplement: SourceData F2 — is the source file for Fig. 2. [file jcb_202509170_sourcedataf2.pdf]

Figure 4 D

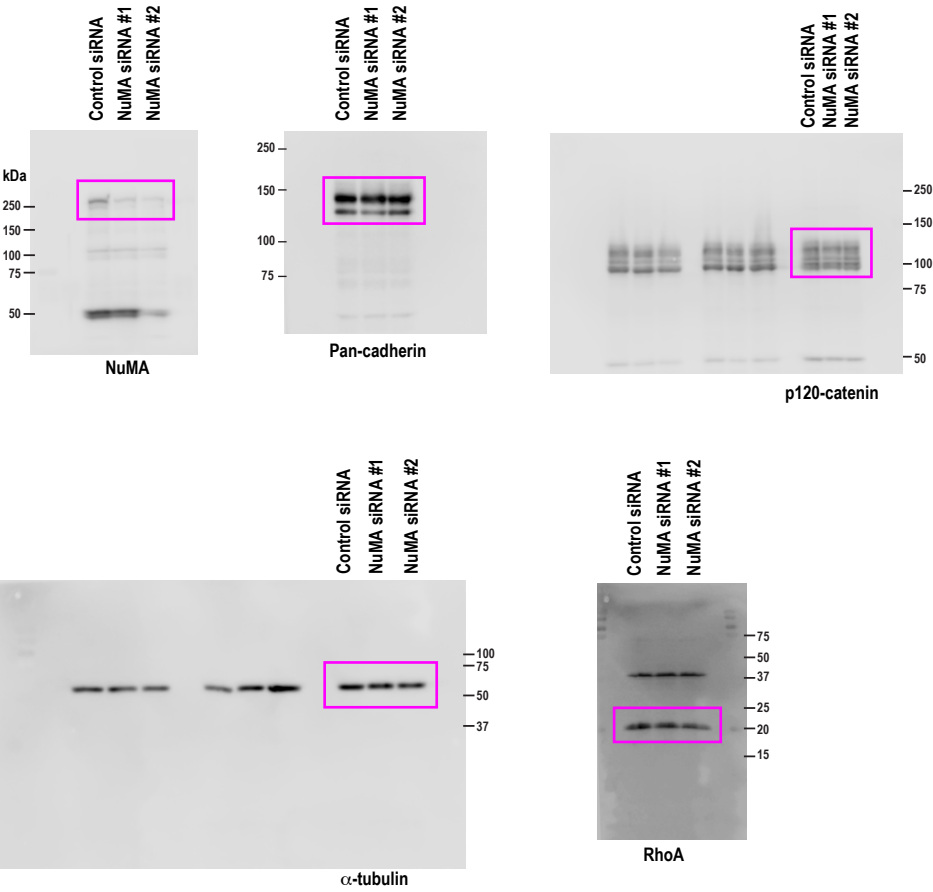

Magenta boxes indicate cropped region used in Fig. 4 D

Supplement: SourceData F4 — is the source file for Fig. 4. [file jcb_202509170_sourcedataf4.pdf]

Figure 5

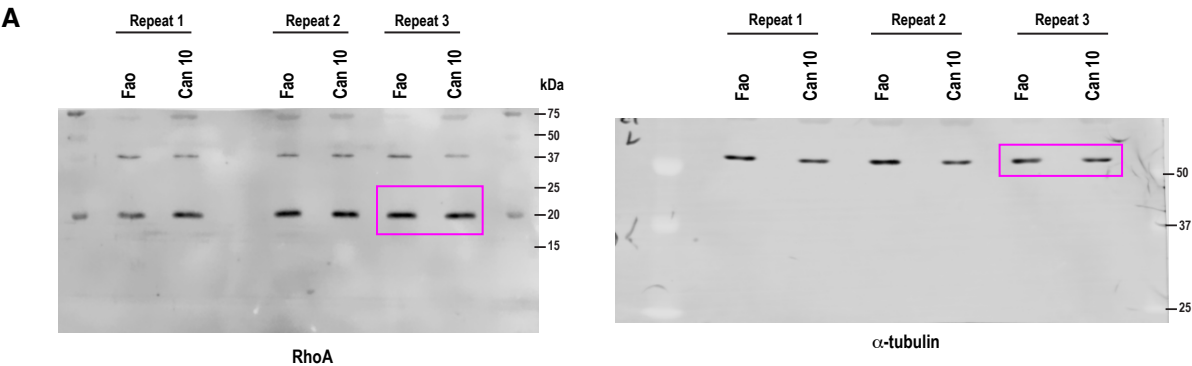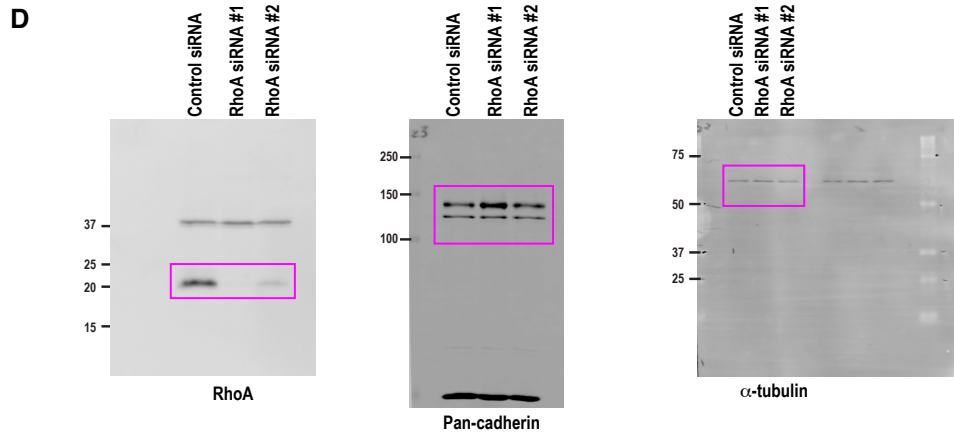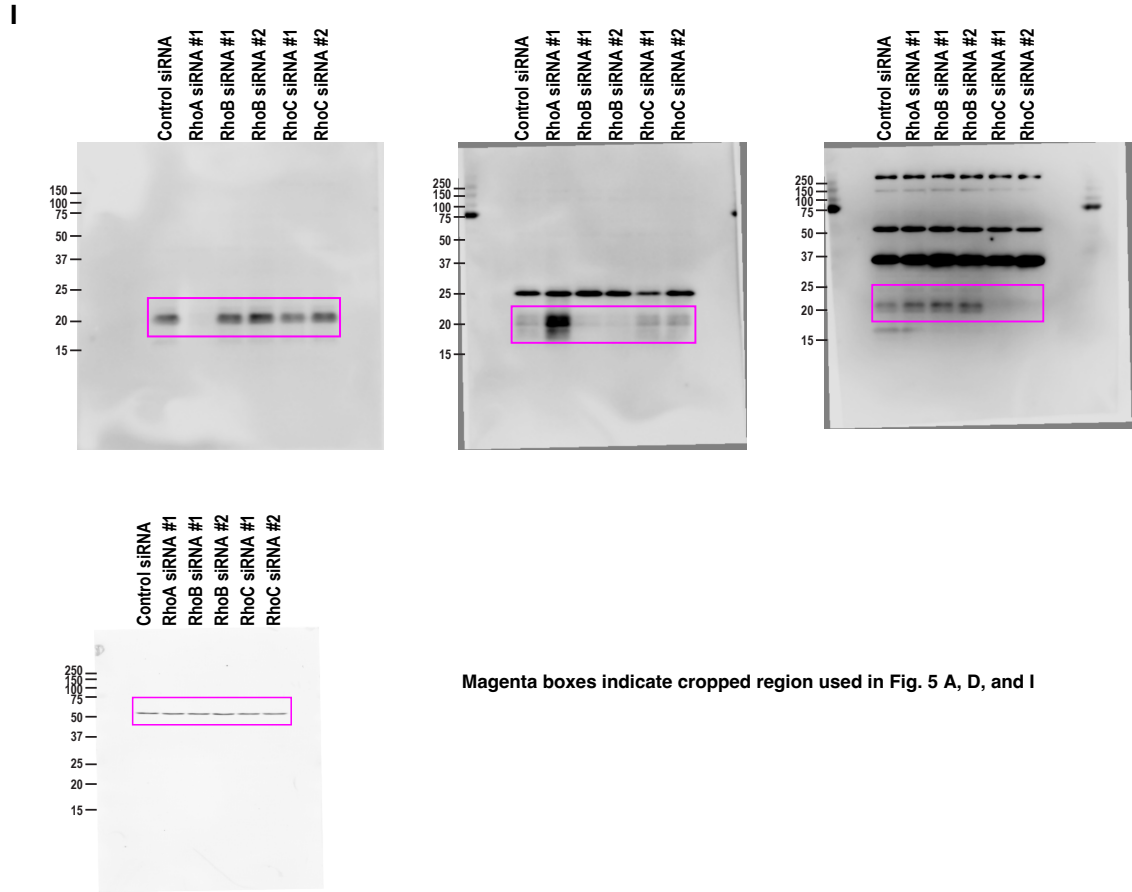

Supplement: SourceData F5 — is the source file for Fig. 5. [file jcb_202509170_sourcedataf5.pdf]

Figure 8 C

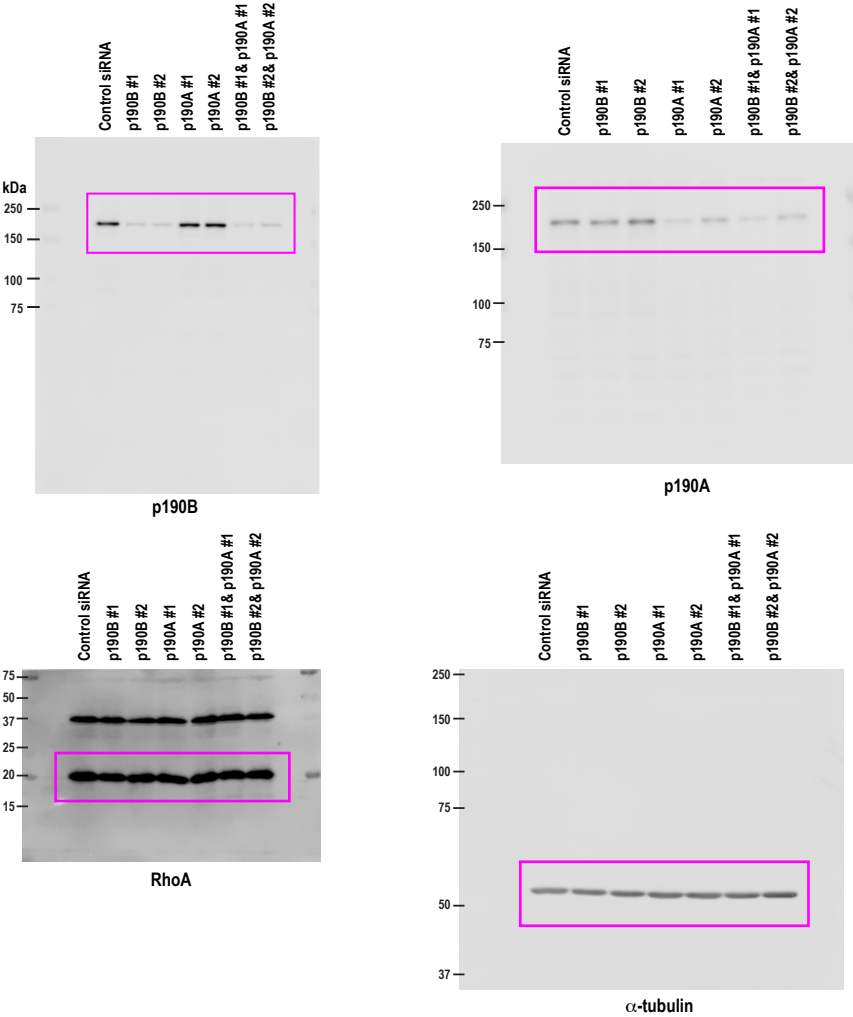

Magenta boxes indicate cropped region used in Fig. 8 C

Supplement: SourceData F8 — is the source file for Fig. 8. [file jcb_202509170_sourcedataf8.pdf]

Figure 9

B

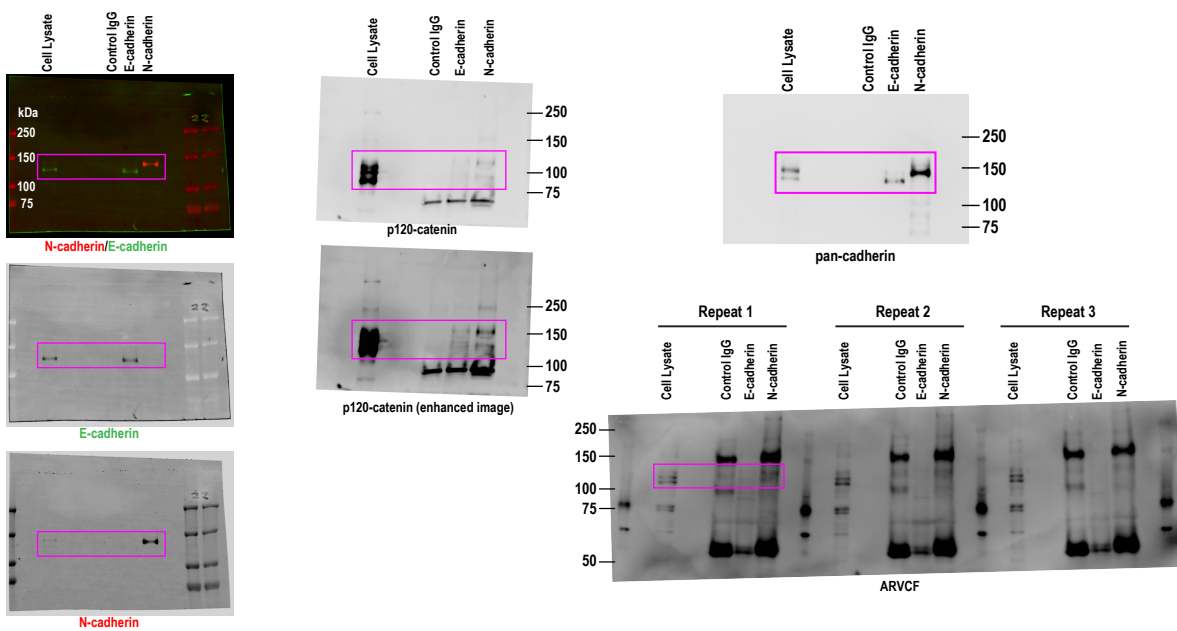

D

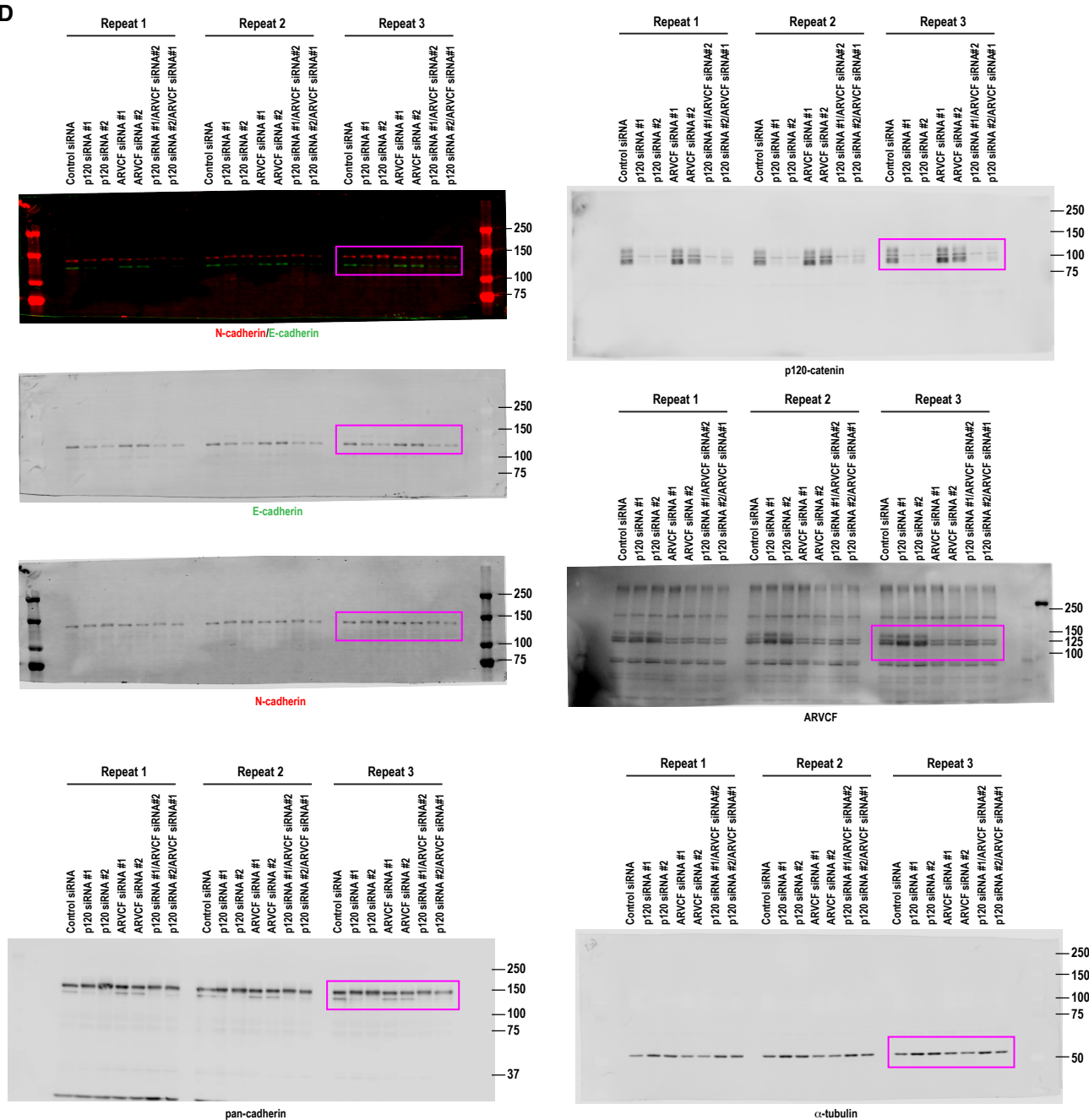

Magenta boxes indicate cropped region used in Fig. 9 B and D

Supplement: SourceData F9 — is the source file for Fig. 9. [file jcb_202509170_sourcedataf9.pdf]

Figure S1

F

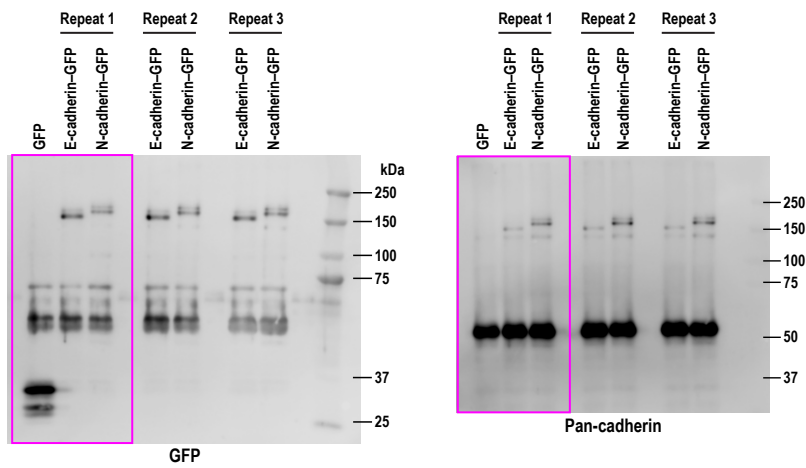

G

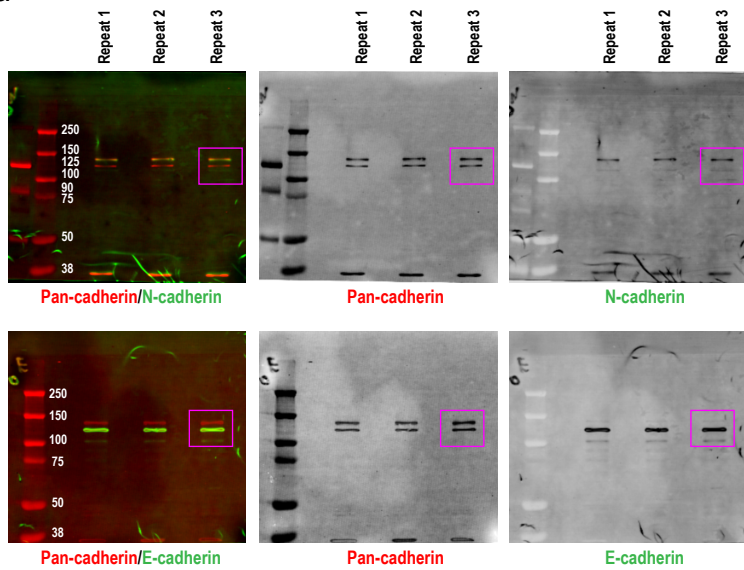

H

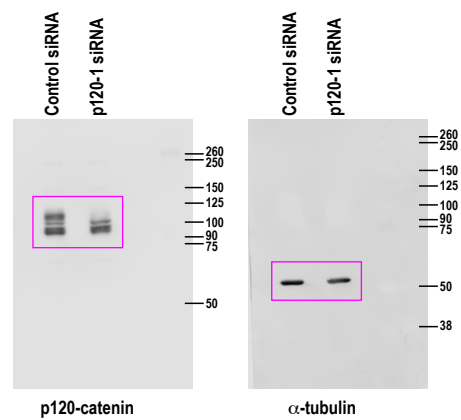

Magenta boxes indicate cropped region used in Figure S1 F, G, and H

Supplement: SourceData FS1 — is the source file for Fig. S1. [file jcb_202509170_sourcedatafs1.pdf]

Figure S3

A

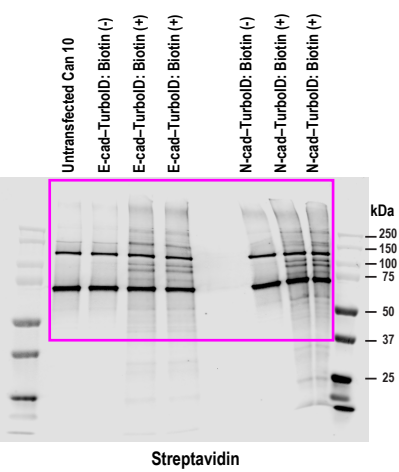

E

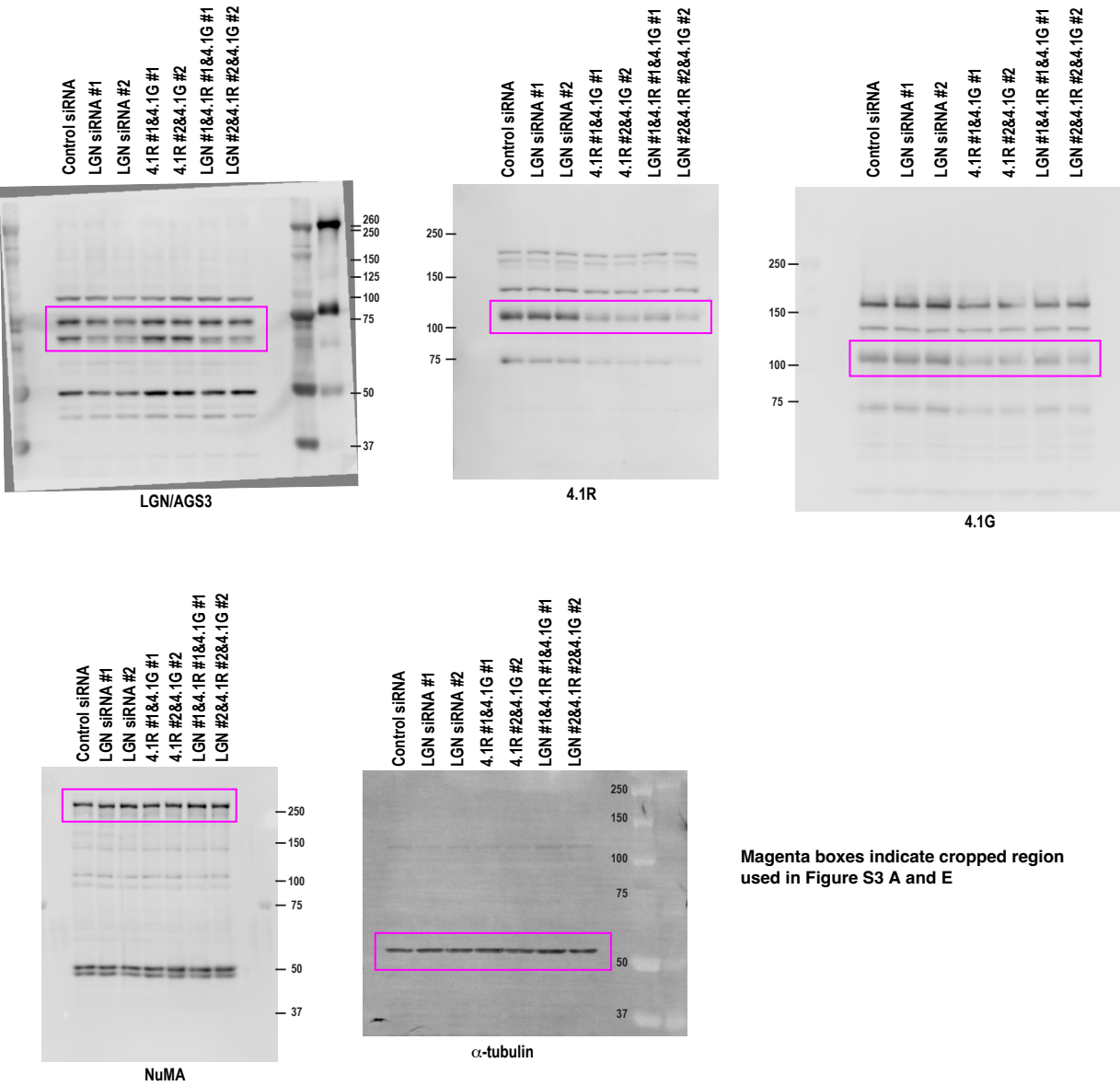

Supplement: SourceData FS3 — is the source file for Fig. S3. [file jcb_202509170_sourcedatafs3.pdf]

Figure S4 E

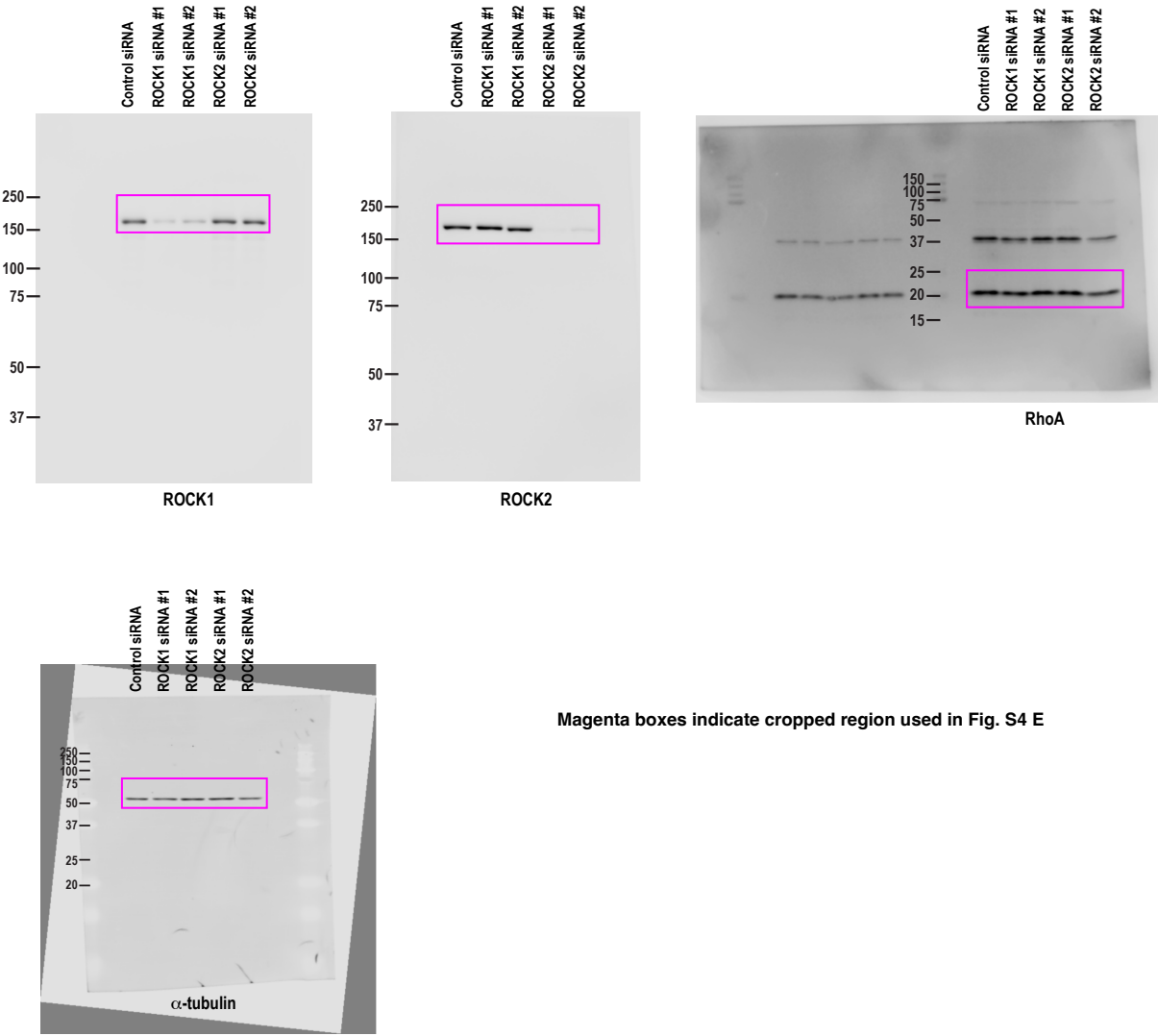

Supplement: SourceData FS4 — is the source file for Fig. S4. [file jcb_202509170_sourcedatafs4.pdf]

Figure S5

I

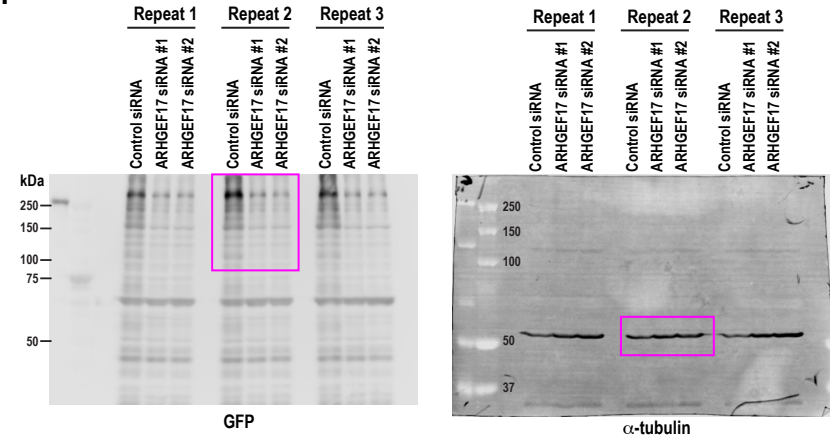

J

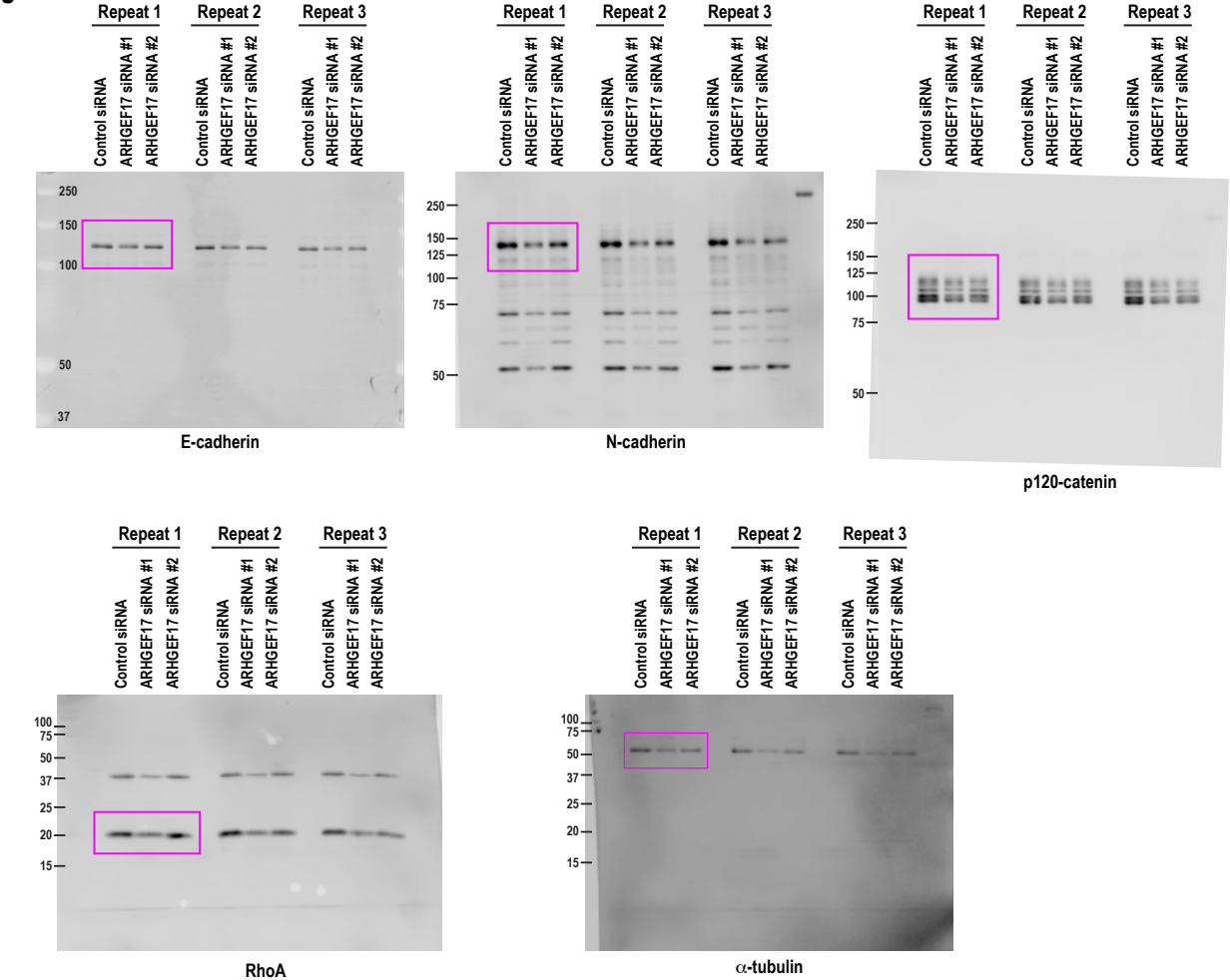

Magenta boxes indicate cropped region used in Fig. S5 I and J

Supplement: SourceData FS5 — is the source file for Fig. S5. [file jcb_202509170_sourcedatafs5.pdf]
