# Supplementary material for: E- and N-cadherin drive hepatic polarity and lumen elongation via opposing effects on RhoA activity
Source: J Cell Biol. 2026 May 27;225(8):e202509170. doi: 10.1083/jcb.202509170 (PMC13215057; doi:10.1083/jcb.202509170)
Supplement: SourceData F3 — is the source file for Fig. 3. [file jcb_202509170_sourcedataf3.pdf]

Figure 3

A & E

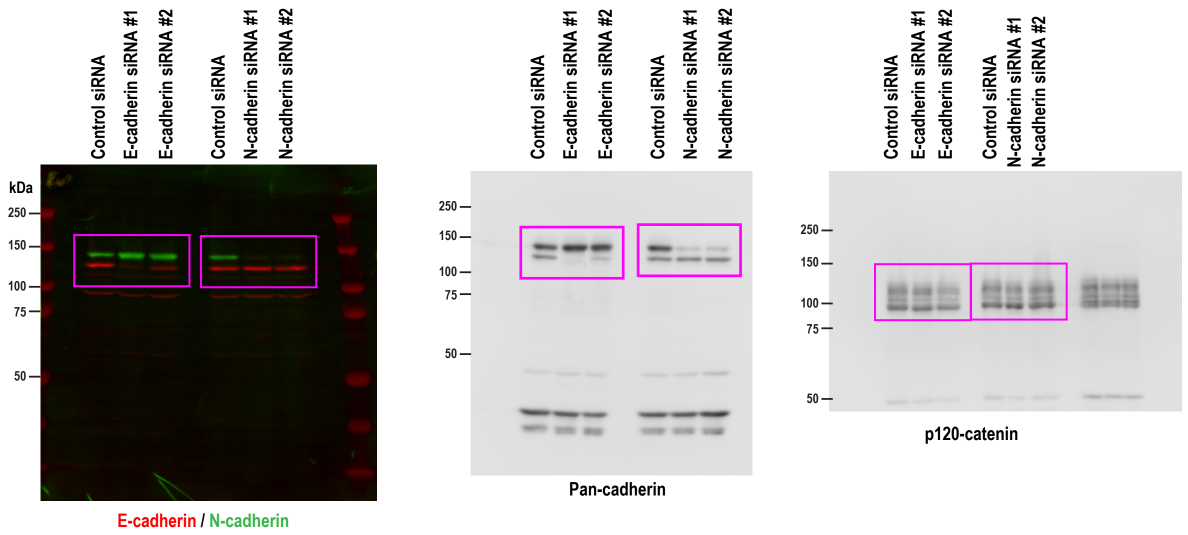

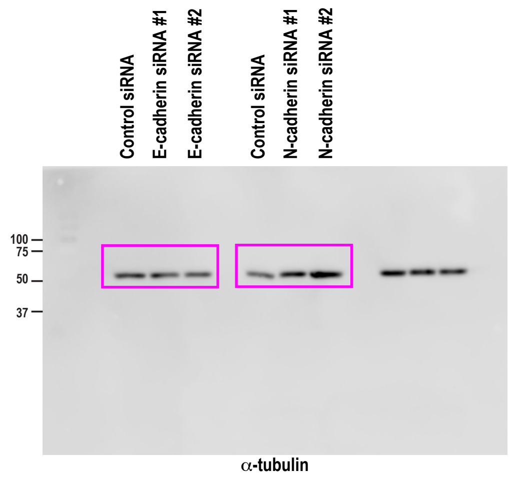

Magenta boxes indicate cropped region used in Fig. 3 A and E

H

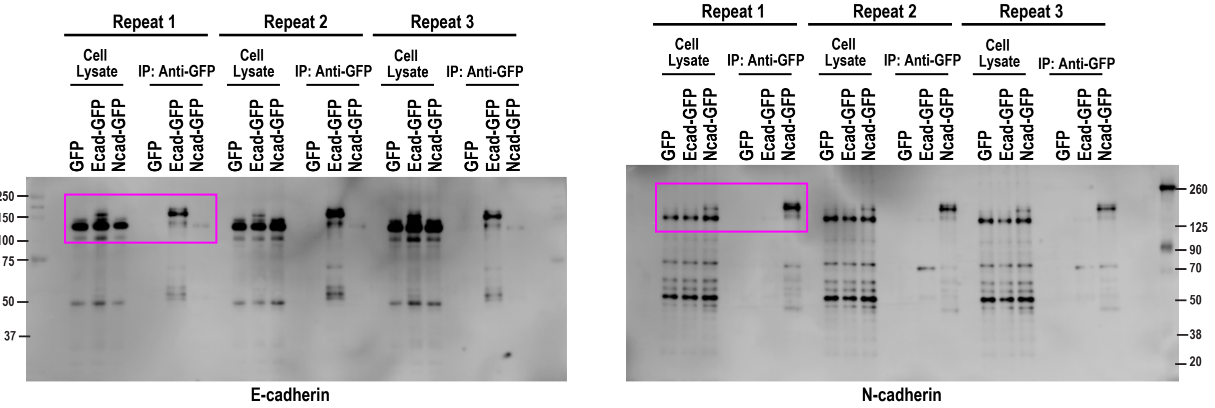

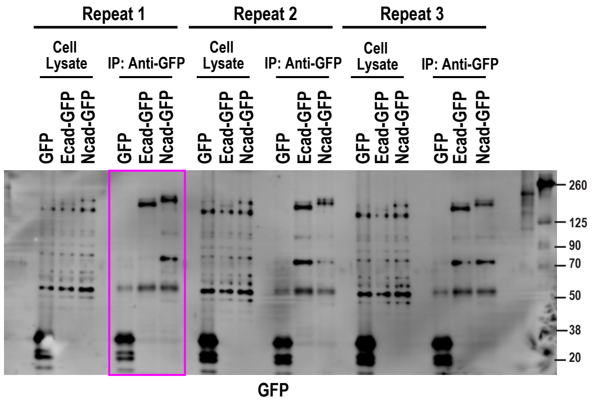

Magenta boxes indicate cropped region used in Fig. 3 H
